# Biomaterial-based extracellular vesicle delivery systems for wound healing: From fabrication to applications

**DOI:** 10.1016/j.bioactmat.2026.06.042

**Published:** 2026-08-05

**Authors:** Shengqiu Chen, Zhiliang Zhao, Lige Tian, Shengnan Cui, Xi Liu, Hao Meng, Yi Xie, Dawei Chen, Xingwu Ran, Changsheng Zhao, Xiaobing Fu, Cuiping Zhang

**Affiliations:** a Innovation Research Center for Diabetic Foot, West China Hospital, Sichuan University, Chengdu, 610041, China; b Talent Office, West China Hospital, Sichuan University, Chengdu, 610041, China; c Medical Innovation Research Department, Chinese PLA General Hospital and PLA Medical College, Beijing, 100048, China; d College of Polymer Science and Engineering, State Key Laboratory of Polymer Materials Engineering, Sichuan University, Chengdu, 610065, China; e Department of Endocrinology and Metabolism, Diabetic Foot Care Center, West China Hospital, Sichuan University, Chengdu, 610041, China

**Keywords:** Extracellular vesicle, Delivery system, Biomaterials, Release mechanism, Wound healing

## Abstract

Skin damage, especially chronic wounds, imposes a significant clinical burden due to the associated risks of disability and death. Extracellular vesicles (EVs) have received extensive attention due to their potential in wound healing. However, the rapid clearance and short retention time of EVs at wound sites hinder the optimal therapeutic effects. The latest advancements in biomaterial delivery systems have spurred the rapid development of engineered EV delivery systems. These biomaterials can not only prolong the localization and stability of EVs in wounds, but also provide structural and biochemical support for cell growth and tissue regeneration. This review provides a comprehensive overview of EVs and the fabrication strategies of EV delivery systems with diverse application forms. The release mechanisms, release profiles, mathematical kinetic models of EVs from biomaterials are also discussed. The application of these EV-loaded biomaterials in treating various types of wounds is also summarized and discussed. Finally, current challenges and future directions in EV-based wound treatment are proposed. Unlike previous reviews that focused separately on the biology of EVs or biomaterials as wound dressings, this review presents a comprehensive summary of the fabrication strategies and applications of advanced biomaterial-based EV delivery platforms for wound healing.

## Highlights

• This review summarizes the basic information of EVs and obstacles to direct application in wound healing.
• The different fabrication techniques of biomaterial-based EV delivery platforms with various forms are also summarized.
• The release mechanisms of EVs from biomaterials are summarized.
• The latest achievements of EV-loaded biomaterials in treating various types of wounds are systematically discussed.

## Graphical abstract

**Figure:**
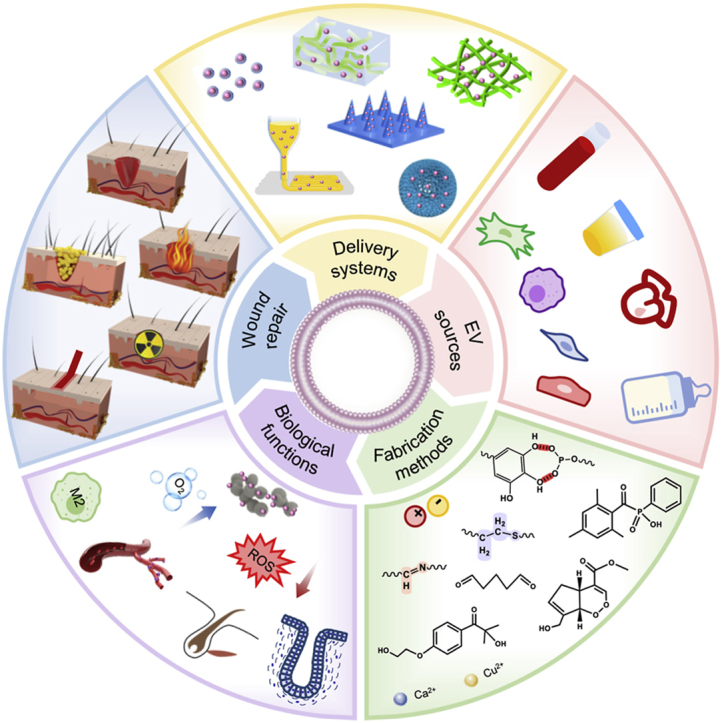

## 1 Introduction

Skin injury is a key clinical issue, especially chronic wounds, such as diabetic ulcers [[1], [2], [3]], infected trauma [[4], [5], [6]], burn damage [[7], [8], [9]], radiation-induced skin injuries [[10], [11], [12]], and compromised skin flaps [[13], [14], [15]]. These wounds impose a considerable burden on patients and healthcare systems. Wound healing is a dynamic and multi-stage process, consisting of four overlapping stages: hemostasis, inflammatory, proliferative, and remodeling, and involves the interaction among various types of cells, cytokines and extracellular matrix (ECM) [16,17]. Extracellular vesicles (EVs) have become a promising “cell-free” therapeutic strategy in promoting wound healing, owing to their good biocompatibility, low cytotoxicity, minimal immunogenicity, specific targeting ability, and the ability to cross biological barriers [[18], [19], [20]]. Currently, EVs can be mammalian-driveded in (MEVs), bacterial-derived EVs (BEVs), and plant-derived EVs [21]. Among them, MEVs are nanoscale phospholipid bilayer vesicles [22] and rich in functional molecular cargoes, including proteins, lipids, metabolites, DNA, and various types of RNAs [23,24]. These bioactive molecules contribute to intercellular communication and regulate key cellular processes in wound healing, such as improving the functions of endothelial cells [25,26], fibroblasts [27], and keratinocytes [28,29]. The therapeutic effect of EVs depends on their cellular source [[30], [31], [32]], thus emphasizing the necessity of selecting the source of EV for specific wound types. Numrous studies have demonstrated that EVs exhibit diverse biological functions in skin wound repair, such as antioxidant [33], anti-inflammation [34], neovascularization [35], re-epithelialization and regeneration of skin appendages, such as hair follicle growth [[36], [37], [38]] and sweat gland restoration [39].

**Fig. 1:**
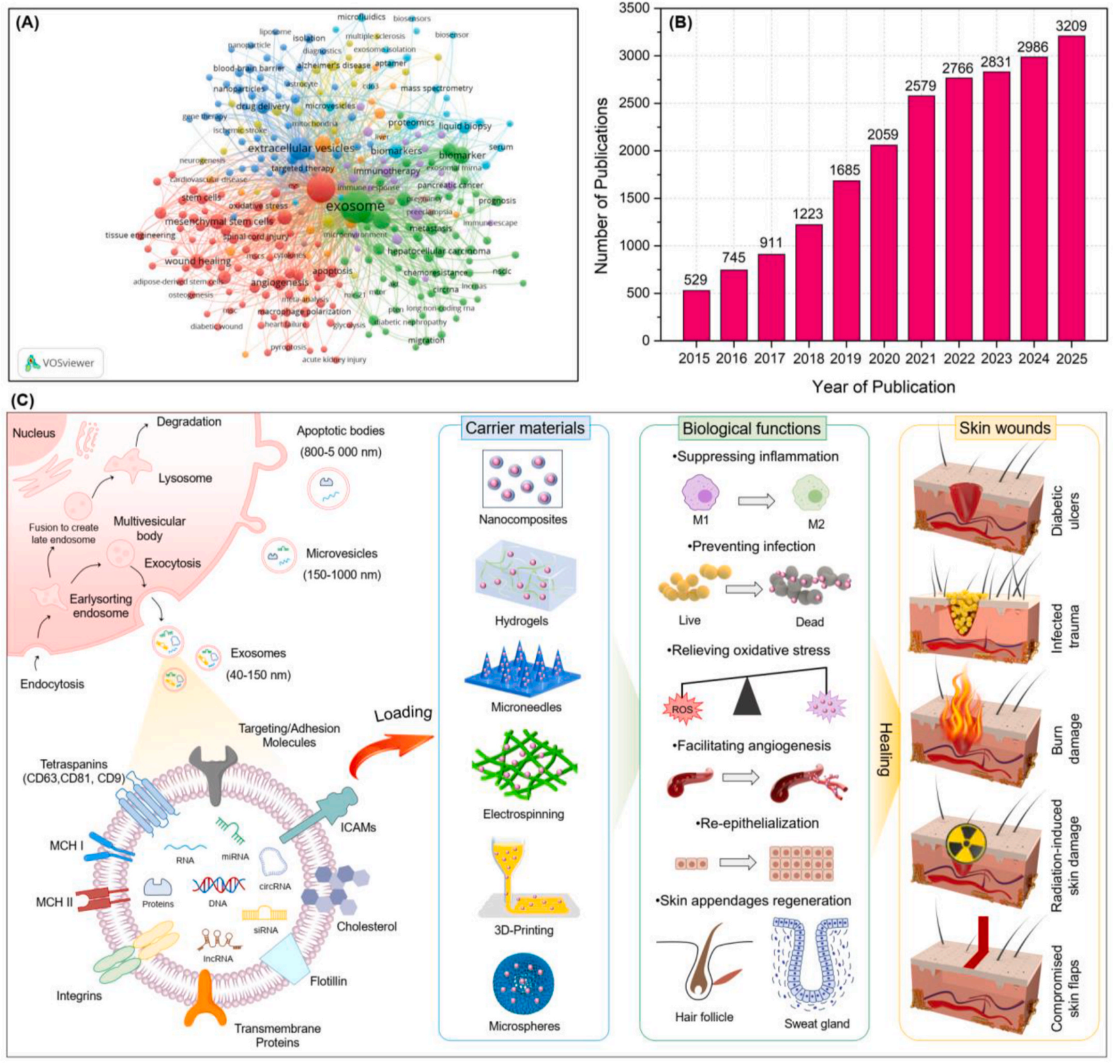
(A) Author keyword co-occurrence network visualization analysis created by VOSviewer software (version VOSviewer 1.6.20) with top keywords extracting from over 21, 523 previously published articles (data obtained December 5, 2025) related to “exosome” or “extracellular vesicle” and “wound” in the Web of Science Core Collection Database. (B) Number of publications on EVs and wounds published in the last 10 years according to Web of Science Core Collection Database (the keywors are “exosome” or “extracellular vesicle” and “wound”). (C) Schematic illustration of biogenesis of EV subtypes and the EV-loaded biomaterial platforms with a variety of forms such as nanocomposites, hydrogels, microneedle patches, electrospun membranes, 3D-printed scaffolds, and microspheres, as well as their biological functions for the repair of various skin wounds.

A bibliometric analysis was performed using VOSviewer software on literature retrieved from the Web of Science Core Collection Database, including 21, 523 publications related to “exosome” or “extracellular vesicle” and “wound”. From 27, 027 extracted keywords, 309 terms occurred at least 30 times. As shown in Fig. 1A, these keywords were clustered into five major groups: cluster 1 (red nodes, focusing on EV-based wound therapeutics), cluster 2 (purple nodes, centering on the immune regulation mediated by EVs), cluster 3 (blue nodes, highlighting EV-based drug delivery systems), cluster 4 (green nodes, focusing on the EV biogenesis and biomarkers), cluster 5 (yellow nodes, covering neurogenesis, neuroinflammation, neurodegeneration, neuroprotection related to EVs). The growing research interest in exosome or EVs for wound repair over the past decade highlights the significant opportunities for future exploration (Fig. 1B).

One major challenge for EVs application is to achieve long retention time at wound sites [40]. Currently, EVs are primarily administered through local injection or intravenous infusion into wound sites [[41], [42], [43]], which in turn impede their therapeutic efficacy, such as short half-life, rapid clearance from circulation, limited bioactivity, and inadequate targeting [44,45]. To overcome these limitations, combining EVs with biomaterials has emerged as a promising strategy to protect EVs from immune clearance in vivo, thereby prolonging their retention time and improving therapeutic effects [[46], [47], [48]]. In recent years, various biomaterial-based delivery systems have been developed to enhance the therapeutic effect of EVs, including nanocomposites [49], hydrogels [50], microneedle patches [51], electrospun membranes [52,53], 3D-printed scaffolds [54], microspheres [55], and sponges [56,57], as illustrated in Fig. 1C. With the assistance of biomaterials, these advanced EV delivery systems have demonstrated significant therapeutic potential in wound healing through stimulating multiple regenerative mechanisms, mainly including: inhibiting bacterial infection [58], reducing inflammation [59,60], relieving oxidative stress [61,62], rescuing microvascular dysfunction [63,64], inhibiting ferroptosis [65], alleviating tissue hypoxia [66], maintaining a moist wound environment [67], providing a good mechanical environment [68], and supporting skin appendage regeneration to achieve functional tissue repair [69]. By synergistically integrating these mechanisms, this comprehensive strategy effectively accelerates wound healing, promotes functional skin reconstruction, and provides a promising approach for advanced wound management.

Despite the promising therapeutic potential of biomaterials carrying EVs, several critical obstacles impede their clinical translation. Firstly, inherent heterogeneity of EVs in their size and cargoes results in poor batch-to-batch reproducibility. Secondly, efficient loading of therapeutic cargoes without compromising EV integrity is still technically challenging. Thirdly, EVs suffer from rapid clearance by the mononuclear phagocyte system and insufficient target tissue accumulation. Fourthly, the release mechanism of EVs from biomaterials are not yet fully elucidated. Finally, the scalable Good Manufacturing Practice (GMP)-compliant production and real-time quality control are largely missing. This review aims to systemically summarize various biomaterial platforms carrying EVs for wound healing and identify clinically viable EV delivery systems.

In this review, we systematically summarize the sources and biological activity of EVs (derived from body fluids and mammalian cells) in wound healing. Additionally, we provide a comprehensive summary of the different preparation strategies of EV-loaded biomaterials, which can be engineered into various application forms (nanocomposites, hydrogels, microneedle patches, electrospun membranes, 3D-printed scaffolds, and microspheres). The release mechanisms, release profiles, and mathematical kinetic models of EVs from biomaterials are also summarized and discussed. Moreover, this review highlights the recent progress in the application of EV-loaded biomaterials for the management of various skin wounds (diabetic ulcers, infected trauma, burn damage, radiation-induced skin injuries, and compromised skin flaps). Furthermore, the clinical translation hurdles of EV-loaded biomaterials, primarily including compliance with cGMP for scalable production, reproducibility, long-term storage stability, regulatory considerations, and potential immunogenicity, are also discussed. Finally, we discuss the current limitations and future perspectives of EV-loaded biomaterial platforms in skin wound treatment.

## 2 Overview of EVs in wound healing

### 2.1 Overview of EVs

#### 2.1.1 Discovery of EVs

The foundational discovery of EVs dates back to 1983, Harding and Johnstone observed the release of transferrin receptor-associated vesicles from maturing sheep reticulocytes through receptor-mediated endocytosis and recycling pathways [70]. This seminal finding was subsequently formalized in 1987 when Johnstone first coined the term “exosomes” to describe these nanoscale vesicles [71]. The transport system and precise regulation mechanism of EVs discovered by James E. Rothman, Randy W. Schekman, and Thomas C. Südhof in 2013 was awarded the Nobel Prize in Physiology or Medicine [72], significantly advancing cellular and regenerative medicine. As we all know, miRNA is the content of EVs and also an important signaling molecule. Excitedly, “miRNA” discovered by Victor Ambros and Gary Ruvkun was awarded the Nobel Prize in Physiology or Medicine in 2024 [73]. These milestone events have significantly enhanced our understanding of EVs in intercellular communication and simultaneously catalyzed the exploration of their translational potential in clinical applications.

#### 2.1.2 Sources of EVs

EVs play key roles in physiological and pathological processes. These extracellular lipid bilayer vesicles are secreted by almost all mammalian cell types and detected in a variety of body fluids. EV-secreted mammalian cells are primarily categorized into two groups: (1) stem cells, including hADSCs [[74], [75], [76], [77], [78]], hBMSCs [[79], [80], [81], [82]], hiPSCs [[83], [84], [85]], hUCMSCs [[86], [87], [88], [89]], EpiSCs [90,91], AMSCs [92,93], FDMSCs [94], ESCs [95], USCs [96]; (2) differentiated cell types, such as macrophages [[97], [98], [99], [100]], fibroblasts [101,102], keratinocytes [103,104], and endothelial cells [105,106]. In contrast to stem cells, stem cell-derived EVs exhibited several advantages, including low risk of differentiation, immunogenicity and tumorigenicity, as well as no ethical concerns [107]. EVs are detected in the varying body fluids, primarily including peripheral blood or whole blood [41,108], umbilical cord blood [109,110], platelet-rich plasma (PRP) [[111], [112], [113]], urine [114], sweat [115], saliva [116], milk [117,118], and chronic wound fluid [119].

#### 2.1.3 Isolation and purification of EVs

EVs can be isolated by a variety of methods, like ultrafiltration, ultracentrifugation, tangential flow filtration, size-exclusion chromatography, precipitation method, and immunoaffinity capture method [120]. Nevertheless, their clinical application is restricted by the time-consuming isolation processes, limited purity, and low yields. To overcome these challenges, several commercial exosome isolation kits have been exploited, such as ExoQuick-TC Reagent (System Biosciences, CA, USA) [[121], [122], [123]], ExoSpin Purification Kit (Cell Guidance System, Cambridge, UK) [124,125], Hieff® Quick Exosome Isolation kit (Yesen, Shanghai, China) [80,126], Ribo™ Exosome Isolation Reagent (RiboBio, Guangzhou, China) [[127], [128], [129]], Exosome Isolation Kit (NovoBiotechnology, Beijing, China) [130], ExoEasy Maxi Kit (Qiagen, Frankfurt, Germany) [[131], [132], [133]], Total Exosome Isolation reagent (Invitrogen™, MA, USA) [127,134], and PureExo® Column (101Bio company, CA, USA) [135].

### 2.2 EV-mediated wound healing

#### 2.2.1 Biological functions of EVs

**Fig. 2:**
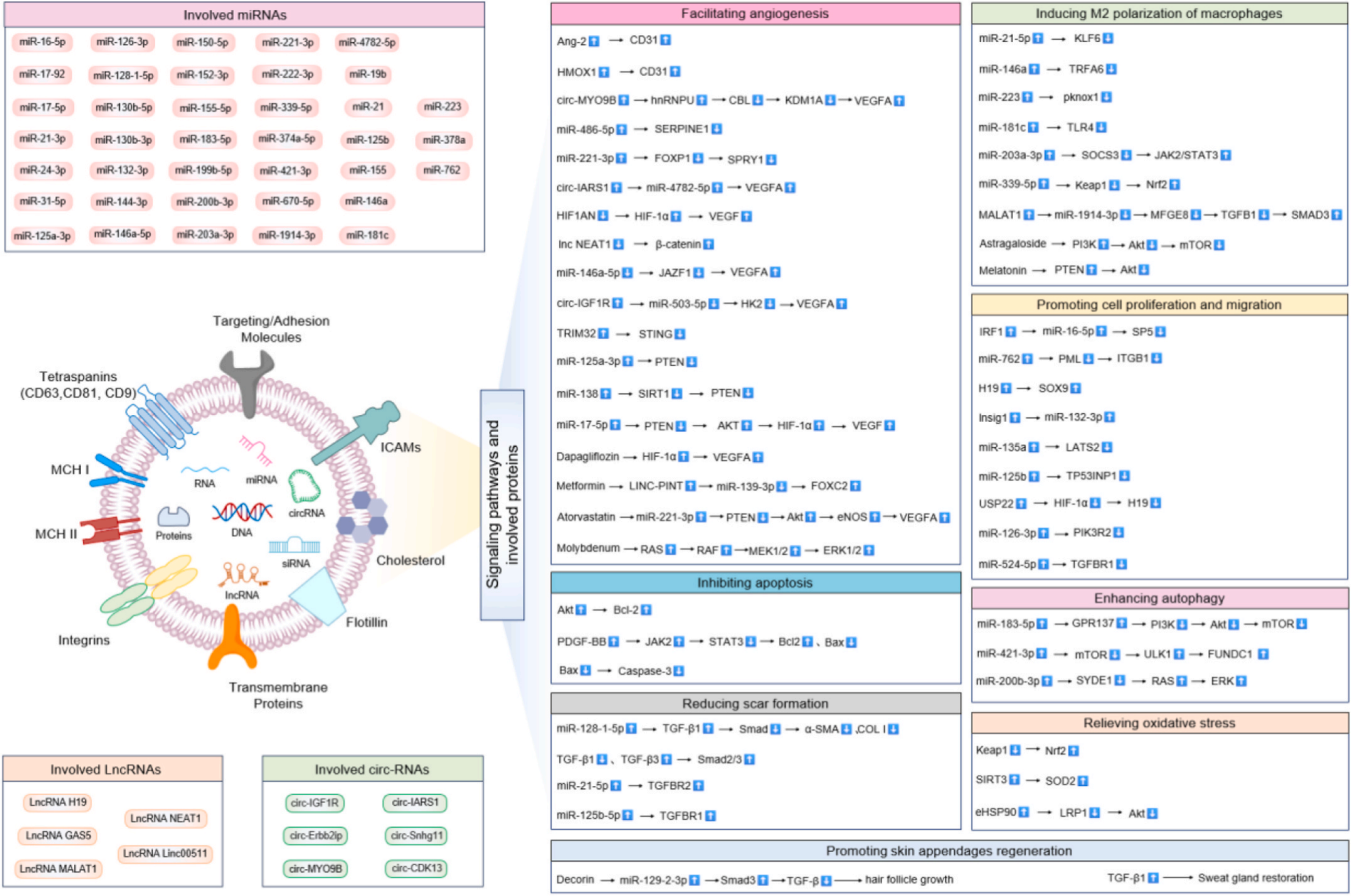
Schematic illustration of the biological functions of EVs in skin wound healing, mainly including (i) relieving oxidative stress, (ii) inducing M2 polarization of macrophages, (iii) promoting cell proliferation/migration, (iv) facilitating angiogenesis, (v) inhibiting apoptosis, (vi) enhancing autophagy, (vii) reducing scar formation, and (viii) promoting the regeneration of skin appendages.

EVs are rich in numerous functional molecules, such as proteins, lipids and nucleic acids [136]. Among these molecules, RNAs, particularly long noncoding RNAs (lncRNAs), microRNAs (miRNAs) and circular RNAs (circRNAs) [137], serve as pivotal regulators of cellular signaling pathways in cutaneous wound healing [138,139]. Some studies have demonstrated that EVs containing specific lncRNAs (such as MALAT1 [140,141], H19 [[142], [143], [144]], NEAT1 [145], GAS5 [146]) present a significant impact on the wound healing process through multiple mechanisms. In skin wound healing, as summarized in Fig. 2, Fig. S1 and Table S1, EVs orchestrate critical biological functions through the following pathways: (i) relieving oxidative stress; (ii) inducing M2 polarization of macrophages; (iii) promoting cell proliferation/migration; (iv) facilitating angiogenesis; (v) inhibiting apoptosis; (vi) enhancing autophagy; (vii) reducing scar formation; and (viii) promoting skin appendage regeneration.

#### 2.2.2 Obstacles of EVs in wound healing

**Fig. 3:**
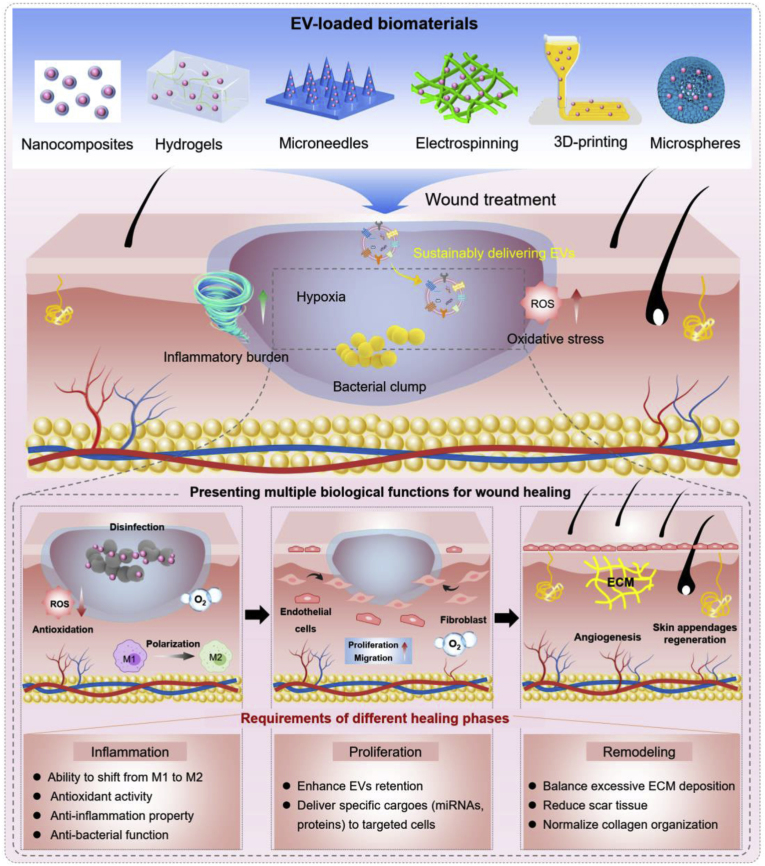
Schematic illustration of wound healing processes by EV-laden biomaterials.

EV therapeutics have demonstrated considerable application prospects in wound healing due to their minimal toxicity, low immunogenicity, and low tumorigenicity [147,148]. Nevertheless, conventional methods, such as local injection of free EV suspensions, have limited their therapeutic effects due to their rapid clearance and short in vivo half-life [149]. To overcome these challenges, recent strategies have focused on designing advanced biomaterial-based delivery platforms [[150], [151], [152]], to stabilize and continuously deliver EVs to wound sites [[153], [154], [155]]. These EV-loaded biomaterials can also provide structural and biochemical cues to support cellular processes necessary for wound healing. As illustrated in Fig. 3, this integrated platforms can address multiple pathological factors related to wound healing: preventing bacterial infections, regulating inflammation, reducing oxidative stress, promoting cell proliferation and migration, relieving hypoxia, stimulating angiogenesis, improving collagen deposition, and promoting the regeneration of skin appendages.

## 3 Biomaterial-based EV delivery platforms

Four primary strategies are widely utilized to integrate EVs into biomaterials, including physical encapsulation, surface adsorption, electrostatic interaction, and chemical immobilization [156]. For physical encapsulation, EVs are directly trapped inside 3D porous scaffold networks, where their diffusion is affected by the steric hindrance of the scaffolds. This method preserves EV structural integrity and biological activity without destructive processing [157]. Surface adsorption allows straightforward EV attachment to biomaterial surfaces via hydrophobic interactions and hydrogen bonding, requiring no complex chemical modification on EVs or scaffolds. For electrostatic interactions, EVs can be bind to polycation-modified scaffolds (like chitosan, polyethyleneimine, and positively-charged bacterial cellulose) [[158], [159], [160]] through electrostatic interactions between the phosphate groups on the lipid bilayer of EVs and positively-charged cationic polyelectrolytes. Chemical immobilization realizes specific, stable EV conjugation through covalent modification and affinity binding. For instance, a streptomycin-modified chitosan oligosaccharide-GelMA hydrogel forms a bridge with DSPE-PEG^2000^-Biotin-modified exosomes (Bio-Exos) with ultra-high affinity [161]. This “biotin-streptavidin” system achieves high-affinity EV capture and sustained release for long-term biological function. Additionally, choline phosphate (CP)-phosphatidylcholine (PC) pairing strategy enables strong specific binding to exosome membranes, offering a precise platform for EV delivery [162].

The advanced biomaterial-based EV delivery systems encompass a variety of application forms, mainly including nanocomposites, hydrogels, microneedle patches, electrospun membranes, 3D-printed scaffolds, and microspheres. In order to identify the most promising biomaterial platform for specific clinical scenarios, we summarized the advantages, limitations, and comparative efficacy of the abovementioned biomaterial platforms in delivering EVs for wound healing Tables S2 and S3. The following subsections will focus on the fabrication strategies of different forms of advanced biomaterials carrying EVs.

### 3.1 EV-integrated nanocomposites

Native EVs inherently have limited therapeutic components or targeting capabilities, thereby constraining their therapeutic potential. To enhance the therapeutic effect, EVs can be engineered on demand. Two primary methods are employed in engineering EVs: (i) endogenous loading: where host cells are pre-treated with therapeutic agents prior to isolation; and (ii) exogenous modification: where EVs are initially isolated from host cells, followed by modification with therapeutic agents onto EVs membrane through physiochemical interactions.

#### 3.1.1 Endogenous loading

**Fig. 4:**
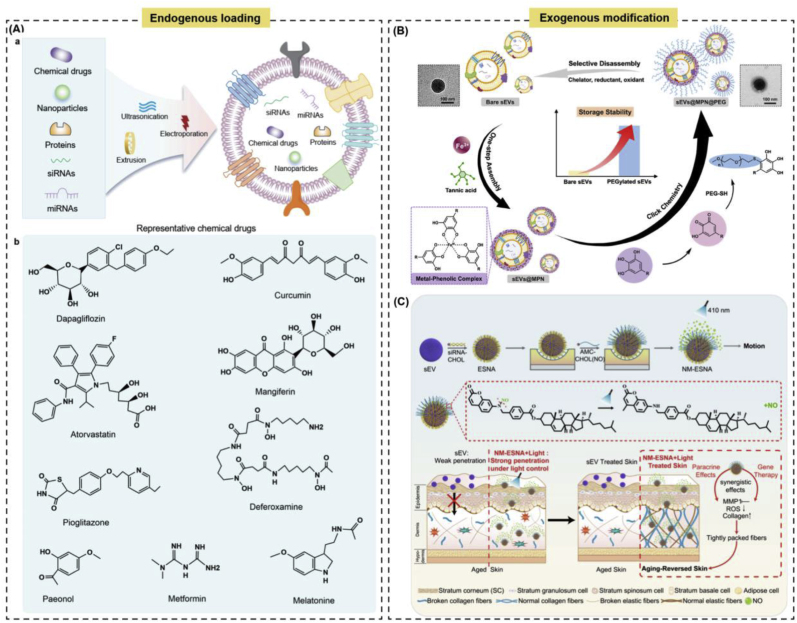
**Representative fabrication strategies for EV-integrated nanocomposites**. (A) (a) Endogenous loading methods including ultrasonication, extrusion and electroporation, (b) chemical structures of representative chemical drugs for engineering EVs. (B) Schematic diagram illustrating the encapsulation of sEVs within MPN coatings and subsequent surface PEGylation to protect the sEVs during storage, followed by the disassembly of the modified MPN coatings to generate functional sEVs. Reproduced with permission [163]. Copyright 2025, Wiley-VCH. (C) Schematic illustration of constructing a NM-ESNA with noninvasive, light-controlled, and enhanced transdermal delivery to the skin. Reproduced with permission [164]. Copyright 2025, American Chemical Society.

EVs, as promising natural nano-carriers, possess inherent capability to transport a diverse array of bioactive molecules or integrate with therapeutic agents, such as chemical drugs, nanoparticles, proteins, siRNAs and miRNAs, as illsutrated in Fig. 4A(a). For the fabrication of engineered EVs, many strategies such as extrusion, ultrasonication and electroporation are efficient and popular technologies. Particularly for chemical drugs, EVs have been commonly used to deliver them with poor water soluability. The representative chemical drugs, such as deferoxamine (DFO) [165], dapagliflozin [166], mangiferin [167], metformin (Met) [168], melatonin [169], curcumin (Cur) [170], pioglitazone [171], atorvastatin [172], and paeonol [173], have been reporetd for preparing EV-integrated nanocomposites. Their chemical structures are presented in Fig. 4A(b). As a representative case, Zhang and colleagues fabricated ESIONPs@EXO complexes by co-incubating bone marrow derived macrophages (BMMs) with extremely small iron oxide nanoparticle (ESIONPs) through multistep centrifugation [174]. They found that ESIONPs@EXO demonstrated the ability to target pathological angiogenesis via independent of VEGF pathway, instead involving the induction of ferroptosis.

#### 3.1.2 Exogenous modification

Exogenous modification of EVs is where EVs are initially isolated from host cells, followed by modification with therapeutic agents onto EVs membrane through physiochemical interactions. For instance, Qian and co-workers loaded angiogenic drug DFO into apoptotic body nanovesicles to form (DFO-nABs) through multi-step processes, involving optimizing hypotonic treatment, mild ultrasound, drug mixing and extrusion [165]. In vitro, they found that DFO-nABs could deliver DFO to promote endothelial cell proliferation, migration, and tube formation. In vivo studies further confirmed the efficacy of DFO-nABs in facilitating diabetic wound healing with rapid wound closure and enhanced neovascularization. To improve the colloidal stability of sEVs and enhance their resilience under harsh storage conditions, Ejima and colleagues modified the surface of sEVs with a metal-phenolic network (MPN) shell through a one-step assembly of tannic acid-Fe^3+^ coordination complexes, and further functionalized MPN-coated sEVs undergo surface PEGylation through Michael addition between residual phenolic groups in the MPN and the thiol-terminated PEG chain [163], as illustrated in Fig. 4B. Importantly, they found that this encapsulation system can significantly extend the storage stability of sEVs while maintaining the integrity and biological functions of the vesicles even in extreme storage environments.

To enhance transdermal delivery against skin aging, Li and co-workers developed sEV-based spherical nucleic acid nanomotor (NM-ESNA), with sEV as the core and MMP1-targeting siRNA as the shell [164]. As presented in Fig. 4C, sEV was engineered by siRNA-CHOL to form the ESNA structure. To enhance transdermal delivery, AMC-CHOL (NO) was asymmetrically modified on one side of ESNA, forming NM-ESNA. On light irradiation, gas was released and accumulated on one side, creating a pressure gradient that drove the nanosystem to reach the dermis. This process facilitated the synergistic anti-skin aging effects of sEV and siRNA-SNA via the paracrine effect and gene therapy. NM-ESNA achieved light-controlled deep penetration into mouse skin, effectively reversing skin aging signs effectively.

Additionally, EV-integrated nanocomposites can be also fabricated through the combination of endogenous loading and exogenous modification. In a typical study, Sun and colleagues reported a novel nanodrug-engineered exosome system to achieve a jointly dual-pathway inhibition of cuproptosis [175]. In this system, BMSC-derived exosomes were obtained using gradient centrifugation, followed by engineering with siRNA-ferredoxin 1 (siFDX1). Simultaneously, a hydrophobic copper chelator, Clioquinol (CQ), was encapsulated within a polydopamine-based nanocarrier (PDA@CQ) through self-polymerization of catechol and drug absorption technique. The nanodrug-engineered exosomes, designated as EXO^siFDX1−PDA@CQ^, were subsequently developed by integrating PDA@CQ into exosomes. Upon administration, the PDA shell of EXO^siFDX1−PDA@CQ^ first releases CQ, followed by decomposition of the exosome membrane, which facilitates the release of siFDX1 to downregulate FDX1 expression.

### 3.2 Hydrogels

Hydrogels are highly hydrated crosslinked polymeric materials with a 3D structure, showing great potential in clinical applications [176]. In recent years, hydrogels have received extensive attention in wound healing due to their excellent biochemical and mechanical properties [177]. They can promote O_2_ exchange [178], absorb wound exudate [179,180], maintain a moist environment [181], provide an ideal platform for cell spreading [182], and serve as an effective drug delivery system [183]. Moreover, the combination of hydrogels with EVs has demonstrated considerable potential in skin wound repair. Hydrogels prolong their retention time (avoiding sudden release of EVs), control EV release (achieved by hydrogel crosslinking density and pore size), improve biological stability, prevent premature clearance, and optimize their therapeutic efficacy, as well as easy to transport and store EVs [184]. Hydrogels as EV delivery platforms can be fabricated via either physical or chemical crosslinking. Physical crosslinking mainly relies on reversible non-covalent interactions, including metal ion coordination, hydrogen bonding, hydrophobic interactions, and host-guest supramolecular interaction. However, these crosslinks are generally weak due to their reversible nature. In contrast, chemical crosslinking involves permanent covalent bond formation and offers great mechanical stability. Common chemical crosslinking strategies includes free radical polymerization (e.g., photo-crosslinking) and condensation or addition reactions, such as Schiff-base reaction, thiol-ene click chemistry, N-hydroxysuccinimide ester coupling, and crosslinking with glutaraldehyde, genipin, or acetic acid. In the following subsections, we will focus on introducing several common crosslinking strategies utilized for preparing hydrogels that carry EVs.

#### 3.2.1 Photo-crosslinking

**Fig. 5:**
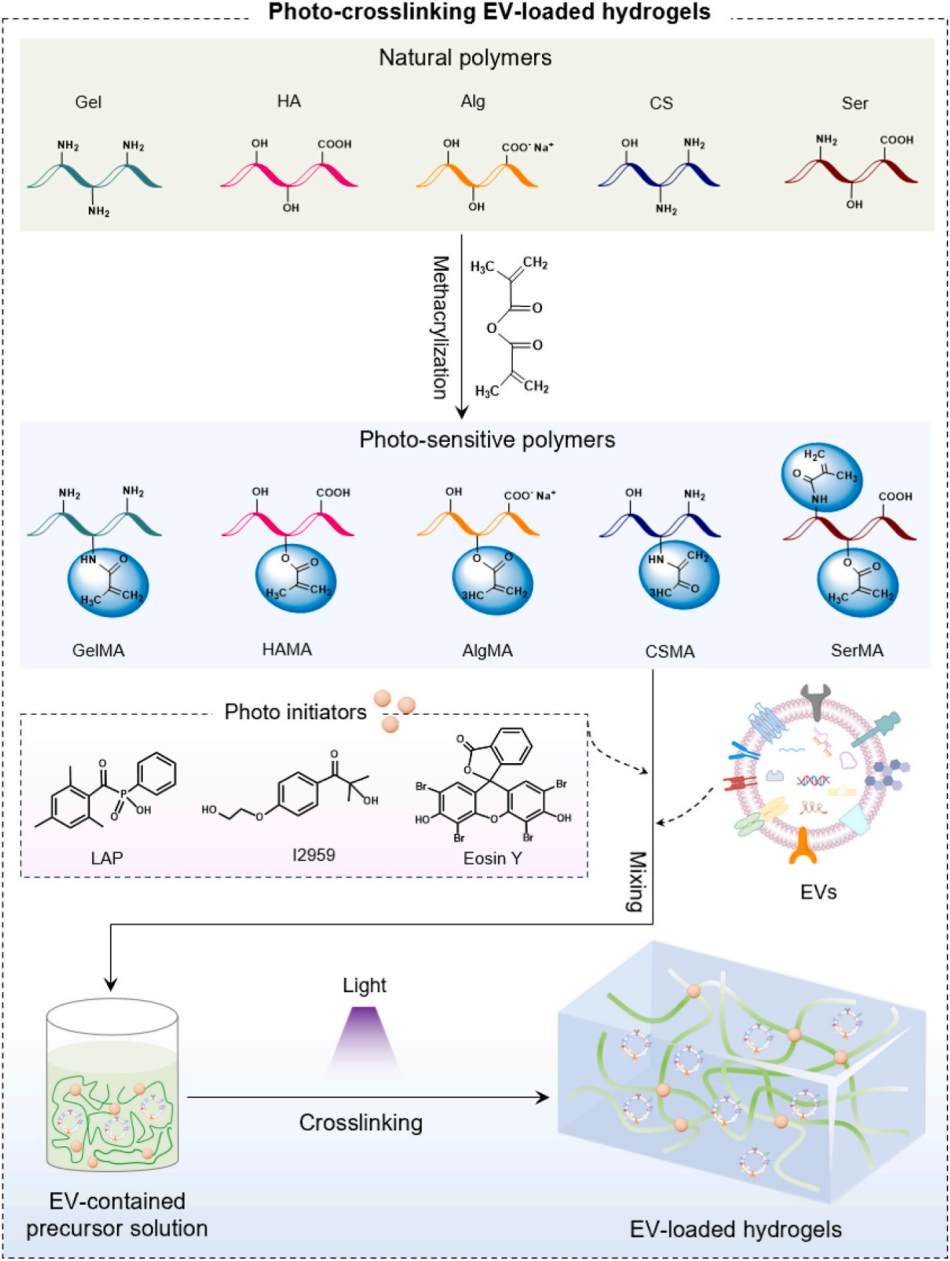
Schematic representation of the synthesis procedures for frequently utilized photo-sensitive polymers (GelMA, HAMA, AlgSA, CSMA, SerMA) and fabrication methodology of photo-crosslinked EV-loaded hydrogels achieved by crosslinking the photo-sensitive polymer precursor solutions upon light irradiation in presence of photo initiators (such as LAP, I2959, Eosin Y).

Photo-crosslinked hydrogels stand out as particularly promising biomaterial platforms for skin wound therapies. Their unique photosensitivity, injectability and spraying property enable rapid light-triggered gelation, transitioning from liquid to gel state within seconds. This phase-change capability enables precise conformability to complex wound morphologies while maintaining therapeutic coverage integrity, criticial for tissue regeneration. The photo-sensitive polymers, including methacrylic gelatin (GelMA) [[185], [186], [187], [188]], methacrylic hyaluronic acid (HAMA) [189], methacrylic alginate (AlgMA) [190], methacrylic chitosan (CSMA) [191,192], methacrylic sericin (SerMA) [193] and methacrylated konjac glucomannan (KGMMA) [194] can be prepared by methacrylizing the natural polymers, gelatin (Gel), hyaluronic acid (HA), sodium alginate (Alg), chitosan (CS), sericin (Ser) and konjac glucomannan (KGM), with methacrylic anhydride (MA), respectively. Moreover, the photosensitivity of these polymers stems from the grafting of methacrylate groups, which facilitates the formaion of a stable covalently bonded polymer network [195]. As shown in Fig. 5, the amide groups of MA can react with -NH_2_ or -OH in Gel, HA, Alg, CS, and Ser, to form methacrylate groups. The degree of methacryloyl substitution in photo-sensitive polymers can be adjusted by changing the MA amount in the reaction systems [196]. Furthermore, by mixing EVs with photosensitive polymers, the photo-crosslinked EV-loaded hydrogels can be fabricated upon light irradiation in the presence of photo-initiators (e.g., LAP [197], I2959 [198], Eosin Y [199]).

Overall, the photo-crosslinking method has several advantages, including rapid gelation (completed within seconds to minutes), adjustable mechanical properties and pore size, as well as adaptability to irregular wounds. However, the ultraviolet light and free radicals generated during polymerization process may damage EV cargos (e.g., mRNA, miRNA, proteins). Additionally, the photo-initiators may be cytotoxic.

#### 3.2.2 Schiff-base crosslinking

**Fig. 6:**
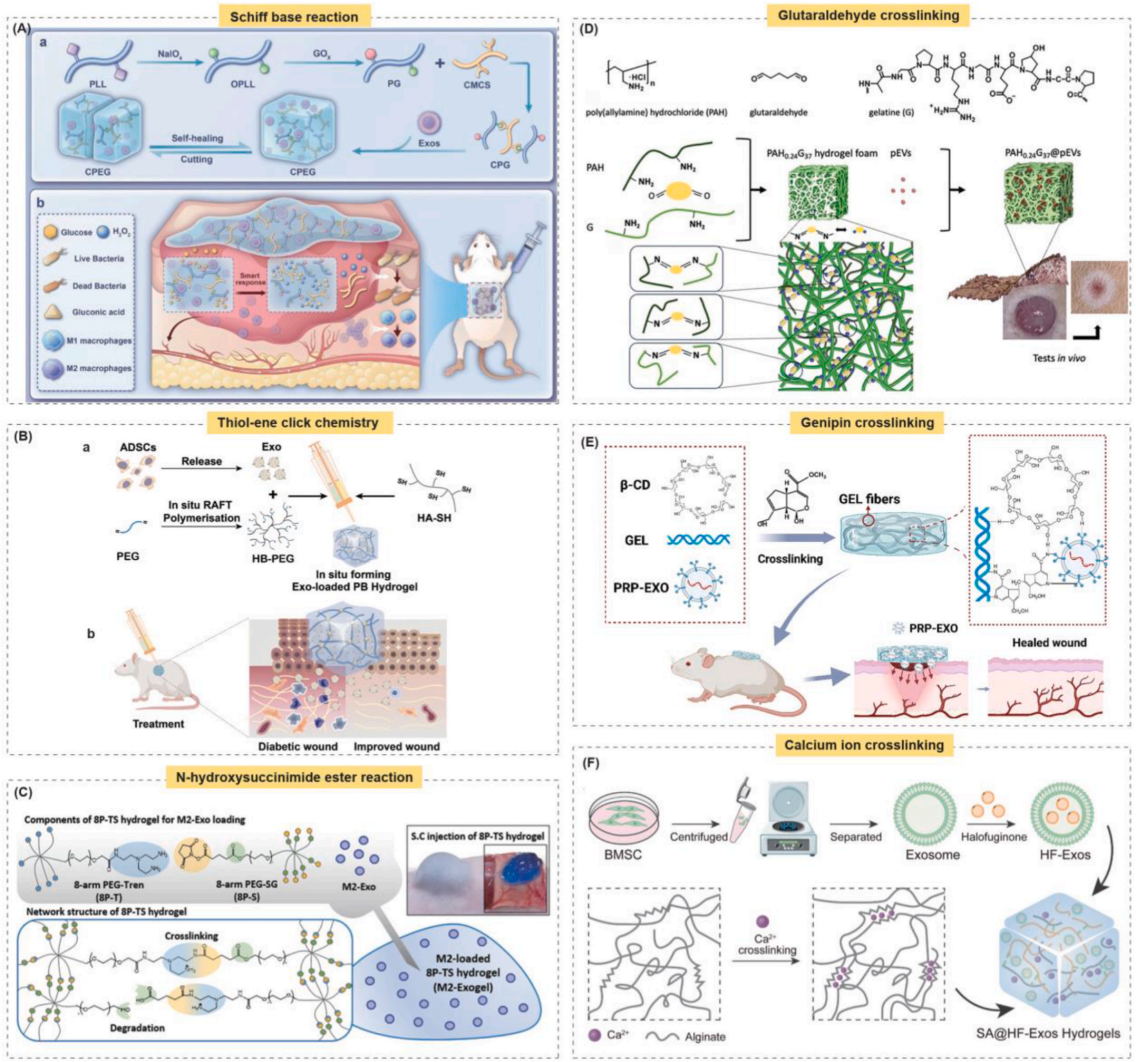
**Commonly used crosslinking methods for the preparation of hydrogels carrying EVs**. Representative examples of the fabrication processes of EV-loaded hydrogels through various methods: (A) Schiff-base reaction. Reproduced with permission [207]. Copyright 2024, Wiley-VCH. (B) Thiol-ene click chemistry reaction. Reproduced with permission [208]. Copyright 2024, Wiley-VCH. (C) NHS ester reaction. Reproduced with permission [209]. Copyright 2022, Wiley-VCH. (D) GTA crosslinking. Reproduced with permission [210]. Copyright 2024, Wiley-VCH. (E) GNP crosslinking. Reproduced with permission [211]. Copyright 2023, Elsevier B.V. (F) Ca^2+^ crosslinking. Reproduced with permission [212]. Copyright 2025, Elsevier B.V.

Schiff base, first proposed by Hugo Schiff in 1864, is formed by a reaction between a primary amine and a carbonyl compound [200]. Based on Schiff base linkages, hydrogel can be developed with the formation of covalent imine bonds (-CH=N-) from the condensation of aldehyde groups (-CHO) and amino groups (-NH_2_) within polymer chains, without requiring additional reagents or external stimuli [201,202]. Commonly used aldehyde-containing polymers include oxidized konjac glucomannan (OKGM), oxidized dextran (OD), oxidized starch (OST), oxidized hyaluronic acid (OHA). Typical amine-containing polymers comprise carboxymethyl chitosan (CMC), and hydroxybutyl chitosan (HBC). The Schiff-base reaction between -CHO and -NH_2_ groups on the polymer backbones endowed the fabricated hydrogels with self-healing ability. Nevertheless, achieving autonomous self-healing while maintaining high crack resistance remains a fundamental trade-off issue in the development of soft materials [203]. Through the dynamic Schiff-base crosslinking, the EV-loaded hydrogels not only retain the self-healing property but also exhibits good injectability and sustained release performance for encapsulated EVs [204,205]. For instance, Zhao and co-workers developed an ARC hydrogel incorporating MSC-derived exosomes for multimodal therapy of diabetic foot ulcers [206]. The hydrogel was prepared through Schiff-base crosslinking between the -CHO groups of aldehyde functionalized hyaluronic acid (AHA) modified with L-Arg (AR) and the primary amino groups of CMCS. The resulting Exo@ARC hydrogel exhibited NO sustained-release effects. In another study, Yang and co-workers developed an exosome-loaded GOx-modified hydrogel for promoting diabetic wound repair [207]. In this system, glucose oxidase (GOx) was grafted onto oxidized pullulanpolysaccharides (OPLL), followed by reacting with carboxymethyl chitosan (CMCS) to form a CPG hydrogel, as illustrated in Fig. 6A. The reaction mechanism was based on a reversible Schiff base covalent crosslinking reaction between -CHO groups on OPLL and -NH_2_ groups on CMCS. To further enhance biological functions of the hydrogel, ADMSC-derived exosomes (Exos) were introduced into CPG hydrogel, resulting in CPEG hydrogel formation. Owing to Schiff base bonds formation, CPEG hydrogel showed self-healing property. Moreover, this hydrogel exhibited dual-responsive characteristics (glucose-responsive and pH-responsive) for sustained exosome release. Additionally, this hydrogel showed good antibacterial properties attributed to the introduction of CMCS. All these properties contrubuted to the healing process of wounds.

Overall, the Schiff base reaction offers several advantages for the fabrication of hydrogels carrying EVs, including mild reaction conditions, rapid gelation, and catalyst-free process, which collectively preserve the bioactivity of EVs. Moreover, the resulting dynamic and reversible Schiff-base bonds endow the hydrogels with injectability, self-healing capability, and pH-responsive EV release properties, making them particularly suitable for irregular wound applications. However, this reaction also presents several limitations. For instance, it is highly sensitive to water content and pH, and the crosslinking density is difficult to precisely control. Additionally, aldehyde-based crosslinkers may exert slight cytotoxicity when used at elevated concentrations.

#### 3.2.3 Thiol-ene click chemistry

Click chemistry, first proposed by Sharpless in 2001, was recognized with the Nobel Prize in Chemistry in 2022 [213]. Among its various reaction types, the thiol-ene click reaction is a free radical-mediated addition reaction, involving the free radiacal-trapping capacity of thiol groups (-SH) that subsequently react with alkene moieties (-C=C-) to form stable carbon-sulfur (C-S) covalent bonds. This reaction boasts multiple distinctadvantages: (i) mild reaction conditions that obviate the need for water and oxygen exclusion, (ii) high efficiency and rapid kinetics characterized by fast reaction rates, high product yields, and minimal by-product formation, and (iii) broad substrate diversity, as nearly all thiol-containing compounds and a wide range of alkenes are compatible as reaction substrates [214]. Moreover, due to the mild conditions of thiol-ene click chemistry reaction, the structural integrity of EVs and their inherent biological activity are maintained throughout the reaction process. Collectively, the thiol-ene click chemistry strategy has emerged as an efficient, mild, and highly promising approach for the encapsulation and functionalization of EVs into hydrogels [[215], [216], [217]]. For instance, Wang and co-workers developed a hypoxia-induced exosome-laden injectable hydrogel for enhancing diabetic wound healing [208], as illustrated in Fig. 6B. The hydrogel was fabricated by mixing the hyperbranched PEG (HB-PEG) with thiol-modified hyaluronic acid (HA-SH) to form a hydrogel precursor solution, followed by loading exosomes derived from hypoxia-preconditioned ADSCs. Upon mixing, a thiol-ene Michael addition reaction rapidly occurred between -C=C- groups on HB-PEG and -SH groups on HA-SH, yielding an in-situ crosslinked injectable hydrogel. The hydrogel achieved continuous release of exosomes over fifteen days. This exosome-loaded hydrogel system offers a promising strategy for efficient exosome delivery in wound healing and holds potential for broader applications in regenerative medicine.

Overall, the thiol-ene click crosslinking reaction offers several advantages for the preparation of hydrogels carrying EVs, primarily including high crosslinking efficiency, ultra-fast reaction rates (often complete within minutes), and the absence of toxic by-products. However, this method also presents several limitations, such as requiring expensive raw materials (such as thiolated polymer precursors), strict reaction conditions, and lack of self-healing property (irreversible crosslinking).

#### 3.2.4 N-hydroxysuccinimide ester reaction

N-hydroxysuccinimide (NHS) ester reaction is a common type of reaction in organic chemistry, and it has important application value in drug synthesis and biochemical research for drug labeling, drug release and protein modification [218]. Its mechanism involves transesterification between an NHS ester and primary amine groups to form an activated ester intermediate, which subsequently undergoes a nucleophilic addition reaction with an amino-containing compound, ultimately yielding an amide product [[219], [220], [221]]. The NHS ester reaction offers several advantages: (i) mild reaction conditions, rapid reaction rate and high yields; (ii) good selectivity which can selectively react with compounds with amino groups, without intefering with other functional groups. This reaction has been successfully employed for the fabrication of EV-loaded hydrogels [222,223]. In a typical example, Kwak and co-workers fabricated a hydrolytically degradable hydrogel that enabled sustained exosome-guided macrophage polarization for wound healing [209], as illustrated in Fig. 6C. In this system, the hydrolytically degradable hydrogel containing 8-arm PEG (8P) was designed with two chemical groups, tris(2-aminoethyl)amine (Tren) and N-succinimidyl glutarate (SG). The 8-arm PEG Tren-SG (8P-TS) hydrogel carrying M2-Exos (M2-Exogels) was prepared by mixing freeze-dried 8-arm PEG Tren (8P-T) and 8-arm PEG-SG (8P-S) with M2-Exo solution through a crosslinking reaction between the amine groups on 8P-T and NHS ester groups on 8P-S. Importantly, the biodegradability of the M2-Exogels was conferred by ester groups within the SG linker. By altering the composition of the hydrogel, the release period of M2-Exos from Exogels could be adjusted. The release of M2-Exos was closely related to the degradation of the hydrogel and generally reached complete release before the complete degradation of the hydrogel. All Exogels exhibited an initial burst release, followed by a sustained slow-release phase, which accelerated shortly before the complete dissociation of the hydrogel.

Overall, NHS ester reaction possesses several advantages, including the ability to rapidly from amide bonds under mild aqueous conditions, the absence of toxic by-products, high mechanical strength and stability, good biocompatibility, and suitability for the preparation of protein-based hydrogels. However, this reaction also faces some challenges, including: (i) NHS esters are prone to hydrolysis; (ii) they react indiscriminately with the amino groups on the surface of EVs; (iii) high cost of raw materials.

#### 3.2.5 Glutaraldehyde crosslinking

Glutaraldehyde (GTA), with the chemical formula C_5_H_8_O_2_, is a dialdehyde compound containing two aldehyde groups (-CHO). It can react with -NH_2_ groups to form an imine bond (-C=N-) [224]. This crosslinkling reaction thereby significantly enhances the mechanical properties, thermal properties and chemical resistance of the material [225,226]. The chemical crosslinking between GTA and other compounds containing -NH_2_ groups or -OH groups has emerged as a versatile strategy for fabricating EV-loaded hydrogels. Such hydrogels are typically constructed from nature and synthetic polymers, including proteins and amino-rich polysaccharides [227]. In a typical study, Back and co-workers developed a gelatine (GEL) hydrogel loaded with platelet-derived EVs, naming it PAH-G@pEVs [210], as illustrated in Fig. 6D. Briefly, PAH-G@pEVs was constructed by crosslinking GEL with poly(allylamine hydrochloride) (PAH) using GTA, followed by freezing, washing, and drying at room temperature, and then completely adsorbing pEVs onto the hydrogel. PAH-G@pEVs exhibited a burst release of pEVs during the early stage and then increased over time. This PAH-G@pEVs hydrogel demonstrates great potential in improving wound healing.

Overall, GTA crosslinking offers several advantages, including high crosslinking efficiency, low cost, high mechanical strength, and straightforward scalability. However, GTA exhibits significant cytotoxic and causes tissue irritation, which may severely compromise the bioactivity of EVs. Furthermore, GTA-crosslinked hydrogels typically suffer from poor biodegradability, limiting their long-term applicability in biomedical settings.

#### 3.2.6 Genipin crosslinking

Genipin (GNP), with the chemical formula C_11_H_14_O_5_, is an aglycone derived from geniposide (an iridoid glycoside) and isolated from the fruits of Gardenia jasminoides Ellis [228,229]. As a naturally occurring crosslinking agent, GNP has garnered significant attention in the fields of biomaterials. The cytotoxicity of GNP is 10-fold lower than that of GTA [230]. Functionally, GNP readily reacts with amino-containing biopolymers (such as GEL, chitosan, silk fibroin, and so on) [231], to form both intra- and inter-molecularly crosslinked products with a robust and stable crosslinking network [232]. The crosslinking mechanism of GNP in primary amine-rich hydrogels involves the formation of heterocyclic compounds, which covalently conjugate with the biomacromolecular chains of the hydrogel matrix. For instance, Shu and co-workers developed a fiber-reinforced GEL/β-cyclodextrin (β-CD) hydrogel functionalized with PRP-derived exosomes (PRP-EXOs) for treating diabetic wounds [211], as illustrated in Fig. 6E. In this system, GEL/β-CD/PRP-EXOs hydrogel was fabricated with GNP as a crosslinking agent, which reacted with -NH_2_ groups in GEL to form covalent bonds, where β-CD interacted with GEL and GNP through hydrogen bonding. Moreover, PRP-EXOs were encapsulated within the hydrogel through GNP-mediated covalent crosskining. In vitro release studies demonstrated a sustained release of PRP-EXOs. Functionality, the encapsulation of PRP-EXOs into GEL/β-CD hydrogel significantly enhanced autophagy and inhibited apoptosis in HUVECs and HSFs, thereby supporting diabetic wound repair.

Overall, GNP crosslinking possesses several advantages, primarily including low cytotoxicity, high biocompatibility, good biodegradability, and inherent anti-inflammatory properties. However, the purity/batch consistency of GNP is difficult to control; and when used at high doses, the potential risk of liver toxicity needs to be fully considered.

#### 3.2.7 Metal ion crosslinking

Polymers with negatively-charged functional groups, such as carboxyl groups (-COOH) and phosphate groups (-PO_3_), can be crosslinked via ionic bonds with multivalent metal ions, leading to the formation of stable 3D hydrogel networks. This metal ion-mediated crosslinking strategy has been widely utilized to enhance mechanical strength of polymeric hydrogels [233]. By coordinating with functional groups along the polymer chains, metal ions increase the crosslinking density, thereby improving mechanical properties such as stiffness, toughness, and elastic recovery. Moreover, metal-carbonate coordination offers a versatile platform for biomineralization and pH-triggered therapeutic delivery [234]. Currently, metal ions such as Cu^2+^ and Ca^2+^ are the commonly used crosslinkers to fabricate EV-contained hydrogels.

Sodium alginate (SA) hydrogels can be readily constructed via facile noncovalent crosslinking by multivalent metal ions (e.g., Ca^2+^, Fe^2+^, Fe^3+^) [235,236]. Among these, Ca^2+^ has been extensively utilized to fabricate EV-laden SA-based hydrogels, primarily through coordination interactions between the multivalent ion Ca^2+^ and the carboxyl groups of SA chains. Importantly, Ca^2+^-crosslinked SA-based hydrogel can effectively reduce the release rate of EVs, thereby achieving a more controllable and sustained release behavior [237]. For instance, Gao and co-workers developed an ultrasound (US)-responsive hydrogel integrated with engineered exosomes for the delivery of halfuginone (HF) [212], as illustrated in Fig. 6F. The hydrogel was fabricated by mixing HF-loaded exosomes with a SA slution, followed by ionic crosslinking with Ca^2+^. The release rate of HF exhibited a time-dependent increase, which could be further enhanced upon US exposure. This phenomenon was primarily due to the disruption of ionic crosslinking between SA and Ca^2+^ induced by US, leading to accelerated hydrogel degradation and consequently enabling more efficient drug release. Zhang and co-workers developed a sprayable alginate (SA) hydrogel dressing, designated as CP/EXO/SA [238]. The hydrogel was fabricated by injecting a gel precursor solution onto the wound surface, which comprised SA, BMSC-derived exosomes (EXO), calcium peroxide (CP)-laden poly(L-lactic acid) (PLLA) microspheres, and catalase. Subsequently, calcium chloride was sprayed onto the SA surface to induce rapid crosslinking, to form in-situ adaptable CP/EXO/SA hydrogel. This hydrogel dressing effectively alleviated cellular hypoxia in chronic diabetic wounds, accelerated wound closure, enhanced angiogenesis, and promoted the M2 polarization of macrophages. These fingdings highlight that CP/EXO/SA, with multifunctional therapeutic effects and ease of use, is an ideal dressing for clinical transformation and wide application.

In an another study, through Cu^2+^ crosslinking, Zhou and co-workers fabricated an extracellular matrix hydrogel, designated as exo^FGF2^@ECM/Cu^2+^, incorporating fibroblast growth factor 2-engineered exosomes (exo^FGF2^) [239]. The hydrogel was fabricated by introducing exo^FGF2^ and Cu^2+^ into decellularized extracellular matrix (dECM), where Cu^2+^-mediated crosslinking via ligand bonds resulted in a stable hydrogel network. The exo^FGF2^@ECM/Cu^2+^ hydrogel exhibited favorable biodegradability, biocompatibility, and hemostatic capability. Functionally, it demonstrated potent efficacy in wound healing by facilitating angiogenesis, collagen deposition, and re-epithelialization. Moreover, this hydrogel effectively inhibited bacterial growth for preventing wound infections. These findings highlight the promising potential of exo^FGF2^@ECM/Cu^2+^ in advancing wound healing across diverse clinical applications.

Overall, metal ion crosslinking has several advantages, including ultra-mild reaction conditions (aqueous, room temperature) and reversible reaction processes (metal ions can be released through clelation reactions). However, the coordination effect is weak, resulting in poor stability and low EV loading. Additionally, excessive metal ions may also cause potential cytotoxicity.

#### 3.2.8 Multiple crosslinking

Multiple crosslinking strategies employing various crosslinking strategies have been also utilized to prepare EV-loaded biomaterials for wound treatment, such as Ca^2+^/Schiff-base dual-crosslinking [240], Ca^2+^/photo irradiation dual-crosslinking [241], Ca^2+^/GNP dual-crosslinking [242], enzyme/Schiff-base dual crosslinking [243], Schiff-base/photo irradiation dual-crosslinking [244]. For example, Shen and co-workers developed a sprayable hydrogel (OSA-EPL-Exo) for wound repair and health monitoring [240]. Specifically, the hydrogel was prepared by mixing two solutions: one containing low-oxidized sodium alginate (OSA) and exosomes, another containing EPL with calcium chloride (CaCl_2_). Each solution was separately loaded into different spray bottles and applied simultaneously. This ensured that the solutions would only mix when in contact with the wound surface, allowing for immediate gelation at the wound site and ultimately forming a hydrogel. The hydrogel formation involved two crosslinking mechanisms: (i) -CHO groups in OSA reacted with -NH_2_ groups in EPL to form Schiff-base bonds, and (ii) unoxidized -COOH groups in OSA crosslinked by Ca^2+^. This dual-crosslinking process formed a robust hydrogel dressing on the wound surface, resulting in a physical barrier for the suppression of external infection. In addition, OSA-EPL-Exo promoted wound healing by regulating immune responses, promoting angiogenesis and collagen deposition. Impressively, this hydrogel can also monitor heartbeat and muscle movement, suggesting its health monitoring functions. Overall, multiple crosslinking integrates advantages of single crosslinking strategies. However, this method also faces some challenges, including: (i) complex preparation and characterization process; (ii) potential incompatibility between crosslinking chemicals; (iii) batch-to-batch variations; (iv) challenging for regulatory approval.

### 3.3 Microneedle patches

Microneedle patches (MNPs) consist of a microneedle base and an array of needle tips, and have great potential in transdermal drug delivery [245]. The Characteristic of MNPs is that it can perforate the stratum corneum through minimally invasive microchannels, effectively bypass the barrier function of skin, and enable the therapeutic agents to be targeted and delivered to deeper skin layers [246]. This structure facilitates the encapsulation of a variety of bioactive cargoes within the microneedle matrix, mainly including small molecule drugs [[247], [248], [249]], growth factors [[250], [251], [252]], and EVs [[253], [254], [255]]. Upon application, these needle-like structures penetrate wound sites, achieving high local concentration and optimized therapeutic effects. The unique advantages of EV-loaded MNPs, especially their minimally invasive drug delivery, prolonged drug retention time, and continuous release characteristics, make them as a pioneering platform for EV-mediated wound treatment [256,257]. Currently, the fabrication of EV-loaded MNPs primarily relies on three strategies, broadly categorized into: (i) direct micro-molding method, (ii) combination of micro-molding and photo-crosslinking, and (iii) combination of micro-molding, photo-crosslinking and electrostatic interaction. In the subsections, we will discuss these three approaches.

#### 3.3.1 Direct micro-molding method

**Fig. 7:**
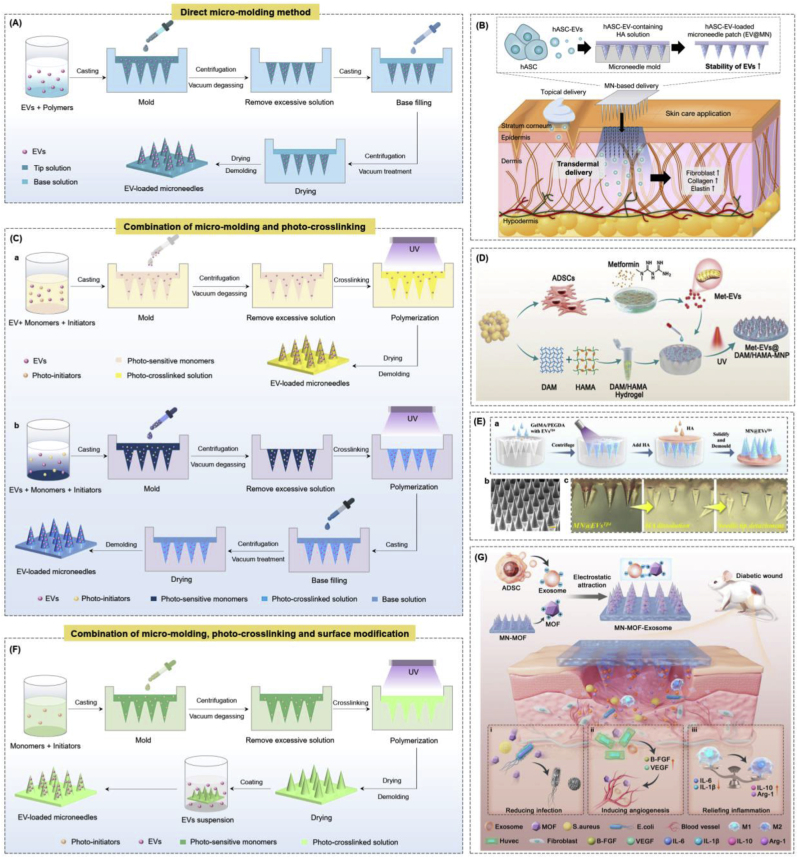
**Representative methods for the fabrication of EV-loaded microneedle patches**. Schematic illustration of three primary strategies for preparing EV-loaded MNPs: (A) direct micro-molding method, (C) combination of micro-molding and photo-crosslinking, and (F) combination of micro-molding, photo-crosslinking and surface modification. (B) Schematic illustration of hASC-EV-loaded microneedle patch. Reproduced with permission [258]. Copyright 2022, Elsevier Ltd. (D) Schematic illustration of Met-EVs@ADM/HAMA-MNP fabrication. Reproduced with permission [259]. Copyright 2024, American Chemical Society. (E) (a) Schematic diagram illustrating the fabrication of MN@EVs^Tβ4^, (b) SEM image of MN@EVs^Tβ4^, and (c) images of the complete process before and after the tip detachment in MN@EVs^Tβ4^. Reproduced with permission [260]. Copyright 2025, Wiley-VCH. (G) Schematic showing exosomes autonomously assemble on the surface of MOF-loaded microneedles through electrostatic attraction. Reproduced with permission [261]. Copyright 2025, Elsevier Ltd.

Direct micro-molding tmethod can be used to prepare EV-loaded MNPs. As illustrated in Fig. 7A, a homogenous mixed solution containing EVs and biocompatible polymers (e.g., HA, PVA, PLGA, PVP, CMC, SF, Gel, Col) is filled into a mold, followed by centrifugating and vacuum degassing to ensure full filling. Subsequently, a base solution is introduced to form the patch backing layer, and finally cured by drying and demolding, resulting in the formation of EV-loaded MNPs. As a typical example, Bui and co-workers developed HA dissolvable microneedles loaded with EVs derived from human adipose stem cells (hASC-EVs) [258]. Briefly, as illustrated in Fig. 7B, an aqueous solution containing HA and hASC-EVs was cast into a mold under vacuum, followed by the removal of the residual solution. Threafter, a separate HA solution was poured onto the surface of mold and dried under air flow within a clean bench. Finally, the resulting EV@MN were separated from the silicone mold. In another study, Lim and co-workers developed dissolvable microneedles (MNs) to develiver and preserve EVs derived from natural killer cells (NK-EVs) [262]. Specifically, MNs were prepared by pipetting NK-EVs onto a PDMS mold to ensure that NK-EVs were concentrated at the needle tip. The mold was then placed under a vacuum state to ensure that the EVs would settle into the needle tips and allow all loaded EVs to be released consistently and effictively. The mixture of HA and trehalose was then added into the mold and vacuumed. This step was repeated with an additional layer of the HA-trehalose solution to make up the MN backing, followed by another vacuum treatment. Finally, the mold was dried overnight to complete the formation of MN. They found that MN can stabilize NK-EVs proteins and allow NK-EVs to be stored and used for a long time.

#### 3.3.2 Combination of micro-molding and photo-crosslinking

Beside the direct micro-molding method, a micro-molding technique coupled with photopolymerization can be also utilized to prepare EV-loaded MNPs, as illustrated in Fig. 7C. A precursor solution, containing EVs, photosensitive monomers (e.g., GelMA, HAMA, CSMA) and photo initiators (e.g., LAP, I2959, Eosin Y) is filled into a mold, followed by centrifugating and vacuum degassing. Subsequently, light irradiation triggers in-situ crosslinking of the precursor solution to immobilize EVs within the polymer networks, and finally curing through drying and demolding.

For instance, Yao and co-workers developed hydrogel MNPs containing stem cell mitochondria-enriched microvesicles for boosting the healing of chronic wounds [259]. Briefly, as illustrated in Fig. 7D, HAMA pregel was prepared by mixing HAMA and LAP, followed by mixing with DAM. Subsequently, Met-EVs were mixed with DAM/HAMA before the addition of them to a mold, followed by centrifugation, removing bubbles, and crosslinking under visible light, forming Met-EVs@DAM/HAMA-MNPs. In another study, Ding and co-workers developed a novel separable MNPs for EVs delivery to facilitate diabetic wound healing [260]. Specifically, as illustrated in Fig. 7E(a), Tβ4-modified adipose-derived stem cell EVs (EVs^Tβ4^) were mixed with a solution contaning GelMA and PEGDA, and then poured into a PDMS mold. Upon UV light irradiation, the biodegradable microneedle tips were obtained. Subsequently, HA solution was filled into base layer and dried. This process ultimately resulted in separable MN@EVs^Tβ4^ MNPs with GelMA/PEGDA as microneedle tips and HA as backing layer. The microneedle tips were neatly arranged on the HA substrate, as presented in Fig. 7E(b). To assess whether the microneedle tips were detached from the base, MN@EVs^Tβ4^ was immersed in PBS. As presented in Fig. 7E(c), the base layer dissolved after absorbing PBS, promoting the rapid separation of the microneedle tips from the patch. Mechanically, MN@EVs^Tβ4^ rescued cell senescence and improved cell function through the PTEN/PI3K/AKT signaling pathway, thereby promoting wound healing. This engineered EV-based MNP is expected to be applied in clinical wound management.

In another study, Zeng and co-workers developed a bilayer microneedle-based wound dressing with mild photothermal therapy to promote the healing of diabetic wounds [263]. This system was designed by encapsulating M2 macrophage-derived exosomes (MEs) within the needle tips while embedding PDA NPs in the backing layer, to synergistically inhibit inflammatory responses and facilitate angiogenesis. Briefly, MEs-HAMA precursor solution was prepared by homogenously dispersing MEs in a HAMA solution along with a photo-initiator of LAP, while PDA-PVA solution was prepared by incorporating PDA NPs into a PVA solution. Subsequently, MEs-HAMA pecursor solution was filled into a PDMS mold, followed with centrifugation, vacuum degassing and drying. Afterwards, MEs-HAMA was photo-crosslinked upon exposure to UV. Finally, PDA-PVA solution was applied to coat the needle tips, followed by drying and demolding to obtain the final bilayer microneedle patch (MEs@PMN). In vitro release experiments demonstrated that MEs were rapidly released in the initial phase, followed by stabilization.

#### 3.3.3 Combination of micro-molding, photo-crosslinking and surface modification

Beside the abovementioned methods, a micro-molding technique coupled with photopolymerization and surface modification can be also utilized to prepare EV-loaded MNPs, as illustrated in Fig. 7F. A precursor solution, containing photosensitive monomers and photo initiators is filled into a mold, followed by centrifugating and vacuum degassing. Subsequently, light irradiation triggers in-situ crosslinking of precursor solution, followed by curing through drying and demolding. Then, the obtained MNPs were immersed into EV suspension, resulting in the formation of EV-loaded MNPs.

In a typical study, through the electrostatic interaction between exosomes and MOFs, Xiang and co-workers developed a MOF microneedle patch loaded with exosomes for improving diabetic wound healing [261]. They prepared ZIF-8-incorporated GelMA/PEGDA MN through combining micro-molding and photo-crosslinking. Subsequently, the exosome suspension was added to the MN and incubated to enable the exosomes to be loaded into the MNs. The exosomes showed a negative zeta potential, while ZIF-8 displayed a positive zeta potential. This significant charge difference plays a crucial role in regulating the electrostatic interaction between the exosome membranes and the surface of ZIF-8, as illustrated in Fig. 7G. The wound healing mechanism of the MN system includes (i) disinfection: the MNPs carrying MOFs can inhibit the growth of microbial pathogens on the wound surface, (ii) angiogenesis: MOFs and exosomes are transported to the subcutaneous layer through the needle tip, where their synergistic effect induces angiogenesis, (iii) anti-inflammation: inhibits chronic wound-related inflammation by promoting macrophage polarization. This system combines the efficient electrostatic self-adsorption of exosomes onto the MOF within a MN structure, enabling the therapeutic agents to be delivered directly to the wound site in a precise, minimally invasive, and effective manner.

In another study, by combining micro-molding, photo-crosslinking, freeze-drying circulation and surface coating techniques, Xu and co-workers prepared a novel double-layer HAMA/PVA MNP modified with young fibroblast-derived exosomes (Y-EXOs) for antibacterial, immunotherapy, and skin regeneration [264]. Briefly, Zn-incorporated HAMA/PVA MNP was fabricated through micro-molding and photo-crosslinking process, with HAMA as the microneedle tips and PVA as the substrate. Subsequently, Y-EXOs were coated onto the HAMA/PVA MNP via spraying and freeze-drying cycles, resulting in Y-EXOs@HAMA/PVA MNP. This MNP demonstrated superior efficacy in promoting the delayed healing of aged skin wounds, characterized by increased activation of fibroblasts and increased collagen deposition.

The majority of reported MNP systems exhibit conical and smooth surfaces, which leads to weak adhesion and often necessitate additional suture fixation. Moreover, the non-deformable MN caps currently present significant limitations for joint wound treatment. To address these limitations, Zhou and co-workers drew inspiration by the crab claw, shark microgroove structure, and traditional Chinese paper-cutting techniques, to develop double-bionic deformable DNA hydrogel MNs incorporated with EVs for promoting diabetic ulcer repair [265]. To ensure that EVs function effectively in the hypoxic environment of chronic wounds, hair follicle stem cells (HFSCs) were cultured in hypoxic conditions to obtain EVs (H-EVs-hypoxia). The polypeptide-DNA hydrogel MNs (P-DNA gel MNs) were prepared by injecting the mixture of polypeptide or catechol-polypeptide DNA segments, along with other DNA segments and EVs, into a 3D-printed mold. The formation of these hydrogels was facilitated by DNA base pairing, covalent bond crosslinking, and physical entanglement. Specifically, the interaction involving Watson-Crick, Hoogsteen, and dense hydrogen bonds played a crucial role in the formation of P-DNA gel MNs. The double-bionic structures endowed the MNs with crab claw-like, flat, inclined, and shark microgroove structures, which enhanced stability and grip strength and reduced insertion resistance into the skin. In vivo, treatment with P-DNA gel MNs/H-EVs-hypoxia showed obvious healing and achieved a high wound closure rate.

### 3.4 Electrospun fibrous membranes

Electrospinning is an economically viable and technically straightforward method that can utilize a diver array of materials, including polymers and ceramics, to produce nanofibers with a fiber structure analogous to the natural ECM, thereby demonstrating significant potential in skin wound repair [[266], [267], [268], [269]]. Currently, two primary approaches exist for fabricating EV-loaded electrospun membranes: (i) one-step electrospinning like single electrospinning or coaxial electrospinning; (ii) post-modification, such as polyethyleneimine (PEI) assembly, metal-organic framework (MOF)-based immobilization, EDC/NHS amidation reaction, and PDA-mediated bio-interface modification.

#### 3.4.1 One-step electrospinning

**Fig. 8:**
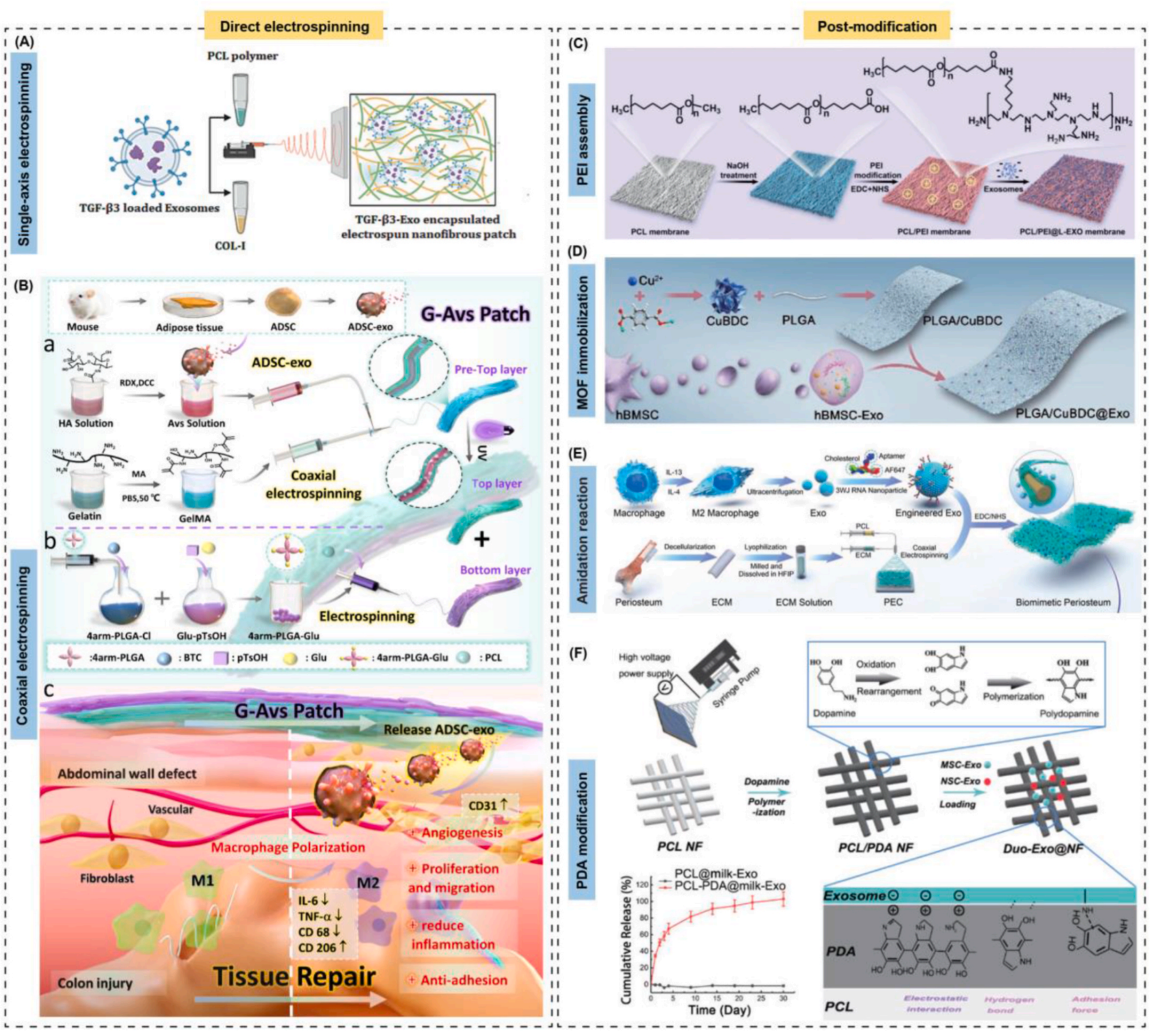
**Representation methods for the fabrication of EV-loaded electrospun membranes**. (A) Schematic illustration of TGF-β3-Exo@NFs fabrication. Reproduced with permission [270]. Copyright 2023, Elsevier Inc. (B) Schematic illustration of the fabrication of G-Avs patch for modulating inflammation and promoting tissue regeneration. Reproduced with permission [271]. Copyright 2025, American Chemical Society. (C) Schematic diagram of PCL/PEI@L-Exo membranes fabrication. Reproduced with permission [272]. Copyright 2025, Elsevier B.V. (D) Schematic illustration of PLGA/CuBDC@Exo scaffold preparation. Reproduced with permission [273]. Copyright 2022, Elsevier B.V. (E) Schematic illustration of preparation process of a bioactive and biomimetic periosteum constructed by combining the electrospun membrane with engineered Exos. Reproduced with permission [274]. Copyright 2025, Elsevier B.V. (F) Schematic illustration of the preparation process for Duo-Exo@NF, including the surface loading mechanisms and cumulative release profile of Milk-Exo from PCL vs. PCL-PDA nanofibers. Reproduced with permission [275]. Copyright 2024, American Chemical Society.

Through direct electrospinning of EV-contained spinning solutions, Ping and co-workers fabricated a blended nanofibrous patch incorporating TGF-β3 and hUCMSC-derived exosomes [270], as illustrated in Fig. 8A. Specifically, the blended PCL/COL-1 spinning solution was prepared by mixing type I collagen (COL-1) with polycaprolactone (PCL). Subsequently, the isolated exosomes and TGF-β3 were homogenously mixed with PCL/COL-1 spinning solution prior to the electrospinning process.

Through coaxial electrospinning technique, Tao and co-workers developed a Janus patch encapsulated with ADSC-derived exosomes (ADSC-exo) for regulating inflammation and promoting tissue regeneration [271]. As illustrated in Fig. 8B, the top layer of the patch was formed by coaxial electrospinning, serving as an exosome delivery system with a core-shell structure by loading ADSC-exo and HA into GelMA, while the bottom layer comprised a 4arm-PLGA(4aPLGA)-Glu/PCL electrospun membrane, which endowed the patch with a lubricious and antifouling surface while providing strong mechanical support. This patch has the potential for clinical transformation and holds broad applicability in the repair of various tissues and organs. In another study, Xue and co-workers fabricated a PCL-EVs nanofiber scaffold with a core-shell structure for accelerating the repair of large-volume skin defects [276]. The PCL-EVs scaffold was prepared using a coaxial electrospinning device, with PCL as the core solution and a mixture of gelatin and BMSC-EVs as the shell solution. Mechanically, this scaffold promoted angiogenesis by activating endothelial HIF-1α-VEGF/cGMP-PKG signaling axis, coupled with enhanced ECM remodeling.

#### 3.4.2 Post-modification

**PEI assembly**: Based on electrostatic interactions between negatively-charged EVs and positively-charged PEI, EVs can be anchored onto the surface of PEI-modified electrospun membranes [277,278]. For instance, Lv and colleagues constructed an electrospun membrane incorporating lipocalin 2 (LCN2)-enhanced exosomes [272], as illustrated in Fig. 8C. To transport negatively-charged exosomes, PCL membrane underwent PEI modification, yielding a PCL/PEI membrane. Owing to the electrostatic interaction between negatively-charged exosomes and positively-charged PEI, the PCL/PEI membrane possessed substantially higher exosome loading efficiency compared to unmodified PCL. Moreover, the PCL/PEI membrane enabled sustained exosome release over a period of four weeks, whereas the limited exosomes carried by the PCL membrane were nearly completely released within the first 24 h. Similarly, Jin and co-workers fabricated exosome-functionalized heterogeneous nanofibrous scaffolds [279]. Specifically, the heterogeneous PCL fibrous scaffold was fabricated by dissolving PCL in trifluoroethanol with an oriented receiver (OR) and the random PCL layer using a flat-plate receiver. The OR fibrous scaffolds were then immersed in a mixture of sodium hydroxide and ethanol, followed by activation with EDC and NHS. Subsequently, a branched PEI solution was utilized to the reaction system to obtain a PEI-modified PCL scaffold (OR-PEI). Finally, the OR-PEI scaffold was immersed in a suspension containing BMSC-derived exosomes to immobilize the exosome.

**MOF-based immobilization**: Through the electrostatic interaction between negatively-charged EVs and positively-charged MOFs, EVs can be immobilized on the surface of MOF-incorporated electrospun membranes. In a representative study, Xu and co-workers integrated exosomes with MOF-modified multifunctional scaffolds [273]. Briefly, as illustrated in Fig. 8D, the pre-synthesized copper-based MOFs (CuBDC) were incorporated in poly(lactic-co-glycolic) acid (PLGA) spinning solution, followed by electrospinning to form PLGA/CuBDC scaffold. Then, exosomes derived from hBMSCs were tethered to the scaffold via physical embedding and electrostatic interactions. The surface of fabricated PLGA/CuBDC@Exo scaffold became obviously rough, due to the incorporation of exosomes. Compared with PLGA scaffold alone, PLGA/CuBDC facilitated a slower release of exosomes and achieved a controllable release of bioactive Cu^2+^ ions. Similarly, Kang and co-workers developed an exosome-loaded magnesium-organic framework (Mg-GA MOF)-based electrospun fibers [280]. Briefly, the pre-synthesized Mg-GA MOF was blended with a PLGA spinning solution and then electrospun into fibers. The resulting PLGA-Mg-GA/MOF fibers with positive charges enabled the tethering of negatively-charged hADSC-Exos through electrostatic interactions. These findings demonstrate the advantages of MOF-modified electrospun fibers in immobilizing EVs and achieving their continuous and slow release.

**Amidation reaction**: Through an EDC/NHS-catalyzed amidation reaction, Wen and co-workers developed an engineered M2 macrophage-derived exosomes-loaded electrospun membrane [274]. Specifically, as illustrated in Fig. 8E, the conjugation of Apt-NP-Exos to PEC was accomplished via an EDC/NHS-catalyzed amide condensation reaction. The resulting PEC-Apt-NP-Exo exhibited a biphasic release profile: an initial burst release of Apt-NP-Exos within the first three days, followed by a sustained and slower release phase.

**PDA modification**: PDA exhibits strong adhesion properties for biological reagents, thereby enabling the immobilization of EVs on electrospun nanofibers surface. PDA surface modification can readily introduce adhesive functionalities such as catechol groups, hydrogen bonds, and electrostatic interactions, making PDA-modified nanofibers an ideal tool for functional exosome delivery. For instance, Li and co-workers employed a PDA-functionalized layer on electrospun PCL nanofibers, significantly enhancing exosome incorporation efficiency through synergistic interactions of adhesive forces, hydrogen bonding, and electrostatic interactions [275], as illustrated in Fig. 8F. The electrospun PCL nanofibers were immersed in a dopamine hydrochloride solution, followed by seeding various types of exosomes (derived from milk, MSCs, NSCs). All types of exosomes exhibited sustained release from PDA-PCL nanofibers, in contrast to unmodified PCL nanofibers alone. Similarly, using a straightforward dip-coating strategy, Bui and co-workers loaded pasteurized milk-derived exosomes onto a PDA-coated HA-based electrospun nanofiber matrix, to form an milk exosomes (mEXOs)-incorporated mesh [281]. Briefly, the PDA-coated matrix (PMAT) was prepared by immersing MAT in a dopamine hydrochloride solution. Subsequently, mEXOs were immobilized on PMAT through dipping it into a mEXOs solution. The mesh gradually released mEXOs within fourteen days without any burst release effect.

### 3.5 3D-printed scaffolds

As an additive manufacturing technology, 3D printing enables the precise deposition of a “bioink” composed of biomaterials, growth factors, or EVs onto a freely movable platform to construct biomimetic scaffolds through computer-aided design [282]. This technology presents diverse advantages, such as precise architectural design, adjustable porosity, and high mechanical integrity, which collectively highlight its potential for wound healing [283,284]. The 3D-printed EV-loaded scaffolds can not only preserve the bioactivity of EVs but also prolong their retention time at wound site. Commonly used polymers for bioinks preparation include chitosan [285], alginate [286], hyaluronic acid methacrylate [287], gelatin [288], collagen [289]. Currently, the fabrication of EV-laden 3D-printed scaffolds primarily relies on two strategies: (i) directly 3D printing using bioinks pre-loaded with EVs, and (ii) post-printing loading of EVs into pre-fabricated scaffolds. The following subsections present a detailed discussion of these two approaches.

#### 3.5.1 Direct 3D printing of EVs-containing bioinks

**Fig. 9:**
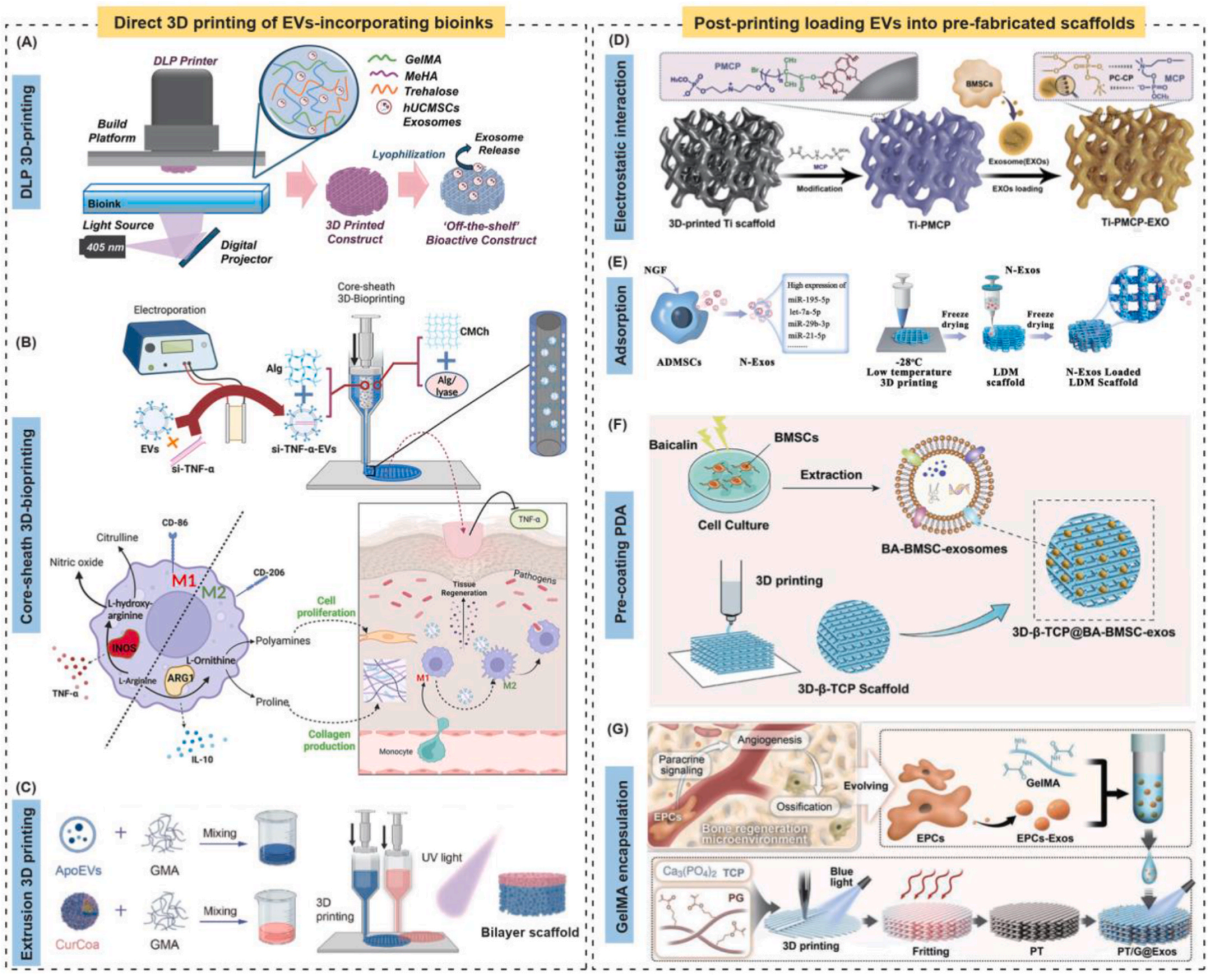
**Representative methods for the fabrication of 3D-printed EV-loaded scaffolds**. (A) Schematic illustration of the fabrication of 3D printing off-the-shelf hepatic patch. Reproduced with permission [290]. Copyright 2025, Elsevier Ltd. (B) Schematic illustration of the preparation of a core-sheath 3D-bioprinted scaffold for the engineered local delivery of si-TNF-loaded EVs. Reproduced with permission [291]. Copyright 2024, Elsevier B.V. (C) Design of a bilayer scaffold utilizing extrusion 3D printing technology. Reproduced with permission [292]. Copyright 2025, Elsevier Ltd. (D) Schematic illustration of Ti-PMCP-EXO scaffold fabrication. Reproduced with permission [293]. Copyright 2025, Americian Chemical Society. (E) Schematic illustration of the fabrication of N-Exo-loaded LDM scaffold. Reproduced with permission [294]. Copyright 2024, Americian Chemical Society. (F) Schematic illustration of the fabrication of 3D-β-TCP@BA-BMSC-exos. Reproduced with permission [295]. Copyright 2025, Elsevier Inc. (G) Schematic showing PT/G@Exos composite scaffolds fabrication. Reproduced with permission [296]. Copyright 2024, Elsevier Ltd.

Direct 3D printing of EVs-encapsulating bioinks enables the construction of the scaffolds with sophisticated architectures, such as core-shell or bilayer structures. For instance, through digital light processing (DLP)-based 3D printing technology, Saha and co-workers developed a off-the-shelf scaffold loaded with exosomes [290]. To develop the 3D bioprinted scaffold, as illustrated in Fig. 9A, DLP 3D printing technology was exploited by using matrix materials with photo-crosslinkable bioinks, composed of GelMA, HAMA, PEGDA, trehalose and exosome, as well as LAP (photo-initiator). The 3D printed scaffold possessed the capacity to improve the stability and retention of the exosomes. In vitro, the developed scaffold exhibited sustained release of hUC-MSCs-Exo.

Through core-sheath 3D bioprinting technology, Vakilian and co-workers developed an innovative core-sheath 3D-printed scaffold incorporating si-TNF-α-loaded EVs as an effective wound dressing [291]. In this system, as illustrated in Fig. 9B, EVs derived from 3T3 fibroblasts were engineered with si-TNF-α using electroporation technique. Subsequently, the scaffolds were fabricated via core-sheath 3D bioprinting technology, with si-TNF-α-EVs suspended in a alginate (Alg) solution serving as the core bioink, while high-viscosity carboxymethyl cellulose sodium salt (CMCh) combined with Alg/lyase served as the sheath bioink. The resultant core-sheath 3D-printed scaffold exhibited a slow initial release of EVs, attributed to the aggregation of EVs within the scaffold matrix. Similarly, Chen and co-workers developed 3D-printed biological scaffolds loaded with interferon gamma (IFN-λ)-preconditioned neural stem cell-derived exosomes (IEs) using collagen/chitosan (CC) as the bioink matrix through low-temperature 3D printing technology [297]. In detail, exosomes were mixed with CC solution with stirring. The resulting hybrid solution was subsequently incorporated into the printer cartridge. Post-printing 3D-CC-IE scaffolds were stored at low temperature, followed by vacuum cooling and drying. The 3D-CC-IE scaffold exhibited a more stable and sustained exosome release compared to the CC-IE group, thereby achieving the prolonged therapeutic effects.

Through extrusion-based 3D printing technology, Jiang and co-workers developed a 3D-printed bilayer scaffold for the sustained release of apoptotic extracellular vesicles (ApoEVs) and antibacterial coacervates to prevent infection for wound healing [292]. Specifically, as illustrated in Fig. 9C, the biolayer scaffold was fabricated using extrusion-based 3D printing technology with two different bioinks: one containing ApoEVs with GelMA to prepare the bottom layer, another containing CurCoa with GelMA to prepare the upper layer. These components mainly interacted through supramolecular interactions (such as hydrogen bonding and electrostatic interaction). The scaffold exhibited anisotropic structure with a distinct bilayer. The pores in the upper layer were obviously smaller than those in the lower layer. The upper layer was thinner, similar to the epidermis, while the lower layer was thicker, simulating dermal tissue. Additionally, the scaffold exhibited sustained ApoEVs release and pH-responsive Cur release behavior. Owing to the sustained release of ApoEVs, the bottom layer can enhance cell proliferation, migration, and angiogenesis.

#### 3.5.2 Post-printing loading EVs into pre-fabricated scaffolds

In addition to directly printing bioinks containing exosomes, another strategy is to integrate exosomes into pre-fabricated 3D-printed scaffolds. This approach employs various loading techniques, such as electrostatic interaction, adsorption, and encapuslation within coating materials like PDA or GelMA. A representative example utilizing electrostatic interaction was demonstrated by Cui and colleagues [293]. As illustrated in Fig. 9D, they engineered a 3D-printed porous titanium (Ti) scaffold functionalized with a bioactive coating of poly[2-(methacryloyloxy)ethyl choline phosphate] (PMCP). The BMSC-derived exosomes were then immobilized onto the scaffold surface through specific electrostatic interactions between the choline phosphate groups of PMCP and the phosphatidylcholine present on the exosomal membrane. Lian and co-workers developed a nerve growth factor (NGF)-preconditioned MSC-derived exosomes (N-Exos)-functionalized 3D-printed hierarchical porous scaffold [294]. Initially, as presented in Fig. 9E, the scaffold was prepared by printing the ink using the low temperature deposition modeling (LDM) printing technique, followed by promptly frozing and subsequently subjected to lyophilization. N-Exos were then dropped onto LDM-printed hierarchical porous scaffold for the adsorption of exosomes into micropores of scaffolds, forming N-Exos/LDM scaffold. The large specific area of the hierachical porous scaffold showed a benefical effect for exosome loading.

Through pre-coating PDA, Zhu and co-workers developed 3D-printed scaffold containing exosomes derived from baicalin (BA)-preconditioned BMSCs (BA-BMSC-exos) [295]. As illustration in Fig. 9F, they used 3D printing technology to construct β-tricalcium phosphate (β-TCP) scaffolds, followed by coating dopamine. Then, BA-BMSC-exos were incorporated into the scaffold, to form 3D-β-TCP@BA-BMSC-exos scaffold. The BA-BMSC-exos were uniformly distributed in the scaffold material. Importantly, in vitro experiments demonstrated that the composite scaffold can continuously release exosomes.

Through GelMA encapsulation method, Kong and co-workers developed a 3D printing scaffold incorporating exosomes derived from endothelial progenitor cells (EPC-exos) [296]. Firstly, as presented in Fig. 9G, PG/TCP (PT) scaffold was fabricated with extrusion-based 3D printer via photopolymerization-based 3D printing method. Subsequently, PT scaffold was filled with GelMA solution containing EPC-exos, followed by blue light crosslinking, thus forming PT/GelMA@Exos (PT/G@Exos) scaffold. The release profiles indicated that GelMA encapsulation was benifical for slow exosome release.

### 3.6 Microspheres

In recent years, microspheres have gained increasing attention in the field of skin wound healing owing to their numerous advantages, such as easy preparation, high stability, and large drug-loading capacity [[298], [299], [300]]. Among various fabrication approaches, microfluidics have emerged as a prominent strategy for the rapid and scalable production of microcapsules with well-defined morphologies [301]. Microspheres are widely utilized as carriers for diverse agents such as small molecule drugs [302,303],growth factors [[304], [305], [306]], and stem cells [[307], [308], [309]], among others. They can encapsulate both solid and liquid therapies, protect them from rapid inactivation, enhance stability, and achieve controlled release efficiency and kinetics [310]. The traditional hydrogel-based drug delivery systems cannot meet the current clinical needs, especially for local injection in small target areas. The emergence of microsphere technology provides a great opportunity for the development of traditional sustained-release hydrogel platforms. The hydrogel microspheres can enhance therapeutic effects, improve clinical outcomes and promote precise drug administration by adjusting particle size and targeted drug delivery, thereby mitigating systemic side effects [311,312]. A key consideration in the preparation of microspheres is to achieve high drug loading efficiency, uniform particle size distribution, and robust batch-to-batch reproducibility while maintaining the inherent biological activity of the encapsulated therapeutic agent [313,314]. Currently, EV-loaded microspheres can be prepared via two primary strategies: (i) one-step fabrication, (ii) post-modification.

#### 3.6.1 One-step fabrication

As a typical example, Kim and co-workers proposed an innovative approach for the controlled EV delivery using monodispersed photodegradable hydrogel microparticles (PHMPs) [315]. To prepare PHMPs, the precursor solution was prepared with a prepolymer solution containing porcine skin-derived gelatin and a crosslinking agent of 4arm-PEG-NB-NHS photocleavable crosslinker. The NHS ester groups of crosslinker facilitated hydrogel formation via reacting with -NH_2_ groups of Gel, leading to stable amide bonds formation. Subsequently, the crosslinker was added to crosslink the polymeric precursor solution, to generate a bulk hydrogel. Finally, MSC-derived EVs were loaded into PHMPs using the droplet-based microfluidic chip. Without external stimuli, EVs were successfully retained within the PHMPs. Furthermore, PHMPs demonstrated the ability to continuously release EVs.

**Fig. 10:**
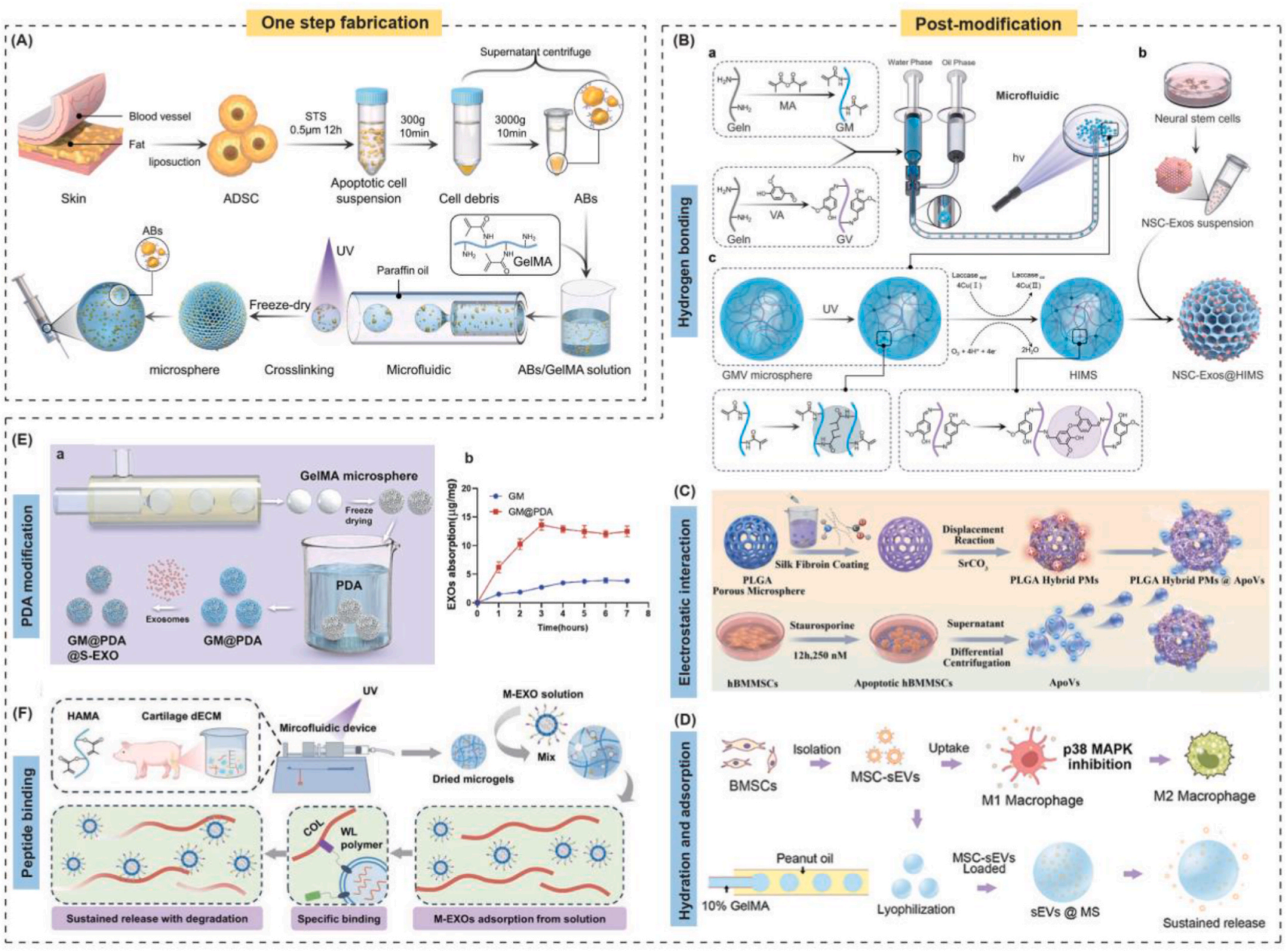
**Representative methods for the fabrication of EV-loaded microspheres**. (A) Schematic illustration of the preparation process of AB@GMSs. Reproduced with permission [55]. Copyright 2024, Elsevier Inc. (B) Schematic representation of (a) the preparation of NSC-Exos@HIMS, (b) harvesting exosomes from NSCs, and (c) the principle of double crosslinking of IPN hydrogel microspheres. Reproduced with permission [316]. Copyright 2025, Wiley-VCH. (C) Schematic illustration of the fabrication of PLGA Hybrid PMs @ApoVs. Reproduced with permission [317]. Copyright 2025, Elsevier B.V. (D) Schematic illustration of the fabrication of sEVs@MS. Reproduced with permission [318]. Copyright 2025, Elsevier Ltd. (E) (a) Schematic illustration of preparation of GM@PDA@S-EXO, (b) exosome loading efficiency curves for microspheres. Reproduced with permission [319]. Copyright 2024, Springer Nature. (F) Schematic diagram of the HECM microgels for targeted and sustained release of exosomes. Reproduced with permission [320]. Copyright 2025, Wiley-VCH.

Through the integration of microfluidic technology and photo-crosslinking processes, Mao and colleagues developed a controlled-release system for apoptotic bodies derived from ADSC (ADSC-ABs) using bioinspired injectable porous PDA-modified GelMA microspheres (AB@GMSs) to improve diabetic wound healing [55]. Briefly, as illustrated in Fig. 10A, ADSC-ABs, induced by staurosporine and purified via differential centrifugation, were encapsulated into GMSs through physisorption, yielding AB@GMSs. Upon local administration of AB@GMSs, ADSC-ABs were gradually released, thereby promoting M2 macrophage polarization. This effect was mediated via JAK-STAT pathway, leading to enhanced ARG1 expression and ultimately facilitating the healing of diabetic wounds.

#### 3.6.2 Post-modification

In addition to one-step fabrication, EV-loaded microspheres can be also prepared through post-modification strategies, such as (i) hydrogen bonding, (ii) electrostatic interactions, (iii) hydration and adsorption, (iv) PDA modification, and (v) peptide conjugation.

Through hydrogen bonding, Zhou and co-workers developed a long-term physically induced hypoxic interpenetrating polymer network (IPN) hydrogel microsphere (HIMS) system with exosomes conjugation [316]. Specifically, the IPN was prepared by a two-step polymerization method. Firstly, GMV microspheres with a certain mechanical modulus were fabricated by using microfluidic technology. After UV crosslinking, secondary enzyme-linking was carried out with different crosslinking degrees and O_2_ modulation capabilities. Moreover, NSC-Exos were coupled to HIMS through hydrogen bonds, as illustrated in Fig. 10B. In vitro, HIMS showed a stable retention and continuous release of exosomes.

Through electrostatic interaction, Liu and co-workers incorporated apoptotic vesicles (apoVs) into PLGA porous microspheres (PMs) [317]. Specifically, PLGA PMs were fabricated by the double emulsion-solvent evaporation method. Subsequently, strontium (Sr) was deposited on the silk fibroin (SF)-coated PLGA PMs through chemical displacement, and then hBMMSC-derived apoVs were encapsulated into the PLGA hybrid PMs through electrostatic adsorption, as illustrated in Fig. 10C. After apoVs incorporation, the zeta potential of the PLGA hybrid PMs changed from a positive charge to a negative charge, confirming the electrostatic interaction between the hybrid PMs and apoVs. Liu and co-workers developed porous microspheres to deliver gingival MSC-derived exosomes (GMSC-Exo) for promoting vascularized diabetic wound healing [35]. In this system, the pre-prepared microspheres were employed to electrostatically attract negatively-charged exosomes. The microspheres, composed of PLGA, poly-L-lactide-poly (ethyl glycol)-poly-L-lactide (PLLA-PEG-PLLA), nano-hydroxyapatite (nHAP), and poly-ε-L-lysine (EPL), were prepared using a double emulsion method, followed by alkali treatment and EPL coating. The EPL-coated microsphere exhibited a positive charge, while decreased after GMSC-Exo loading. This indicated that the porous microspheres exhibited a positive surface charge after EPL coating, enabling efficient adsorption of negatively-charged exosomes. In vivo studies demonstrated that GMSC-Exo-loaded porous microspheres significantly accelerated diabetic wound healing by facilitating re-epithelialization, collagen deposition, and angiogenesis.

Through a rehydration-induced swelling strategy, Li and co-workers incorporated MSC-derived sEVs (MSC-sEVs) into lyophilized GelMA microspheres for enhancing diabetic wound healing [318]. Specifically, GelMA microspheres were prepared via microfluidic technology. Subsequently, the lyophilized microspheres were freeze-dried, followed by incubating with MSC-sEVs to allow full hydration and adsorption, as shown in Fig. 10D. The microspheres exhibited continuously release MSC-sEVs. In vivo, sEVs@MS treatment promoted wound healing, enhanced M2 macrophage polarization, promoted collagen formation and epidermal regeneration, and suppressed inflammation.

Through PDA modification, Cao and co-workers developed a hydrogel microsphere loaded with exosomes rich in superoxide dismutase 3 (S-EXOs) [319]. Initially, as presented in Fig. 10E(a), GelMA microspheres were prepared by the integration of microfluidics and photo-crosslinking. Then the as-prepared microspheres were soaked in a PDA solution, forming GM@PDA. Subsequently, S-EXOs were mixed with microspheres to form GM@PDA@S-EXO. After PDA modification, the average size of the microspheres significantly increased, and exosome loading in microspheres also increased, as presented in Fig. 10E(b). Moreover, GM@PDA microspheres released slowly over fourteen days. In another study, Gao and co-workers developed an injectable porous PLGA microsphere with PDA modification (PMS-PDA) for loading hypoxia-preconditioned exosomes [321]. The average size of PMS-PDA microsphere was bigger than that of PMS microsphere. Moreover, the porous architecture combined with PDA-modified surface enabled PMS-PDA microsphere to achieve a high exosome loading capacity. Furthermore, the PMS-PDA microsphere demonstrated sustained exosome release.

Through a peptide conjugation strategy, Deng and co-workers developed an integrated system comprising self-assembled bifunctional exosomes (M-EXO) and decellularized cartilage-based microgels (HECM) [320], as shown in Fig. 10F. The M-EXO was fabricated by incubating UCMSC-derived exosomes with cholesterol-PEG-peptide polymers via a one-step self-assembly process. Meanwhile, HECM microgels were prepared by mixing cartilage decellularized matrix (dECM) with HAMA, forming pre-gel droplets via microfluidics, UV crosslinking and dehydration processes. The composite HECM@M-EXO was obtained through rehydrating dried HECM microgels in a M-EXO solution, enabling a stable encapsulation via WL-collagen II binding. This combination prevented the easy diffusion of M-EXO out of HECM microgels and achieved continuous release during the degradation of the HECM microgels.

### 3.7 Effect of biomaterial properties on EV identify and biological activity

The encapsulation, immobilization, or adsorption of EVs onto biomaterials are not biologically neutral processes. Physicochemical properties of biomaterials (such as matrix stiffness, matrix viscoelasticity, mechanical stress, topographical structure, internal porosity, surface charge, and degradation rate) directly remodel EV characteristics (e.g., particle size, yield, membrane proteins, cargo composition, and biological function) by pre-treating parent donor cells and interacting with the secreted EVs.

#### 3.7.1 Alterations in EV identity

(i) **Alterations in EV particle size and yield**: Matrix viscoelasticity can reduce the particle yield, increase the particle size, and increase the protein content per particle of BMSC-derived exosomes [322]; static topographical structure combined with dynamic fluid stimulation can increase the yield of macrophage EVs [323]; mechanical squeezing effect induced by a microfluidic device can increase the yield of sEVs from the BM-MSCs by approximately four-fold, while preserving the growth status of stem cells [324].
(ii) **Changes in EV membrane proteins**: Hydrophobic interactions, electrostatic interactions, or hydrogen bonds between matrix and EVs can all lead to protein denaturation, unfolding, or competitive substitution. For example, encapsulation of a single EV within a selectively disassemblable protective shell composed of metal-phenolic networks (MPNs) followed by post-modification with polyethylene glycol (PEG), leads to a detectable reduction in CD9 levels, likely due to shielding effect of encapsulation layer [163]. Moreover, softer silica nanoparticles significantly regulated exosomal markers (CD63, TSG101, and ALIX) compared with stiffer silica nanoparticles [325].
(iii) **Changes in EV cargo composition**: The internal cargo of EVs (e.g., proteins, miRNAs, mRNAs) can also be impacted. The processes of encapsulation or surface conjugation can exert physical forces (e.g., shear stress) or alter the local chemical microenvironment, potentially causing the degradation or leakage of these sensitive biomolecules. mechanical stress can enhance the angiogenic protein cargo of fibroblast-derived exosomes [326]; 3D mechanical stimulation reshapes the molecular cargo of endothelial exosomes, enhancing their biomechanical communication between endothelial cells and fibroblasts [327].

#### 3.7.2 Modulation of EV biological activity

Mechanical cues (such as matrix stiffness, matrix viscoelasticity, and mechanical stress) can influnce the physicochemical properties of EVs, which in turn may modulate their function and biological effects. This highlights theessential role of physical cues in EV-mediated cellular communication. Studies have shown that EVs derived from stiff matrices exhibit a greater capacity to promote cell proliferation compared with those derived from soft matrices [328]. Moreover, the matrix viscoelasticity have been shown to significantly enhance the regenerative potential of EVs, including the promotion of cell proliferation and migration, the induction of angiogenesis, and the alleviation of inflammation [322]. Additionally, a study shown that compared to the soft hybrid vesicles, the hard hybrid vesicles (milk exosomes-liposomes hybrid vesicles) have a reduced ability in penetrating mucus, but they demonstrate significantly higher efficacy in penetrating the epithelial cell barrier [329]. Mechanical stress has also been reported to faciliate angiogenesis by regulating the secretion and functionality of exosomes [330]. Collectively, these findings demonstrate that the physicochemical properties of biomaterials can regulate the secretion phenotype of EVs, thereby offering therapeutic potential for diabetic wound management.

## 4 Release of EVs from biomaterials

### 4.1 Release mechanisms

Understanding the release mechanism governing EVs from biomaterials is essential for the rational design of controlled EV delivery systems. The main release mechanisms of EVs include three types: (i) diffusion-controlled release, (ii) swelling/shrinking-controlled release, (iii) degradation/erosion-controlled release. The following subsections will outline these key release mechanisms and presents the mathematical models used for analysis.

#### 4.1.1 Diffusion-controlled release

**Fig. 11:**
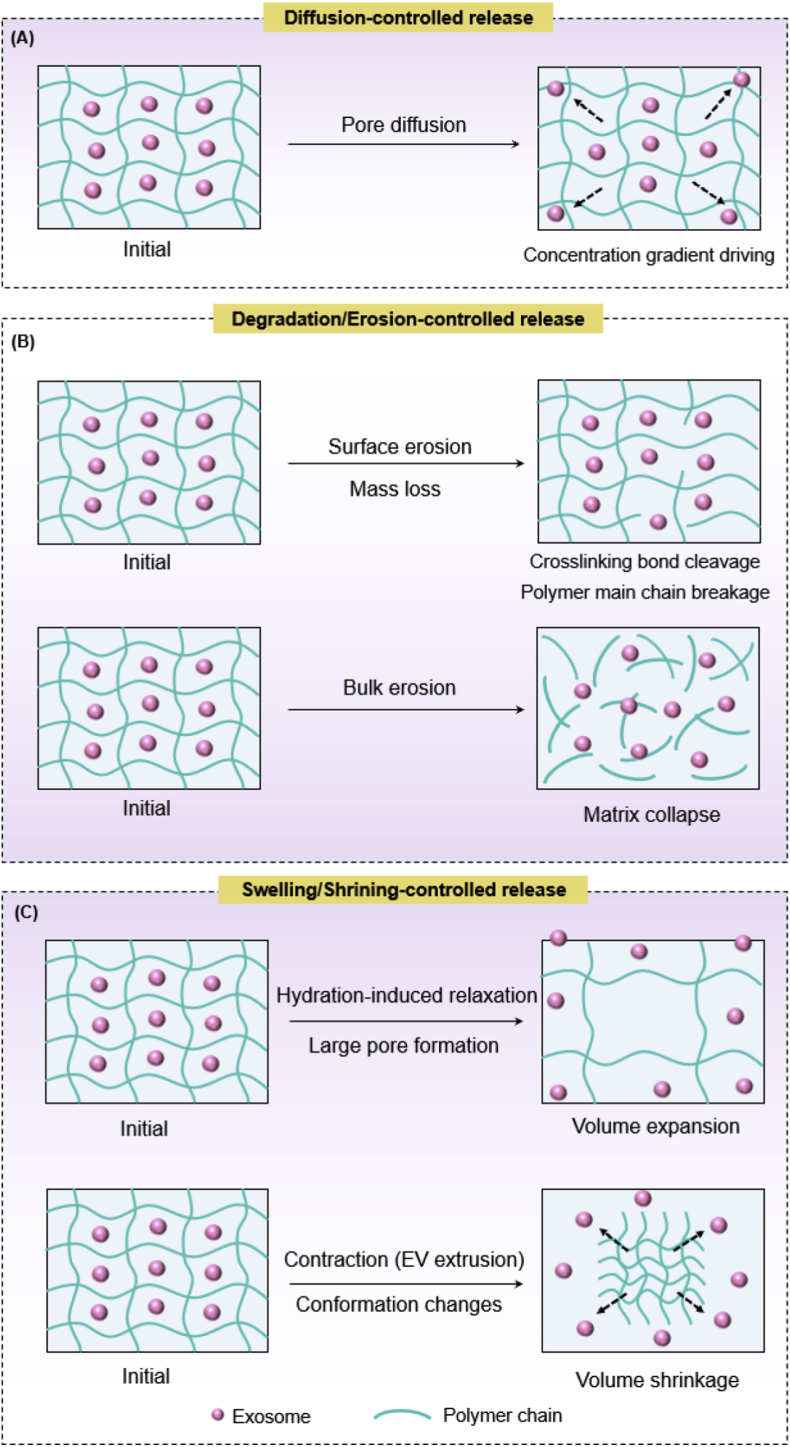
Schematic illustration of release mechanisms of EVs from biomaterials: (A) diffusion-controlled release, (B) swelling/shrining-controlled release, and (C) degradation/erosion-controlled release.

In the diffusion-controlled release system, the release of EVs is achieved through their diffusion within the polymer membrane or polymer matrix. These systems can be classified as porous diffusion and non-porous diffusion. As illustrated in Fig. 11A, the diffusion-controlled release is driven by concentration gradients [331], which facilitates the movement of EVs to the wound site. The release kinetics are predominantly governed by the structure of polymer matrix and the physiochemical attributes of EVs, while the carrier material does not undergo chemical degradation or significant expansion [332]. Under this mode, the release of EVs follows a “parabolic” curve: the initial release rate of EVs is very high, which is attributed to the rapid diffusion of EVs on and near the surface; then it enters a plateau phase, which is controlled by the migration of EVs from the deep part of the matrix through tortuous channels. In this mechanism, the integrity of EVs can be maximally preserved, but the release dose is limited by the diffusion boundary layer.

#### 4.1.2 Swelling/shrinking-controlled release

Swelling refers to the volumetric expansion and structural dilation of scaffolds when immersed in solvent environments [333]. This process promotes the formation of larger pores within the scaffold architecture, threby enabling the controlled diffusion of encapsulated EVs. The fundamental mechanism underlying swelling-controlled EV release involves the hydration-induced relaxation of the polymer carrier network. The consequent relaxation of polymer chain segments leads to an expansion of network mesh size, which in turn triggers a volume phase transition of the scaffold. The transition subsequently facilitates the diffusive release of EVs. Among the various swelling-driven mechanisms, electrostatic repulsion emerge as the predominant contributor.

In contrast, shrinking is the volume shrinkage and dehydration of scaffolds, which induce smaller pore formation and further cause the extrusion of EVs from the scaffolds [332], as illustrated in Fig. 11C. This mechanism involves the reversible or irreversible changes in the secondary structure or chain conformation of polymer chains (e.g., from a helix structure to a random coil sturcture [334]) or hydrophilic-to-hydrophobic phase transition [335]. These conformational changes enable the delivery platform with continuous release of EVs. This mechanism invovles the rearrangement of intramolecular or intermolecular non-covalent interactions (such as electrostatic interaction, hydrogen bonds, hydrophobic interaction) [334,336,337], which leads to a change in the overall spatial arrangement of the polymer chain. Additionally, several thermo-responsive polymers, such as poly (N-isopropylacrylamide) (PNIPAM) and poly (N-vinylcaprolactam) (PNVCL), can achieve conformation-controlled release of EVs through the swelling/shrinkage equilibrium of polymers. For instance, Li and co-workers prepared a mechanically active hydrogel by integrating PNIPAM polymer chain into the main network of hydrogel for regulating the release of ADSC-derived exosomes loaded into the hydrogel [338]. When the temperature is above the lower critical solution temperature (LCST), the hydrophobic segments of PNIPAM function, causing the contraction and reduction in volume of the hydrogel network structure and further inducing the release of exosomes. Similarly, Xie and co-workers fabricated a self-contraction microgel assembly with robust tissue adhesion (SMART) via the improved sedimentation polymerization of N-isopropylacrylamide (NIPAM) and acrylic acid (AAC), which can on-demand release of hMSC-derived exosomes [339]. Upon surpassing the LCST of SMART, the disruption of hydrogen bonds between the amide and isopropyl groups induced the shrinkage of chains segments and the bulk volume of SMART, thereby causing the release of the exosomes for re-epithelialization and wound mciroenvironment regulation.

Additionally, relevant studies have shown that the cholesterol-modified deoxyribonucleic acid (DNA) single stands undergo intramolecular folding under acidic conditions, forming unstable i-motif structures. This structural change will disrupt hydrogen bonds within the DNA duplex, which inspires researchers to design delivery systems based on the comformational transformation of DNA, achieving the sustained release of EVs in the acidic diabetic wound microenvironment. For instance, Feng and co-workers fabricated a self-adapting DNA-crosslinked silk fibroin hydrogel encapsulating BMSC-derived exosomes for promoting diabetic wound healing [340]. The hydrogel exhibited pH-sensitive exosome release properties by acidic pH-induced duplex-to-i-motif transition of cholesterol-modified DNA.

#### 4.1.3 Degradation/erosion-controlled release

Degradation involves the gradual breakdown or transformation of chemical compounds into simpler, more stable, or less harmful forms within environmental or biological systems. Erosion-controlled release relies on the biodegradation or physical erosion of carrier materials, during which the EVs are gradually released as the carrier degrades. The release of EVs mainly depends on the chemical breakage of the polymer main chain or crosslinking bonds, which leads to the disintegration or erosion of the material. The molecular weight of the polymer continues to decrease, mechanical strength is lost, and eventually there is a loss of mass (erosion). This is an irreversible chemical process. This process is generally categorized into surface erosion (anomalous degradation) and bulk degradation (homogeneous degradation) [333,341], as illustrated in Fig. 11B. When EVs are bonded by sensitive chemical bonds (such as disulfide bonds, borate ester bonds, Schiff-base bonds) or uniformly encapsulated in degradable polymers, their release rate is dominated by the rate of mass loss of matrixes. For instance, He and co-workers constructed a multifunctional OHAMA-EPL (OE) hydrogel encapsulating MSC-derived sEVs [342]. The acid-responsive degradation of Schiff base in OE-Gel facilitates the sEVs controlled release. If it is surface erosion, the curve shows zero-order release, which is a linear constant-speed release mode of the delivery system; if it is bulk degradation, a “bulk erosion” phenomenon usually occurs in the middle stage due to the collapse of the matrix.

Additionally, the intricate pathophysiological milieu of chronic wounds has spurred the development of stimulus-responsive delivery systems for EVs. Such systems offer notable advantages in preserving EVstability and prolonging their retention at wound sites. Stimulus-responsive systems are activated by specific stimuli and are designed by incorporating various responsive polymers or sensitive chemical bonds that respond to either endogenous signals (e.g., pH [158,207,343], matrix metalloproteinases [[344], [345], [346], [347]], glucose [[348], [349], [350]], reactive oxygen specifies [[351], [352], [353]]) or exogenous triggers (e.g., temperature [354], light [[355], [356], [357]]). These designs enable the precise and targeted release of EVs from carrier materials upon changes in the wound microenvironment or external cues. Furthermore, Table 1 presents a comprehensive overview of stimulus-responsive hydrogels that enable the sustained release of EVs for promoting wound healing.

**Table 1:** A comprehensive summary of stimuli-responsive hydrogels with sustained release of EVs for wound healing.

Type of stimuli-responsive	Hydrogel composition	Hydrogel degradation	EV release behavior	Wound model	Wound efficacy	Refs.
pH	BMSC-Exos, L-Arg, DNA-Chol	• pH-dependent biodegradation: faster degradation rate at pH 6.0 than at pH 7.4	• Within 24 h: 97% released (pH 6.0), 82% released (pH 7.4)	6-mm-diameter full-thickness wound on STZ-induced diabetic mice	• Fast wound closure by day 14 • Achieve complete epithelial regeneration • Promote re-epithelialization • Enhance collagen maturation • Mitigate inflammation • Enhance angiogenesis • Modulate immune microenvironment	Feng et al. (2026) [340]
	HUCMSC-Exo, HAL, AHA, ODex, SCS	• Degrade slowly with 38.29 ± 3.79% mass loss at day 28	• ∼60% released (pH 5.5) within 7 days, 83.43 ± 5.04% released (pH 7.4) by day 15 and approaching completion by day 21	12-mm-diameter full-thickness wound on SD rats	• Fast wound closure by day 14 • Enhance granulation tissue formation • Promote angiogenesis • Modulate the immune microenvironment	Zhai et al. (2026) [358]
	ApoVs, MXene, PEDOT, PSS, PVA	• Not applicable	• Within 24 h: ∼55.04% released (pH 5.5), ∼40.14% released (pH 7.4)	10-mm-diameter full-thickness wound on STZ-induced diabetic rats	• Fast wound closure by day 14 • Promote collagen deposition • Enhance angiogenesis • Good antibacterial effects	Yan et al. (2026) [359]
	AMSCs-exo, F127, OHA, EPL	• Faster degradation rate at pH 5.5 than at pH 7.4	• Higher release rate at pH 5.5 than at pH 7.5	8-mm-diameter full-thickness wound on STZ-induced diabetic mice	• Complete wound closure and hair growth at day 21 • Promote skin appendage regeneration • Improve collagen deposition • Promote angiogenesis and blood vessels formation • Promote cell proliferation	Wang et al. (2019) [360]
	sEVs^miR^, AlgHA, MFE	• pH-responsive degradation properties: >80% degraded at pH 5.0 within the first 6 days	• >80% released (pH 5.0) in the first 4 days • Higher release rate at pH 5.0 than at pH 7.4	15-mm-diameter full-thickness wound infected with S. aureus on STZ-induced diabetic rats	• 97.9% wound closure by day 14 • >95% bacterial killing rate on day 3 • Complete epidermal regeneration by day 14 • Promote collagen deposition • Antioxidant stress effect • Alleviate endoplasmic reticulum stress • Promote angiogenesis • Promote macrophage M2 polarization	Zhang et al. (2025) [343]
	MSC-sEVs, OHAMA, EPL	• >85% degradation ratio within 3 days	• >80% released by day 3 • Higher release rate at pH 6.0 than at pH 7.4	7-mm-diameter full-thickness wound infected with MRSA on Kunming mice	• 92.63% wound healing rate by day 14 • Promote skin appendage (hair follicle growth) formation • Promote collagen fiber deposition • Antibacterial and anti-inflammation effect • Promote cell proliferation • Promote angiogenesis	He et al. (2025) [342]
	Cur-Exos, PEG-BA, CMC	• pH-responsive degradation behavior: Faster degradation at pH 5.5 than at pH 7.4	• Faster release at pH 5.5 than at pH 7.4	15-mm-diameter full-thickness wound on STZ-induced diabetic rats	• Reduce wound areas • Enhance epithelial migration and collagen deposition • Decrease M1 macrophage activity	Li et al. (2025) [361]
ROS	Exos, OAlg, CP	• ROS-responsive degradation behavior: >80% degraded in H2O2 solution within 7 days	• Within 7 days: 84.37 ± 1.39% released in H2O2 solution, while 66.27 ± 3.75% released in PBS	8-mm-diameter full-thickness wound on STZ-induced diabetic mice	• Nearly complete healing (97.97 ± 0.90%) by day 14 • Abundant newly formed hair follicles • Promote collagen deposition • Suppress excessive inflammatory responses • Promote cell proliferation	Lin et al. (2026) [362]
	hUC-MSCs-Exos, VP, PVA, TSPBA, PLGA, EGCG	• H_2_O_2_-dependent degradation kinetics	• Over 15 days: 79.9 ± 1.6% released in 1 mM H_2_O_2_	6-mm-diameter full-thickness wound on STZ-induced diabetic mice	• *Complete re-epithelialization by day 14* • *97.85% inhibition rate on bacterial biofilm formation on day 3* • *Achieve dynamic ROS modulation* • *Improve collagen maturation and appendage regeneration* • *Promote angiogenesis* • *Shift toward pro-regenerative M2 macrophage polarization* • *Promote scar-free healing*	Du et al. (2026) [363]
	vEVs, PVA, P-MG	• ROS-responsive cleavage behavior: ∼90% degraded gradually over 10 days	• Within 8 days: 90% released in H_2_O_2_ solution, while ∼40% released in PBS • Faster and higher release rate in H_2_O_2_ than in PBS	6-mm-diameter full-thickness wound on STZ-induced diabetic mice	• 91.3% wound closure by day 14 • Accelerate re-epithelialization • Enhance collagen deposition • Mitigate excessive inflammation	Ding et al. (2026) [364]
MMP	M2-Exo, CMCS, ODex, PG-6	• MMP-9-dependent degradation behavior: 60% degraded in 50 ng/mL MMP-9 within 20 days	• Higher release amount in 50 ng/mL MMP-9 than in 10 and 20 ng/mL MMP-9 solution	10-mm-diameter full-thickness wound on db/db mice	• Fast wound closure by day 21 • Promote collagen deposition • Reduce chronic inflammation	Meng et al. (2025) [347]
	ADSC-exo, MMP-PEG	• MMP-2-dependent degradation behavior: within 20 days, >60% degraded with MMP-2, and <10% degraded in PBS	• Within 20 days: nearly 90% released with MMP-2, while a little released in PBS	10-mm-diameter full-thickness wound on STZ-induced diabetic mice	• Fast wound closure rate by day 14 • Promote re-epithelialization and collagen deposition • Promote cell proliferation • Promote neovascularization	Jiang et al. (2022) [365]
Glucose	hUC-MSC-EXO, HAMA, CSMA, MPBA	• Not applicable	• Higher release rate in 4.5 g/L glucose solution than in PBS • >80% released within 14 days	Full-thickness wound on STZ-induced diabetic mice	• ∼95% wound closure by day 12 • Promote the growth of granulation tissue and collagen remodeling • Enhance angiogenesis • Promote the polarization of M2 macrophages • Reduce inflammation	Yuan et al. (2024) [350]

Temperature	EVs, PEG, MC	• Not applicable	• Faster release rate at 29 °C than at 37 °C • 99.9% released at 29 °C within 5 days • 100% released within 8 days at 37 °C	Mouse hindlimb ischemia model	• Improve angiogenic capability • Improve functional regeneration • Enhance limb rescue rate • Reduce foot necrosis rate • Inhibit muscle damage and inflammation • Enhance neovascularization via activation of VEGF/VEGFR pathway • Reduce muscle damage via activation of ALP	Xing et al. (2021) [366]
	MSC-EXO, NIPAM, AAC	• Not applicable	• Higher release rate at 37 °C than at 0 °C • >90% unimpeded release over 10 days • Sustained release in the initial 3 days at 37 °C	8-mm-diameter full-thickness wound on db/db mice	• Fast wound closure rate by day 16 • Promote re-epithelialization • Promote collagen deposition • Promote angiogenesis • Activate skin mechano-transduction	Xie et al. (2024) [339]
Light	hucMSC-Exo, Ti_3_C_2_ MXene, Agarose hydrogel	• ∼30% degraded over 15 days in PBS at 37 °C	• Higher release amount with NIR laser irradiation than without NIR laser irradiation • Almost no release without NIR laser irradiation	10-mm-diameter full-thickness wound on Balb/c nude mice	• Almost complete wound closure by day 13 • Promote collagen deposition and remodeling • Promote angiogenesis • Reduce inflammation	Teng et al. (2024) [357]
	M2-Exos, AuNRs, Aam, NIPAM, MPBA	• ∼30% degraded in type I collagenase solution within 16 days	• Faster release rate with NIR-irradiation than without NIR-irradiation • ∼80% released within 9 days with NIR-irradiation	10-mm-diameter full-thickness wound on STZ-induced diabetic mice	• Fast wound healing rate by day 12 • Promote re-epithelialization and collagen deposition • Promote angiogenesis • Suppress inflammation	Li et al. (2023) [367]
pH/Glucose	ADMSCs-Exos, CS	• Not applicable	• Fastest release rate at pH 5.5 among different pH values • Faster release rate in high-glucose solution	10-mm-diameter full-thickness wound on STZ-induced diabetic mice	• 90% ± 5% wound healing rate by day 14 • Promote re-epithelialization • Promote collagen deposition • Enhance neovascularization	Song et al. (2026) [368]
	Exos, GOx, CMCS, OPLL	• Faster degradation rate in 3 mg/mL glucose solution than in sugar-free PBS solution • >80% degraded within 24 h in 3 mg/mL glucose solution	• Faster release rate in pH 7.4 than in pH 5.5 • Faster release rate in 3 mg/mL glucose solution than in sugar-free PBS solution	10-mm-diameter full-thickness wound on STZ-induced diabetic mice	• Complete healing by day 10 • Promote collagen deposition • Reduce inflammation • Promote neovascularization • Promote M1 to M2 macrophage polarization • Regulate cellular oxidative stress	Yang et al. (2024) [207]
ROS/Glucose	hUCMSC-Exo, CS-PBA, PVA, DFO, Mn-ZIF-8	• ROS/Glucose dual-responsive degradation behavior: ∼75% degraded at 3 mg/mL glucose with 1 mM H_2_O_2_ within 10 day	• •Within 7 days: 87.56% released at 3 mg/mL glucose with 1 mM H_2_O_2_, while • 74.40% released at 3 mg/mL glucose without H_2_O_2_ • •Within 24 h: 59.37% released at 3 mg/mL glucose with 1 mM H_2_O_2_, while 37.5% released at 3 mg/mL glucose solution	10-mm-diameter full-thickness wound infected by S. aureus on STZ-induced diabetic mice	• 99.41% wound closure rate at day 14 • Promote re-epithelialization • Reduce inflammation • Promote angiogenesis • Promote cell proliferation	Lin et al. (2026) [369]

pH/ROS/Glucose	Exo, LAMC	• 34.5% degraded in a pH = 9 environment • 36.7% degraded in the presence of H_2_O_2_ • 34.6% degraded in 10 mg/mL glucose solution	• Threefold increased release in the presence of environmental stimuli such as pH = 9, high glucose levels, or H_2_O_2_	10-mm-diameter full-thickness wound on STZ-induced diabetic mice	• Fast wound healing rate over 21 days • Promote collagen deposition • Anti-oxidative stress properties • Promote angiogenesis • Inhibit AGEs formation	Liu et al. (2023) [370]

***Abbreviations***: L-arginine (L-Arg); hexaminolevulinate hydrochloride (HAL); aminated hyaluronic acid (AHA); oxidized dextran (ODex); epigallocatechin gallate (EGCG); Verteporfin (VP); poly(lactic-co-glycolic acid) (PLGA); N^1^-(4-boronobenzyl)-N^3^-(4-boronophenyl)-N^1^, N^1^, N^3^, N^3^-tretramethylpropane-1,3-diaminium (TSPBA); sulfated chitosan (SCS); apoptotic bodies (ApoVs); polyvinyl alcohol (PVA); miR-210-3p-engineered small extracellular vesicles (sEVs^miR^); mulberry fruit extract (MFE); methacrylate-modified oxidative hyaluronic acid (OHAMA); poly-ε-L-lysine (EPL); oxidized sodium alginate (OAlg); carboxymethyl chitosan-grafted phenylboronic acid (CP); 4-carboxyphenylboronic acid-modified chitosan (CS-PBA); desferrioxamine (DFO); lipoic acid modified chitosan (LAMC); advanced glycation end products (AGEs).

### 4.2 Release profiles

**Fig. 12:**
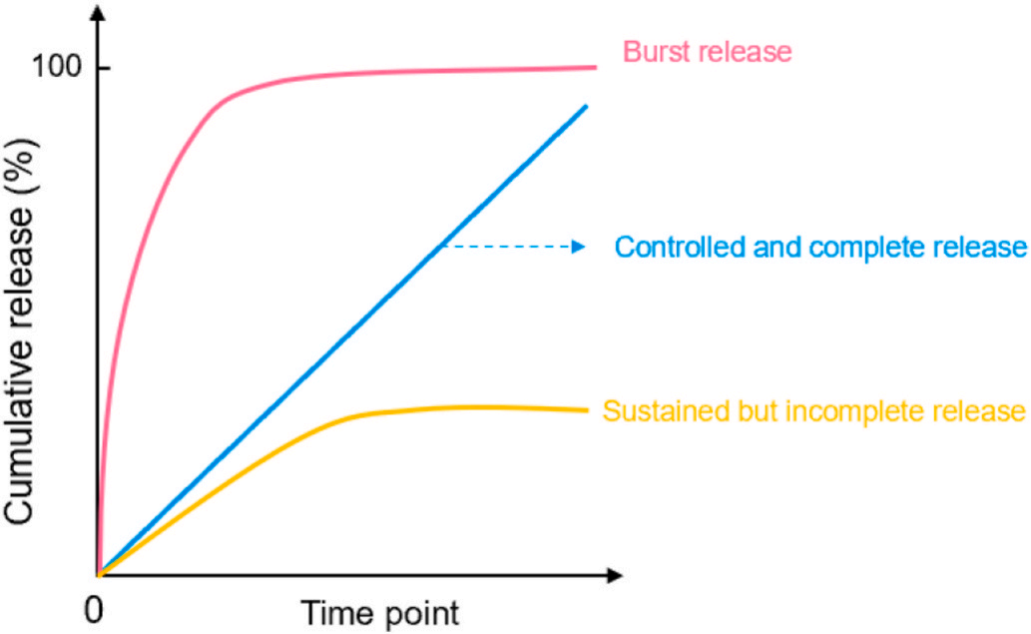
Schematic illustration of three main pattern of EV release profiles.

Three main patterns of EV release profiles from carrier materials, including (i) burst release, (ii) sustained but incomplete release, and (iii) controlled and complete release, are shown in Fig. 12. Controlled release refers to the process where EVs are released from specific internal sites or regions at an appropriate time points and following a predetermined release rate. Burst release refers to the phenomenon where EVs are released rapidly and intensely. Continuous release refers to the prolonged and fixed-rate release process of EVs. Lag time occurs during the delayed release process.

### 4.3 Release kinetic models

**Fig. 13:**
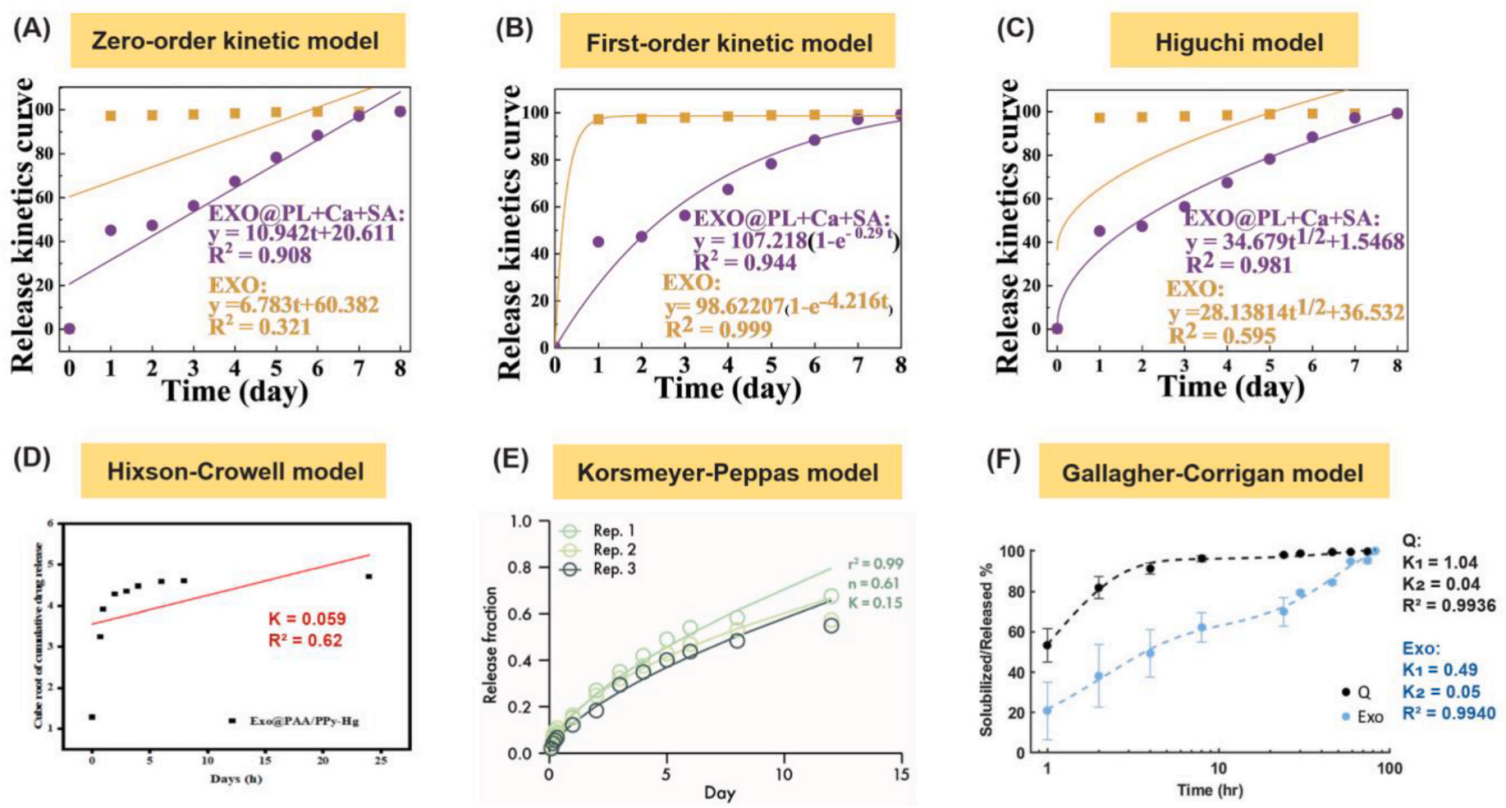
**Different mathematical kinetic models applied to fitting EV release profiles to analyze release mechanisms.** Cumulative EVs release profiles from biomaterials carrying EVs and the corresponding fitted curves with (A) Zero-order, (B) First-order, and (C) Higuchi models. Reproduced with permission [371]. Copyright 2026, Elsevier B.V. (D) Hixson-Crowell model. Reproduced with permission [372]. Copyright 2025, Elsevier B.V. (E) Korsmeyer-Peppas model. Reproduced with permission [373]. Copyright 2025, Elsevier Inc. (F) Gallagher-Corrigan model. Reproduced with permission [157]. Copyright 2026, Elsevier Ltd.

To investigate the release mechanism of EVs in various biomaterial-based delivery systems, the most common mathematical models, including zero-order kinetic model, first-order kinetic model, Hixson-Crowell, Higuchi model, Korsmeyer-Peppas or Ritger-Peppas model, Peppas-Sahlin model, and Gallagher-Corrigan model, are employed to quantitatively analyze the release profiles of EVs from carrier materials under varying physiochemical conditions, enabling a systematic analysis of the material performance. The representative mathematical kinetic models applied to fitting EV release profiles are presented in Fig. 13, thereby facilitating the analysis of release mechanisms. Moreover, these kinetic models can effectively describe the underlying release mechanisms of EVs from carrier materials (such as hydrogels, microneedle patches, electrospun membranes, 3D-printed scaffolds or microspheres), and even can explain complex phenomena such as diffusion, erosion, and expansion. The following subsections will conduct a comprehensive analysis of these models comprehensively, emphasizing their principles and significance in EV delivery systems.

#### 4.3.1 Zero-order kinetic model

The Zero-order kinetic model is also known as steady-state release, as its release rate remains constant and is independent of concentration, thus enabling a predictable and controllable release process [332]. equation (1) is as follows:

(1) $$W_{t}=W_{0}+k_{0}\cdot t$$

where W_t_ and W_0_ represent the mass of EVs at the testing time and initial time, respectively, as reflected by the cumulative release percentage; k_0_ is the zero-order rate constant. For instance, sevval and co-workers developed M2-macrophage-derievd EVs-functionalized acellular dermal matrix. The release kinetics exhibited a near-liner profile, with a correlation coefficient of R^2^ = 0.9761, suggesting a controlled diffusion behavior likely mediated by the porous ECM network [374].

#### 4.3.2 First-order kinetic model

The First-order kinetic model describes EV release as concentration-dependent. The EV release rate decreases as the concentration declines, often in proportion to the concentration of the EVs, following the Fickian diffusion principle. This model characterizes a concentration-dependent release rate [375,376], and typically empolyed to describe the release of EVs from polymeric matrices, porous carriers, or solution-based systems [377]. equation (2) is as follows:

(2) $${\text{logC}}_{t}={\text{logC}}_{0}-\frac{k_{1}\cdot t}{2.303}$$

where C_t_ and C_0_ are the concentration of EVs at the testing time and initial time, respectively; k_1_ is the first-order rate constant. For instance, Yang and co-workers applied three kinetic models (Zero-order, First-order, and Higuchi) to fit the cumulative release profiles of exosomes from the EXO@PL + Ca + SA hydrogel system [371]. As shown in Fig. 13A–C, the release behavior of the EXO group exhibited a strong concordance with the first-order model, suggesting a significant dependence of the release rate on the remaining exosome concentration. In contrast, the EXO@PL + Ca + SA group demonstrated a better alignment with the Higuchi model, suggesting that the release process was governed predominantly by diffusion and followed classic Fickian behavior. Huang and co-workers employed four kinetic models (Zero-order, First-order, Higuchi, and Ritger-Peppas) to fit the cumulaive release profiles of EVs from MSC-EVs-GEL, MSC-EVs-GEL (4 °C), FAP-KO-MSC-EVs-GEL, and BV2-EVs-GEL [378], respectively. The release kinetics of MSC-EVs-GEL and FAP-KO-MSC-EVs-GEL were found to be more consistent with the first-order model, with correlation coefficients of R^2^ of 0.9990 and 0.9652, respectively. Conversely, the release profiles of MSC-EVs-GEL (4 °C) and BV2-EVs-GEL had a superior fit with the Ritger-Peppas model, yielding R^2^ values of 0.9829 and 0.9791, respectively. It was well established that the release characteristics of hydrogels are joinyly governed by dissolution and diffusion processes, which is consistent with the Ritger-Peppas model. For MSC-EVs-GEL, since the enzymes present on the membrane of MSC-EVs are capable of mediating hydrogel degradation, thereby accelerating EV release, the Ritger-Peppas release model is switched to a first-order release model. The enzymes on MSC-EVs membranes play a pivotal role in modulating the release of EVs from the hydrogel matrix.

#### 4.3.3 Hixson-Crowell model

The Hixson-Crowell model, originally proposed by Hixson and Crowell in 1931 [379], is widely empolyed to characterize drug release kinetics from polymeric matrices. In this model, parameters such as the geometry, surface area, and particle size of the polymer exert significant influence on the release behavior [377,380]. This model postulates a linear relationship over time between the difference in the cube roots of the initial drug mass and the remaining drug masses [381]. This model can be also used for evaluating EV release mechanism from polymeric systems. equation (3) is as follows:

(3) $$C_{0}^{1/3}-C_{t}^{1/3}=k_{\text{hc}}\cdot t$$

where C_0_ is the initial amount of EVs; C_t_ is the amount of EVs released at the testing time; k_hc_ is a rate constant related to the surface area and volume [382].

#### 4.3.4 Higuchi model

The Higuchi model, proposed by Takeru Higuchi in 1961, defines the time-dependent variation of drugs release from semisolid or solid matrices based on Fickian diffusion [383]. equation (4) is as follows:

(4) $$Q_{t}=k_{h}\cdot \sqrt{t}$$

where Q_t_ represents the cumulative amount of EVs released at the testing time and k_h_ is the Higuchi dissolution constant. For instance, Xu and co-workers utilized five kinetic models (zero-order, first-order, Hixson-Crowell, Higuchi, and Korsmeyer-Peppas) to investigate the exosome release mechanisms [372]. They found that Higuchi model presented the highest value of R^2^ of 0.95 (Fig. 13D), suggesting that Higuchi model showed the best performance in predicting the exosomes release profile of PAA/PPy-Hg hydrogel, while the n value of Korsmeyer-Peppas model was less than 0.5, further indicating that Fickian diffusion was the main release mechanism. Deng and co-workers used Higuchi model to fit the cumulative release profile of hypoxia preconditioned BMSC-derived EVs from an inectable bioactive hydrogel composed of PEG/polypeptide copolymers and found that the release curve of EVs had a good match with Higuchi model, with a correlation coefficient of R^2^ > 0.98, demonstrating the release was mainly dependent on a diffusion-controlled manner [384]. Wang and co-workers used Higuchi model to fit the release profiles of exosomes from both CS@Exo nanoparticle and PLA/PMLACS@Exo composite coating [385]. They found that both of them exhibited good match with Higuchi model, with the correlation coefficients of R^2^ = 0.9863 and R^2^ = 0.97095, respectively. Shiekh and co-workers used two models (Higuchi and Korsmeyer-Peppas models) to fit the cumulative EV release from ORC and BOOST scaffolds [386]. They found that Higuchi plots exhibited linearity (R^2^ > 0.95), indicative of a diffusion-controlled process; while the Korsmeyer-Peppas exponent (n) remained below 0.45 for both scaffolds (0.34 for ORC; 0.41 for BOOST), confirming Fickian diffusion as the dominant release mechanism. Additionally, BOOST displayed a lower apparent rate constant (k = 25.6) compared to ORC (k = 29.9), suggesting stronger interactions between EVs and the scaffold matrix.

#### 4.3.5 Korsmeyer-Peppas or Ritger-Peppas model

The Korsmeyer-Peppas model (proposed by Korsmeyer and Peppas in 1983), also known as the Ritger-Peppas model (introduced by Ritger and Peppas in 1987), characterizes the release mechanism from polymeric matrix systems, which may involve diffusion, swelling, or matrix erosion [387]. This model is also known as the modified power-law model, which indicates that there is an exponential relationship between the fraction of drug release and time [388]. equation (5) is as follows:

(5) $$f_{t}=k_{\text{kp}}\cdot t^{n}$$

where f_t_ represents the fraction of EVs released at the testing time; k_kp_ is a kinetic constant of the exosome-polymer system; and n is the diffusion or release exponent. The value of the release exponent (n) determines the drug release mechanism, which can be classfied into four regimes: (a) if n ≤ 0.45, the release follows Fickian diffusion, where the concentration gradient plays a decisive role; (b) if 0.45 < n < 0.89, the transport is anomalous (non-Fickian), where both diffusion and polymer swelling jointly regulate the release; (c) if 0.89 ≤ n < 1.0, the mechanism corresponds to Cast II transport or polymer erosion; in this range, the Korsmeyer-Peppas model indicates a release predominantly by diffusion; (d) if n = 1, the mechanism corresponds to the zero-order release, characterized by a constant release rate independent of time [[389], [390], [391]]. For instance, Zhou and co-workers utilized Korsmeyer-Peppas model to fit the hUMSC-derived exosomes release data from CuMOFs/CTMG/Exos composite sponge [392]. They found that a relatively high goodness of fit (R^2^ > 0.9) was achieved across all groups, and the release mechanism of exosomes varied with microenvironment conditions: pH value (neutral > acidic) and ROS (high > low) affect release by adjusting the rate constant (k) and the release exponent (n). In another study, Li and co-workers used Korsmeyer-Peppas model to fit the release profile of exosomes from SA@MED hydrogel, with a goodness-of-fit of R^2^ greater than 0.95 and the release exponent (n) of 0.7, suggesting that exosome release was govened by a comination of diffusion and polymer network swelling-relaxation mechanisms [393]. Margaronis and co-workers employed the Korsmeyer-Peppas model to fit the release kinetic of yogurt EVs from an injectable hydrogel. The fitted parameters yielded n = 0.61 and k = 0.15 (Fig. 13E), consistent with anomalous (non-Fickian) transport, suggesting that the release mechanism was governed not only by diffusion but also by matrix relaxation [373].

#### 4.3.6 Peppas-Sahlin model

The Peppas-Sahlin model is an extension of Korsmeyer-Peppas and Ritger-Peppas models [332]. equation (6) is as follows:

(6) $$f_{t}=k_{1}\cdot t^{m}+k_{2}\cdot t^{2m}$$

where f_t_ denotes the fraction of EVs released at the testing time; k_1_ corresponds to the release constant for Fickian diffusion, whereas k_2_ represents the kinetic release constant associated with Case II relaxation [381,394]; and m is the diffusional exponent related to Fick's diffusion (dimensionless). For instance, Cedillo-Servin and co-workers utilized four kinetic models (first-order, Higuchi, Ritger-Peppas and Peppas-Sahlin models) to investigate the EV release mechanisms involved in GelMA hydrogels [395]. Based on the comparison of the correlation coefficient (R^2^) and of the second-order Akaike Information Criterion (AIC_C_), they found that Peppas-Sahlin model showed the best fit (with the highest R^2^ and lowest AIC_C_ values) for the release data. Moreover, across all tested GelMA concentrations, the absolute value of the diffusion constant k_1_ exceeded that of the chain relaxation constant k_2_.

#### 4.3.7 Gallagher-Corrigan model

The Gallagher-Corrigan model is used for the release of EVs from degradable biopolymers [396]. The standard mechanisms used to evaluate EVs release from biodegradable polymeric delivery systems involve a combination of diffusion and degradation [397]. equation (7) is as follows:

(7) $$f_{t}=f_{B}\left(1-e^{{-k}_{1}t}\right)+\frac{\left(f_{t,\max}-f_{B}\right)\left(e^{k_{2}\cdot t}-k_{2}\cdot t_{m\text{ax}}\right)}{1+e^{k_{2}\cdot t}-k_{2}\cdot t_{m\text{ax}}}$$

where f_t_ is the fraction of EVs released at the testing time; f_B_ is the maximum fraction during the burst release phase; f_t,max_ is the maximum releasable content; t_max_ is the time required to reach the maximum release rate; and k_1_ and k_2_ are the first-order rate constant and sustained-release kinetic constant, respectively. For instance, Ashrafi and co-workers used two kinetic models (Korsmeyer-Peppas, Gallagher-Corrigan) to fit the exosome release experimental data from an alginate hydrogel composited with a PCL nanofibrous layer [398]. They found that Gallagher-Corrigan model was better fit the release kinetic process of hPMSC-derived exosomes, with a goodness of fit R^2^ of 0.9961 and a relatively high k_2_ value, underscoring the critical role of hydrogel degradability in mediating the release of exosomes. Subhan and co-workers fabricated a thermo-responsive hydrogel (Exo-Q) and observed a biphasic release profile: an initial burst release followed by a sustained release phase driven by gradual network relaxation and matrix erosion [157]. By fitting the cumulative release profile with the Gallagher-Corrigan model, as shown in Fig. 13F, they found that the rate constants k_2_ > k_1_, indicating that exosome release governed by matrix erosion processed more slowly than the initial diffusion-dominated phase.

### 4.4 Effect of biomaterial properties on EV release

The physicochemical characteristics of biomaterials serve as the fundamental regulatory determints for govering EV release kinetics, as well as cellular uptake and therapeutic efficiency. The physicochemical characteristics of biomaterials mainly include mechanical cues, topographical cues, internal porosity, surface charge, and degradation rate.

#### 4.4.1 Mechanical cues

A growing body of evidence indicates that mechanical cues, specifically matrix stiffness, matrix viscoelasticity, and mechanical stress, exert a significant influence on EV release kinetics and cellular mechanobiology.

The matrix stiffness can modulate the release kinetics and functional properties of EVs through mechano-transduction pathways and EV pre-conditioning effects. Specifically, matrix stiffness governs the mechanical microenvironment of parent cells, thereby regulating intracellular signaling cascades that control EV biogenesis and cargo sortin. Specifically, substrate stiffness markedly modulates the characteristics of EVs, and the size increases when the substrate stiffness is below 20 kPa whereas it decreases on stiffer substrates [399]. Moreover, compared with stiffer silica nanoparticles (Young's modulus: 1064.7 MPa), the softer silica nanoparticles (Young's modulus: 103.2 MPa) upregulated exosomal markers and promoted greater exosome release, and macrophages sense nanomechanical signals through Piezo1 regulate the release of exosomes [325]. Additionally, EVs from medium stiffness substrates, which mimic the mechanical microenvironment of healthy cartilage, enhance chondrocyte proliferation, attenuate inflammatory responses, and promote the phenotypic transition of macrophages from M1 toward the M2 subtype; while exosomes from soft stiffness substrates, indicative of osteoarthritic conditions, exacebrate chondrocytes inflammation and drive M1 polarization [400]. Furthermore, a relavant study indicates that moderate stiffness levels (100 Pa vs. 250 Pa) of materials do not exhibit statistically significant differences in EV release [401], but EVs derived from stiff matrices potently promoted cell proliferation compared to EVs derived from soft matrices [328]. Therefore, matrix stiffness serves as a critical biophysical cue that not only determines EV release kinetics and physical characteristics but also dictates their biological functions and influences cellular behavior.

The matrix viscoelasticity of biomaterials can regulate the secretion of EVs. For instance, compared with elastic microenvironment-derived exosomes (e-Exos), the local delivery of viscoelastic microenvironment-derived exosomes (v-Exos) via a thermosensitive PF127 hydrogel significantly enhanced diabetic wound healing through reducing inflammation (as reflected by the promoted phenotypic transition of macrophages from M1 to M2), and enhanced angiogenesis (including induced tube formation of endothelial cells as well as increased proliferation and migration of fibroblasts) [322]. Thus, matrix viscoelasticity serves as pivotal biophysical cues that direct EV-mediated intercellular communication, offering critical design parameters for functionally tailored biomaterials in wound healing.

The mechanical stress within biomaterials can enhance the pro-angiogenic activity via modulating the secretion of EVs. For instance, mechanical stress promotes the pro-angiogenic activity of fibroblast-derived exosomes through YAP-dependent enrichment of BMP2 and CXCL8, both of which serve as the key mediators within the VEGFA-VEGFR2 signaling axis [326]. MSCs respond to diverse mechanical cues, including substrate stiffness and fluid shear stress. Recent evidence demonstrates that exposure to additional mechano-regulatory parameter, physical confinement, enhances the pro-angiogenic bioactivity of MSC-derived EVs [402]. Mechanical stress within a scaffold promotes angiogenesis by regulating the secretion and function of fibroblast-derived exosomes [330]. Thus, mechanical forces orchestrate EV-mediated pro-angiogenic responses across multiple cell types, highlighting a central mechanobiological mechanism for vascular repair.

#### 4.4.2 Topographical cues

The topographical features of biomaterials (including micro/nano-scale roughness, fiber alignment, pore morphology, and patterned surface grooves) serve as critical physical regulatory cues that modulate the phenotypic polarization of parent cells, cytoskeletal arrangement, and fine-tuning endogenous EV biogenesis, secretion yield, and cargo functionalization, thereby enabling tailored EV release. For instance, Peng and co-workers reported a ferroelectric living interface for fine-tuning exosome secretion [403]. This system utilizes unique topographical and electrical (piezoelectric and photoelectric) signals to continuously generate bioactive exosomes through the rational construction of a ferroelectric layer of a polydopamine/poly(vinylidene fluoride-co-trifluoroethylene) (PDA/PVDF-TFE) film with nanoscopic grooves and a living cell layer (MSCs). Additionally, static topographical cue combined with dynamic fluid stimulation can enhance the yield of macrophage-derived EV [323]. Additionally, topography plays a central role in modulating cellular behavior [404]. Thus, the topological structure design of biomaterials represents a powerful approach that can guide the EV-based intercellular communication for regenerative medicine.

#### 4.4.3 Internal porosity

The internal porosity of scaffolds directly dictates EV release kinetics by regulating diffusion constraints, EV-matrix inetractions, and local microenvironmental conditions. Small pore size of materials enables continuous and sustained release of EVs over days to weeks. In contrast to the non-porous or small-pore systems, the void-forming materials (e.g., cryo-gel) offer two key advantages: one is the enhanced EV loading capacity via increased void volume, allowing for higher initial EV loading; and another is a high EV release ratio due to reduced diffusion resistance [405]. Importantly, these porous systems also improve therapeutic efficacy in the treatment of diabetic wound healing with faster-re-epithelialization, neovascularization, and collagen deposition. This is partially attributed to the ability of large pores to facilitate cell infiltration into the matrix, creating a pro-regenerative niche where EVs and host cells interact synergistically to accelerate tissue repair.

#### 4.4.4 Surface charge

Surface charge of biomaterials can modulate EV-matrix interactions, adsorption capacity, and subsequent cellular uptake through electrostatic interactions, which are critical for optimizing EV delivery efficiency. Polycationic electrolytes modified scaffolds (e.g., chitosan-containing scaffolds [406], PEI-modified surfaces [272]), MXene-contained hydrogel [49], or MOF-loaded scaffolds [261] can bind electrostatically to the negatively-charged EV membrane surface, achieving high adsorption capacity of EVs and reducing premature EV leakage. This electrostatic interaction also modulates EV release kinetics: positively-charged matrix retains EV more tightly, leading to sustained release, whereas negatively-charged or neutral matrixes exhibit faster initial EV release due to repulasive or non-interactive forces. Furthermore, the positively-charged matrix can enhance cellular uptake of release EVs by promoting electrotatic interactions between EVs and the negatively-charged cell membrane, thereby increasing the bioavailability of EV cargo. In constrat, negatively-charged materials may reduce EV-cell interactions, limiting cellular uptake efficiency and therapeutic potency.

#### 4.4.5 Degradation rate

Biomaterials that can be degraded through hydrolysis, collagenase, or hyaluronidase enzyme (such as gelatin [407], hyaluronic acid [408,409]) control the release of EVs. Additionally, stimulus-responsive biomaterials with stimulus-responsive polymers or sensitive chemical bonds (such as pH-responsive bonds [207], ROS-sensitive bonds [410], MMP-cleavable peptides [347]) allow on-demand release of EVs in the disease microenvironments. Degradation rate of biomaterials directly governs the release profile of EVs. Slow-degrading materials (such as high molecular weight polymers, highly-crosslinked hydrogels) provide continuous and sustained release of EVs over weeks to months, while fast-degrading materials (such as low molecular weight of polymers and peptides) exhibit early burst release of EVs [237]. Slow degradation aligns with chronic wounds requiring long-term EV signaling, whereas rapid degradation suits acute wounds where short-term high-dose EV exposure is desired. Mismatch between degradation rate and EV stability leads to loss of bioactivity.

Crucially, these physicochemical properties of biomaterials are not independent but exhibit complex interdependencies that collectively regulate EV delivery and therapeutic outcomes. For example, material porosity influences degradation rate: large pores increase matrix hydration, accelerate hydrolytic cleavage of polymer bonds, and enhance enzymatic infiltration, thereby speeding up degradation. Similarly, matrix stiffness is dynamically regulated during degradation (e.g., crosslinked hydrogels become softer as crosslinks are broken, which can feedback on EV release by altering matrix-EV interactions and mechano-transudction in infiltrating cells).

## 5 Wound healing accelerated by EV-loaded biomaterials

Clinically, skin injury is classified into two primary types: acute and chronic. Acute wounds typically follow a physiological healing cascade and achieve closure within four weeks, but can extend to twelve weeks at the certain conditions such as skin ulcers [411]. In contrast, chronic wounds [412,413], such as diabetic foot ulcers, burn injuries, infected wounds, and radiation-induced skin injuries, exhibit impaired healing characteristics (slow healing or nonhealing) due to the microenvironment dysregulation [414]. Current therapeutic modalities for wound management include dry dressings, surgical debridement, growth factors, stem cells, skin grafts and flaps, hyperbaric oxygen therapy (HBOT), negative pressure wound therapy (NPWT), and electrical stimulation (ES). A detailed comparative summary of these approaches is presented in Table S4. Recent advances in cell-free therapeutic strategies have highlighted the synergistic potential of EV-loaded biomaterial platforms for skin wound repair [415]. The following subsections will mainly focus on the recent progress of EV-loaded biomaterials for treating five clinically challenging wound subtypes: diabetic ulcers, infected trauma, burn damage, radiation-induced skin injuries, and compromised skin flaps.

### 5.1 Diabetic ulcers

According to the prediction of the International Diabetes Federation (IDF), approximately 578 million adults worldwide will have diabetes by 2030 [416]. Diabetic chronic ulcers (DCUs), especially diabetic foot ulcers (DFUs), are one of the most common clinical complications in diabetic patients, mainly caused by neuropathy and vascular changes resulting from persistent hyperglycemia [[417], [418], [419], [420], [421]]. Persistent hyperglycemia often leads to ulcers on the feet or lower limbs that are difficult to heal [422,423]. Diabetic wound healing brings various challenges, mainly including impaired angiogenesis, persistent infection, high inflammation, tissue hypoxia, excessive oxidative stress, and delayed re-epithelialization [[424], [425], [426], [427]].

As a key mediator for intercellular communication, EV presents high therapeutic potential in treating diabetic wounds [428]. Functionally, EVs accelerate diabetic wound healing through multiple pathways including: stimulating angiogenesis [60,64,429], regulating inflammation [430] ameliorating oxidative stress [431], relieving high glucose damage to cells such as HUVECs [101] and fibroblasts [432], promoting keratinocytes proliferation and migration [433], facilitating cell cycle progression [434], inhibiting fibrosis [435], and suppressing ferroptosis [65]. Moreover, to address the inherent shortcomings of native EVs (like rapid clearance and short bioactivity duration), many advanced EV-loaded biomaterials have been developed for diabetic wound treatment.

**Fig. 14:**
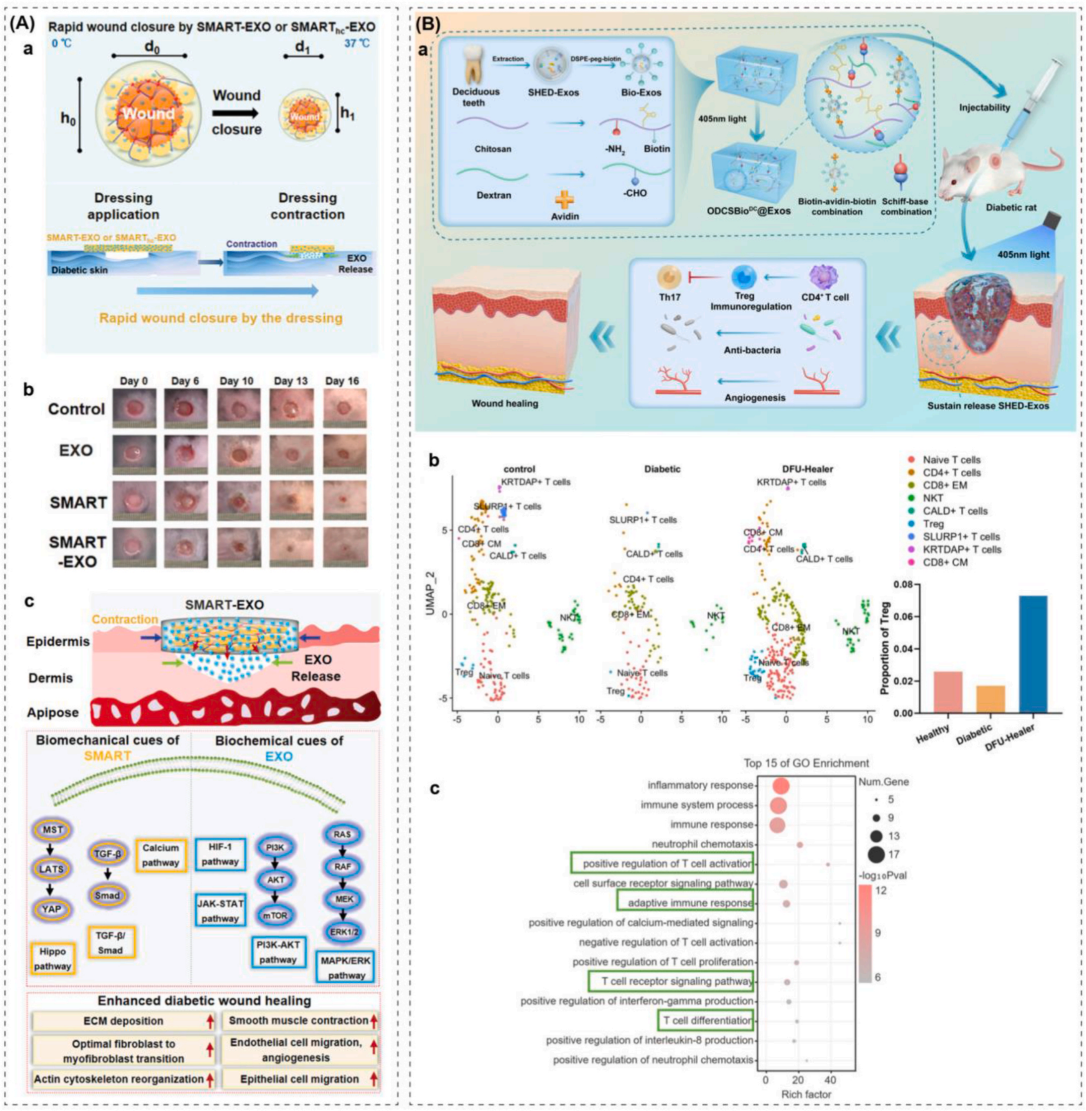
**Representative examples of EV-loaded biomaterials for diabetic wound treatment**. (A) (a) Schematic diagram of SMART-EXO or SMART_hc_-EXO promoting wound healing by applying wound traction and contraction-induced exosome release, (b) representative images of wound treated with SMART and SMART-EXO, (c) wound healing mechanism by SMART-EXO. Reproduced with permission [339]. Copyright 2024, Wiley-VCH. (B) (a) Schematic illustration of a bioinspired biotin-avidin hydrogel with prolonged SHED Exo delivery enhancing diabetic wound healing by modulating Treg/Th17 balance, (b) single-cell sequencing analysis of T cells, and (c) GO enrichment of upregulated genes in skin tissue. Reproduced with permission [437]. Copyright 2025, Elsevier B.V.

In recent years, researchers have been dedicated to by integrating EVs with other promising therapeutic modalities to address the multiple pathological issues of diabetic ulcers, such as oxidative stress, persistent inflammation, bacterial infections, and impaired angiogenesis. For instance, Zeng and co-workers developed CeO_2_ NPs-engineered exosomes and they found that the fabricated CeO_2_@Exo treatment promoted diabetic wound healing through anti-inflammatory and pro-angiogenic effects [436]. Xie and co-workers developed a SMART-EXO dressing by integrating MSC-derived exosomes into a self-contraction core-shell microgel asembly [339], as presented in Fig. 14A. They found that the triggering contraction property of SMART-EXO not only facilitated the efficient encapsulation of exosomes but also enabled their on-demand release. This dressing promoted diabetic wound healing through regulating both mechanical and biochemical regulation of multiple repair process, including cell migration, prolfieration, angiogenesis, and re-epithelialization. Liu and co-workers fabricated a sprayable Exo@AMCN hydrogel by loading hUCMSCs-derived exosomes and β-cyclodextrin bornel inclusion complexes into methacrylated decellularized dermal matrix [438]. The Exo@AMCN presented good biocompatibility, broad-spectrum antimicrobial property, antioxidant activity, as well as the ability to promote cell migration, angiogenesis, and M2 macrophage polarization. Exo@AMCN made the wound almost completely healed, attributed to the multi-mechanistic synergy of the composite hydrogel, including inflammation modulation, enhanced collagen deposition, and stimulation of neovascularization. Transcriptomic analysis further revealed that Exo@AMCN upregulated genes associated with angiogenesis and inflammation in recipient cells and activated key pathways such as PI3K/Akt. These findings indicate that the integration of EVs with other therapeutic components holds great potential in wound healing by addressing multiple pathological issues of diabetic ulcers.

Recent studies have shown that miRNA therapy holds great potential in the field of wound healing. As key regulators of cellular physiology and pathology, miRNAs can exert their effects by regulating the expression of genes related to wound healing [439]. However, the effective delivery of miRNAs is the key point of the therapeutics. miRNAs can be released by exosomes and these exosomal miRNAs can be taken up by neighboring or distant cells, influencing their functionality [440]. For instance, Wang and co-workers developed an injectable Gel-sEV-486-5p hydrogel by encapsulating miR-486-5p-modified sEVs into GelMA, and they found that the hydrogel promoted wound healing by enhancing angiogenesis via Smurf2/TGF-β1 pathway [441]. Fan and co-workers developed a dECM hdyrogel loading exosomes from hypoxic urine-derived stem cell and they found that this composite hydrogel facilitated diabetic wound healing by targeting SERPINE1 through miR-486-5p [442]. Shi and co-workers developed a multifunctional hydrogel (CG-OHA@Exo) and they found that CG-OHA@Exo accelerated diabetic wound healing by relieving macrophage dysfunction [443]. The underlying molecular mechanism may be that miR-4546-5p derived from hyBMSC-Exos and antioxidant property of the hydrogel synergistically inhibit SREBP2 activity. These findings indicate that it is possible to cure diabetic ulcers by using biomaterial-assisted miRNA-enriched EVs to deliver RNA and achieve precise targeting of essential cellular processes and molecular pathways.

Regulatory T cells (Tregs) have become a promising target for the treatment of diabetic ulcers by regulating immune system and supporting tissue repair [444]. In a recent study, by regulating Treg/Th17 balance in diabetic wounds, an injectable chitosan/dextran-based hydrogel (ODCSBio^DC^@Exos) was fabricated by Guo and co-workers [437], as showed in Fig. 14B. They found that this hydrogel promoted wound closure through facilitating angiogenesis, alleviating inflammation, and restoring Treg/Th17 balance. Transcriptomic analysis further demonstrated that the hydrogel regulated immune-related pathways, including positive regulation of T cell activation, immune response, and T cell differentiation, thereby reversing immune dysregulation in diabetic wounds. This system presents a promising strategy in treating diabetic wounds and may serve as a highly prospective candidate for clinical wound management.

### 5.2 Infected trauma

Bacterial infections bring a threat to global human health and drive the rapid development of antimicrobial agents. The clinical application of antimicrobial agents is greatly limited by the rapid occurrence of drug-resistant bacteria and biofilms [445]. Infected wounds often experience delayed healing or persistent non-healing [446]. The current treatment of infected wounds is confronted with a dual predicament: one is that the antibiotic resistance makes it difficult to eliminate pathogens,and the other is that the persistent inflammation leads to the impaired tissue regeneration.

Adding antibiotics to EV-loaded biomaterials is a commonly used strategy to protect wounds from bacterial infection. For instance, Dai and co-workers proposed a strategy amined at complex microenvironments, leveraging antibiotic-loaded platelet-derived extracellular vesicles (PEVs) for the repair of infected wounds [447]. Their findings revealed that incorporating entamicin-loaded PEVs into a hydrogel matrix not only augmented the targeted effect and bacterial killing efficiency but also facilitated macrophage recruitment for biofilm removal and promoted angiogenesis, thereby accelerating the healing process of infected diabetic wounds. In another study, three kinds of chiotosan-based self-healing hydrogels (ec⊂OH, ec⊂OHD, ec⊂OHPB) were developed by Li and co-workers [448]. Due to the incorporation of ciprofloxacin hydrochloride (CIP), these three hydrogels all showed antibacterial property against both E. coli and S. aureus, but ec⊂OHPB exhibited the strongest antibacterial activity. Importantly, ec⊂OHPB achieved long-term bactericidial effect due to the self-healing property of hdyrogel. In a S. aureus-infected stretchable wound model, ec⊂OHPB significantly accelerated wound healing by suppressing bacterial growth, alleviating inflammation, enhancing angiogenesis, improving collagen deposition, and facilitating epidermal regeneration. Together, the instantaneous self-healing behavior and superior drug rention capacity of ec⊂OHPB broaden the clinical application of chitosan-based wound dressings for infected stretchable wounds.

**Fig. 15:**
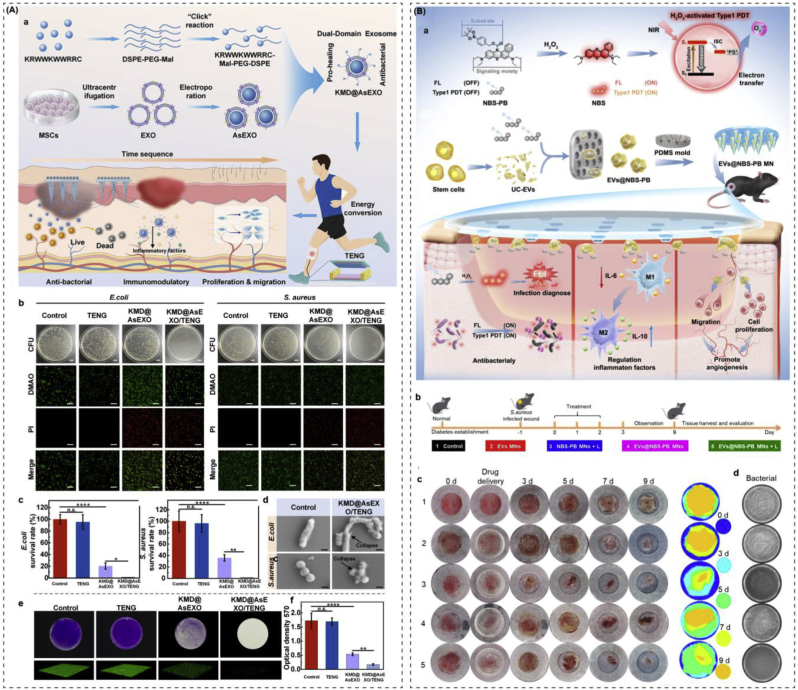
**Representative examples of EV-loaded biomaterials for infected trauma treatment**. (A) (a) Schematic illustration of the preparation of a self-powered KMD@AsEXO/TENG microneedle delivery platform for the treatment of infected wounds, (b) bacterial colony count and live/dead bacterial staining after treatment, (c) quantitative analysis of bacterial survival rates, (d) SEM images showing morphological changes in bacteria after treatment, (e) biofilm staining and fluoresence imaging, and (f) quantification of biofilm biomass based on optical density measurements. Reproduced with permission [454]. Copyright 2025, Elsevier Ltd. (B) (a) Schematic illustration of bacterial-responsive EVs@NBS-PB MNs for synergetic oxygen-independent phototheranostics and microenvironment regulation in hypoxia diabetic wounds, (b) schematic of the experimental protecol of in vivo infected motion site wound healing, (c) typical wound images and stimulated in vivo wound closure traces under different treatments, and (d) images of S. aureus colonies from the wound tissues. Reproduced with permission [455]. Copyright 2025, Elsevier B.V.

However, the abuse of antibiotics can easily lead to the emergence of drug-resistant bacteria, posing a great challenge to the treatment of infectious wounds. To address this issue, some antibiotic-free antibacterial agents, such as antimicrobial compounds (e.g., quaternized chitosan [449,450], berberine [451]) or bactericidal nanoagents (e.g., MXene [49], carbon dots [58], Ag NPs [452]), have been reported and combined with EV-loaded biomaterials for treating infected wounds. Moreover, antimicrobial peptides (AMPs) show great potential in preventing bacterial infections in wounds due to their broad-spectrum antibacterial activity and extremely low bacterial resistance [453]. Based on this, researchers attempted to introduce AMPs into EV-functionalized biomaterials to combat bacterial infections. For instance, Zhang and co-workers developed a self-powered microneedle delivery platform integrated with dual-domain engineered exosomes, denoted as KMD@AsEXO/TENG, for the treatment of infected wounds [454]. This platform integrated a triboelectric nanogenerator (TENG) that can precisely and effectively release exosomes through electrical stimulation, as shown in Fig. 15A. Owing to the incorproation of antimicrobial peptide (TET213), this platform demonstrated potent antibacterial activity, effectively eliminating planktonic bacteria and markedly suppressing biofilm formation, contributing to healing infected wounds. These findings highlight the great potential of the combined treatment with AMPs, EVs and biomaterials for infected wounds.

Tissue hypoxia often limits the efficacy of conventional antimicrobial photodynamic therapy (aPDT). To address this issue, Zeng and co-workers developed an O_2_-independent phototheranostic system based on hUCMSC-derived EVs engineeredby a type I pro-photosensitizer NBS-PB, denoted as EVs@NBS-PB [455], as illustrated in Fig. 15B. They found that NBS-PB exhibited specific responsiveness to the infected wound mciroenvironment, allowing accurate assessment of infection severity and enabling targeted type I-photodynamic antibacterial activity. In S. aureus-infected diabetic wound models, EVs@NBS-PB with laser irradiation demonstrated great potential in fluorescence imaging-guided targeted antibacterial therapy, regulating wound microenvironment, promoting angiogenesis, and accelerating wound closure. These findings highlight a simple and intelligent phototheranostic paltform achieved by simply incorporating a single photosensitizer molecule into biocompatible stem cell vesicles, with diagnosis, disinfection and microenvironment improvement effects to synergistically accelerate diabetic wound healing.

### 5.3 Burn damage

Burns are traumatic injuries characterized by skin and/or subcutaneous tissue damage caused by thermal (such as flame, hot water, or steam), chemical, electrical, radiative or frictional agents [[456], [457], [458]]. Clinically, based on injury depth, burns are classified into three degrees: first-degree burns (affecting only the epidermis), second-degree burns (involving the dermis), and third-degree burns (extending to the full-thickness skin layer) [459]. The pathophysiology of burn wound healing is a highly complex and dynamic continuum, and classically divided into three overlapping stages: (i) hemostasis/inflammation, (ii) proliferation, and (iii) remodeling [460]. The disruptions in any phase can lead to complications such as chronic wounds or excessive scarring.

Emerging as a promising cell-free therapeutic approach, EV-based therapies hold significant potential for burn wound healing [461]. EVs exert multifaceted therapeutic effects: (i) regulating excessive inflammatory responses [135], (ii) suppressing heat-stress-induced apoptosis [462], (iii) facilitating cell proliferation and migration [84], (iv) promoting angiogenesis [463], and (v) reducing scar formation [464]. Notably, EVs may also facilitate skin appendage regeneration, including hair follicles [465,466] and sweat glands [39]. which are critical for long-term function recovery. Furthermore, integrating EVs with biomaterials offers synergistic advantages by prolonging EVs retention at wound sites and enabling sustained release to amplify therapeutic efficacy [467].

**Fig. 16:**
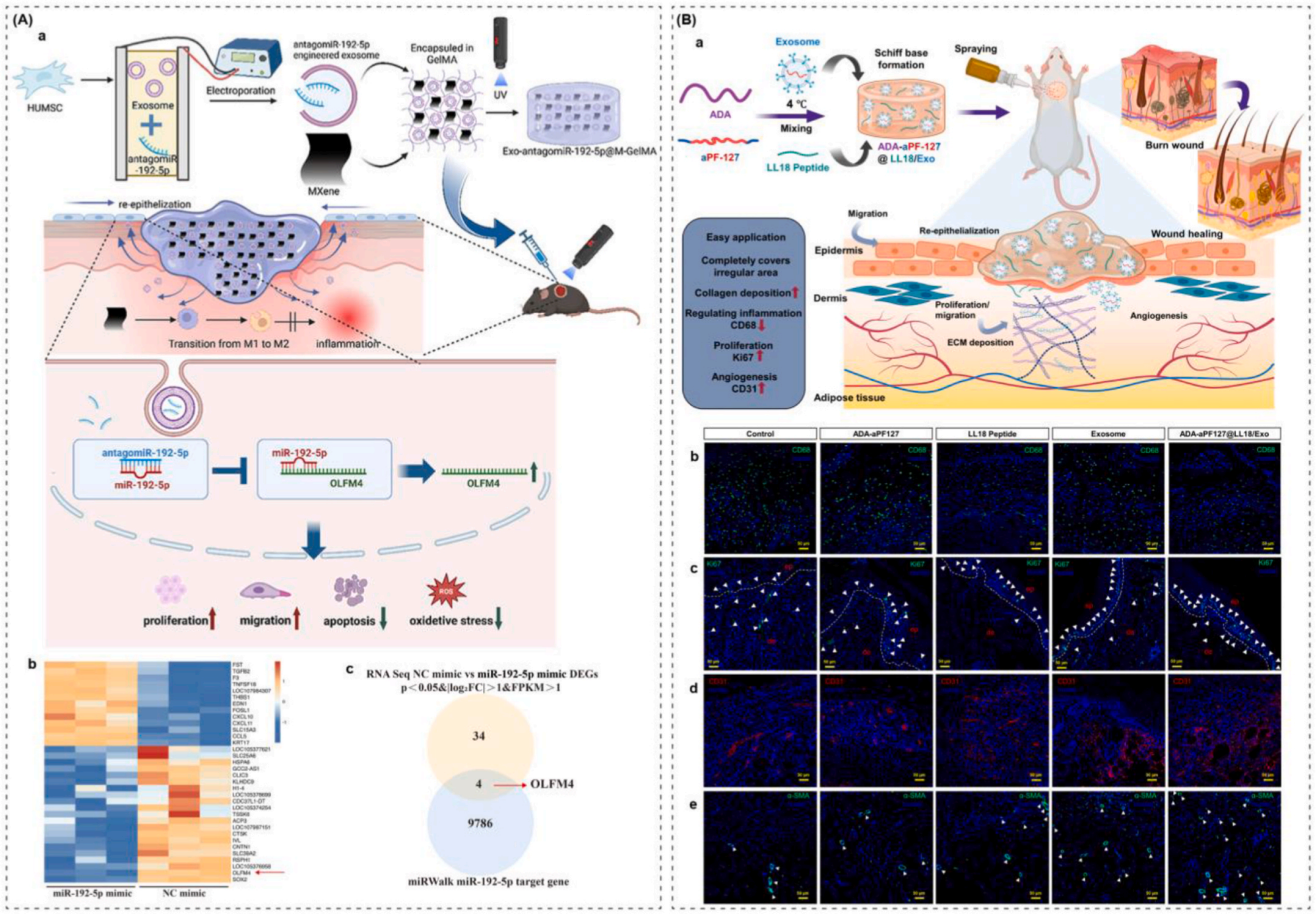
**Representative examples of EV-loaded biomaterials for burn wound treatment**. (A) (a) Schematic illustration of the fabrication of Exo-ant-192@M-Gel hydrogel and mechanism in healing burn wounds, (b) heatmap of differentially expressed genes, and (c) Venn diagram showing the intersection of differentially expressed genes and predicted miR-192-5p target genes from the miRWalk database. Reproduced with permission [468]. Copyright 2025, Elsevier B.V. (B) (a) Schematic illustration of the fabrication and application of ADA-aPF127@LL18/Exo for burn wound healing, representative immunofluorescence staining images for (b) CD68, (c) Ki67, and (d) CD 31 and (e) α-SMA. Reproduced with permission [69]. Copyright 2024, Elsevier B.V.

To identify the key miRNAs governing epithelization of burn wounds, Liu and co-workers used high-throughput sequencingto reveal a significant upregulation of miR-192-5p in HaCaT keratinocytes under oxidative stress and burn wound tissues [468], as illustrated in Fig. 16A. They found that inhibiting miR-192-5p enhanced the function of epidermal cells by upregulating olfactomedin-4 (OLFM4). To enhance the delivery and therapeutic effect of miR-192-5p inhibitors, they engineered exosomes with antagomiR-192-5p (ant-192), and then loaded them in a composite hydrogel comprising GelMA and MXene, to form the final Exo-ant-192@M-Gel. In a full-thickness burn wound model, Exo-ant-192@M-Gel presented anti-inflammation and antioxidant properties, along with a pro-healing effect. This hydrogel achieved nearly complete wound closure. Collectively, these findings demonstrate a novel drug-loading platform that effectively promotes burn wound repair by targeting miR-192-5p/OLFM4 axis.

Severe burns can cause neurovascular network destruction, uncontrolled inflammation and insufficient angiogenesis, leading to incomplete healing, abnormal collagen deposition, and poor tissue regeneration. To resolve these issues, Chai and co-workers prepared an organic-inorganic nanocomposite hydrogel (Gel-PL/MMF/EXO) by targeting neurovascular network reconstruction for burn wound repair [469]. They found that the hydrogel significantly accelerated wound closure with a high closure rate, boosted collagen deposition, and promoted angiogenesis and innervation. Additionally, this hydrogel also enabled scarless healing (epidermal thickness matching native skin) and promoted the regeneration of skin appendages. Elakkawi and co-workers developed HAMA microneedles loaded with exosomes derived from 3D-cultured hUCMSCs and found that EXO@HAMA accelerated the closure of deep second-degree burn wounds, promoted collagen maturation, reduced inflammation, promoted collagen maturation, upregulated the expression of CD31 and COL-1, and downregulated the expression of CD68 and β-catenin [470]. Transcriptomic analysis further revealed that molecular reprogramming-including downregulation of CTSB/ITGAD and activation of Wnt/β-catenin signaling and arachidonic acid metabolism-was associated with ehanced collagen deposition, epithelialization, and reduction in CD68^+^ macrophage infiltration. These findings demonstrate the synergistic effect of biomaterials and EVs in the repair of burn wounds and the regeneration and recovery of functional tissues.

Even if deep burns heal, they may leave unsatifactory scars, seriously affecting the quality of life of patients. To achieve scarless healing of deeply infected burn wounds, Yang and colleagues developed a rapid freeze-dry-thaw macro-porous hydrogel (HD-DP7/Exo) and found that it could be stored for a long time or transported over long distances after freeze-drying [471]. Importantly, this hydrogel accelerated the closure of deep second-degree burn wounds infected by Pseudomonas aeruginosa (PAO1), and dramatically decreased PAO1 colony counts, denser collagen arrangement, wider new collagen, as well as more skin appendages regeneration. Vipin and co-workers reported a sprayable thermal-sensitive polysaccharide-based hydrogel, loaded with antimicrobial peptide LL18 and ADMSC-derived exosomes [69], as illustrated in Fig. 16B. This hydrogel demonstrated excellent therapeutic effects on deep partial-thickness burns, including enhanced re-epithelialization, robust formation of granulation tissue, increased collagen deposition, reduced inflammatory responses, and facilitated angiogenesis, as well as promoting the regeneration of hair follicles. These findings provide highly promising dressings for the treatment of deep burnsand their multiple biological properties contribute to high-quality repair of burn wounds.

### 5.4 Radiation-induced skin injuries

Radiation-induced skin injury (RISI) is a common complication affecting about 95% of patients receiving radiotherapy and is caused by a different pathological mechanism compared to conventional burns or ulcers [[472], [473], [474]]. The mechanism of RISI is relatively complex, primarily including (i) DNA damage, (ii) oxidative stress, (iii) inflammatory response, and (iv) tissue fibrosis [475], which collectively exacerbate tissue damage and hinder healing [[476], [477], [478], [479]]. Current clinical management of RISI predominantly relies on conventional approaches including surgical debridement, wound dressing replacement, and surgical interventions [480]. However, these existing therapies exhibit substantial clinical limitation, being principally confined to non-specific interventions, such as physical debridement and hyperbaric oxygenation therapy [481], with few targeted therapeutic products available commercially.

**Fig. 17:**
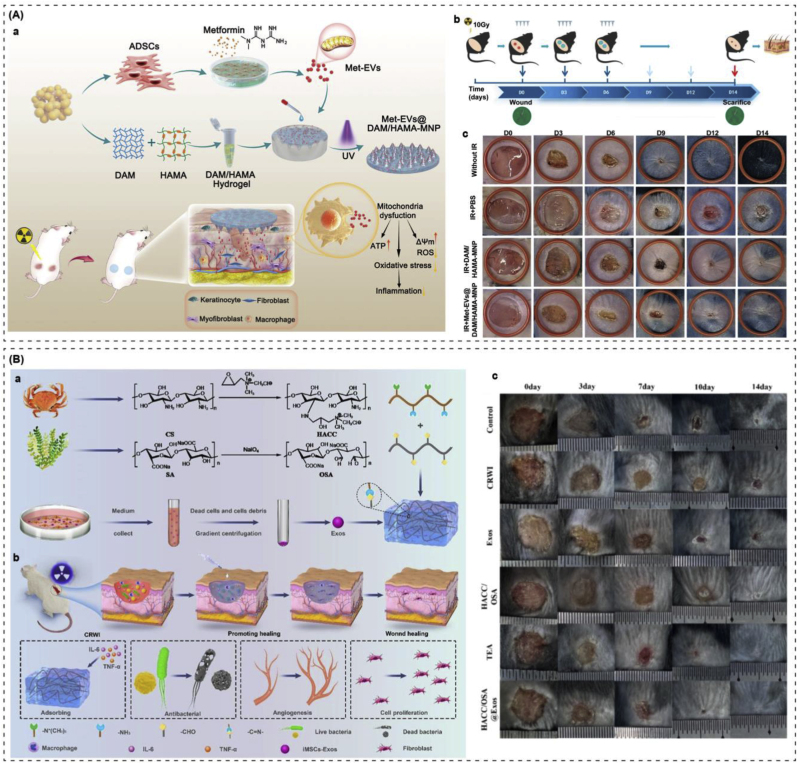
**Representative examples of EV-loaded biomaterials for treating radiation-induced skin injuries**. (A) (a) Schematic illustration of the fabrication of Met-EVs@DAM/HAMA-MNP and treatment in radiation combined with skin wounds, (b) schematic of radiation combined with skin wound model and treatment strategy, (c) photographs of wounds. Reproduced with permission [259]. Copyright 2024, American Chemical Society. (B) (a) Schematic diagram of the preparation of HACC/OSA@Exos hydrogel, (b) mechanism of action, and (c) images of wounds in different groups. Reproduced with permission [450]. Copyright 2024, Elsevier B.V.

Mitochondrial rescue strategies can restrain the production of mitochondrial ROS and eliminate oxidative stress [482,483], providing a promising approach for addressing chronic radiation injuries. For instance, as illustrated in Fig. 17A, Yao and co-workers developed a microneedle patch (MNP) loading mitochondria-enriched EVs derived from Met-treated ADSCs (Met-EVs) within a decellularized adipose matrix (DAM) and HAMA hydrogel, denoted as Met-EVs@DAM/HAMA-MNP [259]. In a radiation-impaired dorsal skin wound model, MNP treatment significantly promoted wound closure and exhibited greater epithelial thickness and higher COL-1 deposition, as well as promoting the expression of vascularization markers CD31 and α-SMA expression. Moreover, MNP treatment effectively ameliorated metabolic disorders by upregulating ATP concentration and downregulating ROS level. Furthermore, the MNP facilitated phenotype conversion of macrophages from the M1 subtype to the M2 subtype in wound tissue, along with decreased levels of pro-inflammatory factors (including IL-6, IL-1β, and TNF-α) and increased levels of anti-inflammatory factors (including Arg1 and IL-10). This study provides an effective EV delivery system with considerable clinical potential for the future clinical management of raidation-induced chronic wounds.

Several studies have shown that MSC-EVs can accelerate the healing process of radiation-induced injury [484]. Additionally, integrating EVs with biomaterials enhances their therapeutic efficacy by addressing the multifaceted pathophysiology of RISI. For instance, Fang and colleagues reported a multifunctional hydrogel, PDA-NPs@MSC-sEV, combining polydopamine nanoparticles (PDA NPs) and MSC-sEV within an OHA/carbohydrazide-modified gelatin (Gel-CDH) matrix [485]. They found that the hydrogel effectively scavenged ROS, alleviated oxidative stress and inflammation, and enhanced cellular proliferation and migration. In vivo, the hydrogel improved the repair process of radiation-combined skin wound by facilitating re-epithelization and skin tissue reconstruction. Proteomic analysis showed the hydrogel improved tissue repair and regeneration bymodulating wound microenvironment. To address the clinical challenge of treating combined radiation-wound injury (CRWI), characterized by delayed healing and high infection risk, Peng and co-workers developed an injectable exosome-loaded hydrogel integrating quaternized chitosan (HACC), oxidized sodium alginate (OSA) with exosomes derived from iMSCs [450], as illustrated in Fig. 17B. The fabricated HACC/OSA@Exos presented self-healing, bioadhesive, antibacterial and biocompatibile properties. It enabled sustained exosome release while preserving exosome integrity. In vitro, the hydrogel facilitated the cell proliferation, migration and angiogenesis. In vivo, it facilitated CRWI repair by accelerating re-epithelialization, collagen deposition, and neovascularization. These findings suggest that EV-loaded biomaterials hold great potential in promoting the regeneration of damaged tissues after radiation-induced skin injury.

### 5.5 Compromised skin flaps

Skin flap surgery is commonly employed in plastic surgery for healing tissue defects caused by diverse factors, such as post-tumor excision wounds, congenital deformities, chronic vascular ulcers, or diabetes complications [[486], [487], [488]]. However, flap viability is still affected by some challenges, such as limited length-to-width ratios, insufficient blood supply, and ischemia/reperfusion (I/R) injury, which may collectively lead to transplantation failure [489]. Current intervention methods, including the use of angiogenic drugs, growth factor administration, stem cell therapy, and anti-inflammatory/oxidatant intervention, have all demonstrated the effectiveness of improving flap survival [490]. Their clinical translation is hindered by inherent limitations like high doses, short half-lives, risks of cell contamination, and potential tumorigenicity [491].

EVs have received extensive attention in improving flap survival, due to their potential to promote angiogenesis [145], activate autophagy [492], and reduce oxidative stress damage [493]. Researchers have demonstrated that the combined treatment of EVs and biomaterials not only enhances EV biostability but also enables the improved survival of flaps. For instance, Yang and co-workers encapsulated human adipose-derived stem cell-derived exosomes (hASC-Exos) into a PF127 hydrogel, demonstrating that this construct enhanced the survival of autologous fat grafts via HIF-1α-mediated angiogenic potentiation [494]. Wang and co-workers developed a tannic acid (TA)-based hydrogel loaded with VEGF mRNA-engineered EVs, and found that the resulting VEGF-EVs@TA-Gel system promoted the viability and quality of prefabricated flaps by enabling sustained release of VEGF mRNA-loaded EVs [495]. Liu and co-workers developed a sprayable hydrogel containing sEV derived from platelets (PL-sEV) [496]. They found that PL-sEV enhanced skin flap survival by boosting blood perfusion, inhibiting PANoptosis via suppressing the NF-kB pathway, and rescuing endothelial cell dysfunction induced by oxygen-glucose deprivation/reoxygenation.

**Fig. 18:**
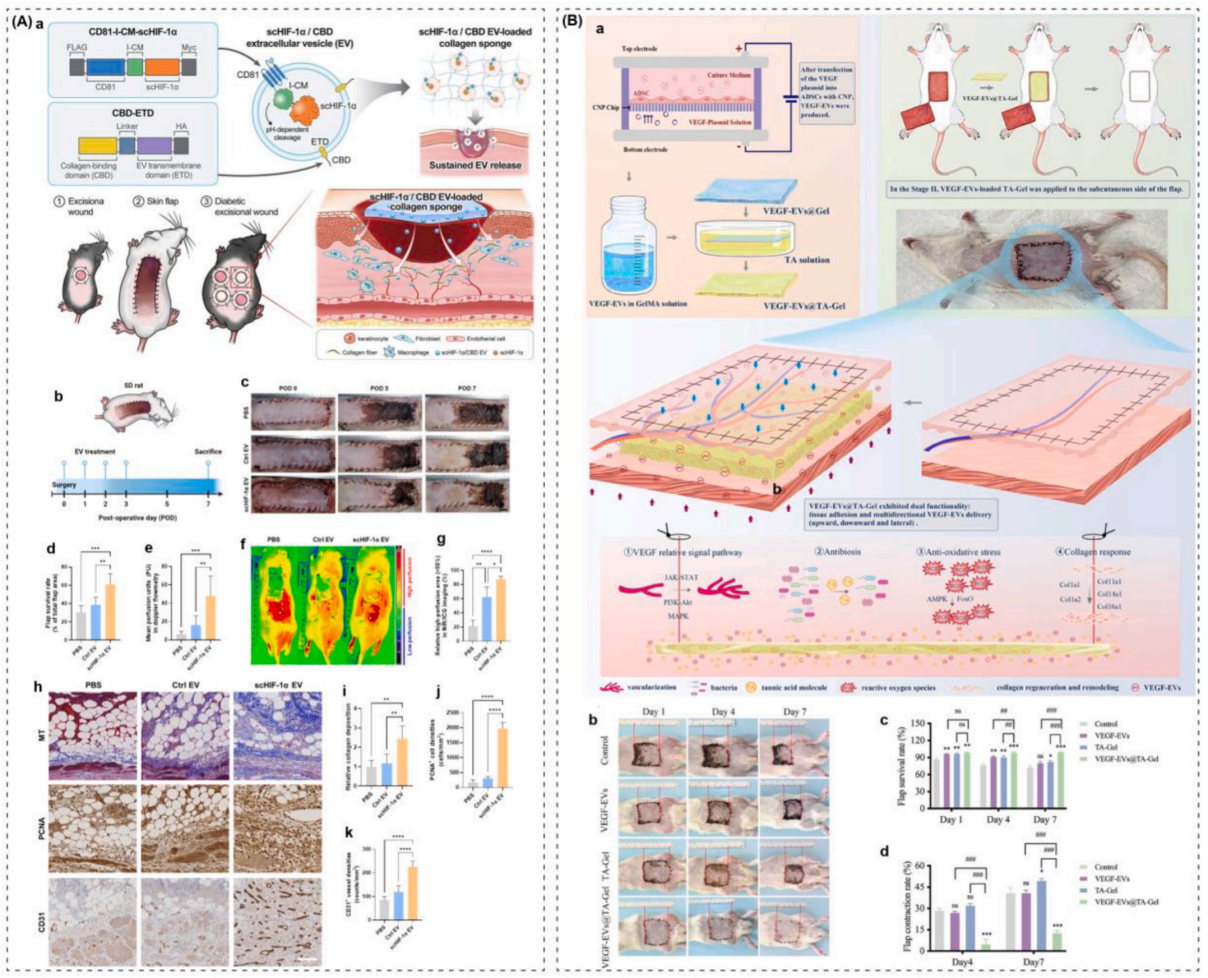
Representative examples of EV-loaded dressings for enhancing skin flap survival. (A) (a) Schematic illustration of the integration of EVs into CBD for enhancing various wound healing, (b) schematic of expermental timeline of scHIF-1α EVs in random-pattern skin flap model, (c) representative images of skin flap survival, (d) quantification of the skin flap survival rate, (e) mean perfusion units measured via Doppler flowmetry, (f) representative images of NIR imaging with (g) quantification of high-perfusion area, (h) MT and IHC staining images of flap tissue for PCNA and CD31, (i) quantification of relative collagen deposition, (j) PCNA^+^ cell densities, and (k) CD31^+^ vessel densities. Reproduced with permission [497]. Copyright 2025, Elsevier B.V. (B) (a) Schematic illustration of VEGF-EVs@TA-Gel preparation and the therapeutic effects in rat prefabricated flap regeneration and angiogenesis, (b) representative images of flap conditions and (c) statistical comparison of flap survival rates across different groups, (d) statistical comparison of flap contraction rats across different groups. Reproduced with permission [495]. Copyright 2025, Wiley-VCH.

Some researchers are dedicated to the combined treatment of EVs and biomaterials, aming to enhance the flap survival rate by promoting angiogenesis. For instance, to address the insufficient HIF-1α activation in chronic wounds, Jeon and co-workers loaded a stable, constitutively active form of HIF-1α into EVs, and found that the formed scHIF-1α EVs improved the viability of ischemic flaps by facilitating vascularization and tissue remodeling [497], as illustrated in Fig. 18A. Moreover, the addition of collagen-binding domain (CBD) further improved EV retention and amplified the therapeutic effect on angiogenesis. Sun and co-workers developed an engineered exosome (M-MS@EXO) and found that M-MS@EXO upregulated VEGF signaling pathway while downregulating TNF signaling pathways [498]. Local administration of M-MS@EXO not only significantly mitigated inflammatory responses but also promoted neovascularization in the Choke zone, providing a promising treatment strategy for promoting the survival of multi-territory perforator flap in diabetes. Wang and co-workers constructed a VEGF-EVs@TA-Gel hydrogel by integrating VEGF mRNA-loaded EVs (VEGF-EVs) into a TA-based hydrogel to enhance prefabricated flap survival [495], as illustrated in Fig. 18B. The VEGF-EVs@TA-Gel can enhance angiogenic activity and significantly increase the survival quality of prefabricated flaps. Moreover, this hydrogel upregulated differentially expressed genes related to VEGF-mediated pro-angiogenic pathways, collagen remodeling, and anti-oxidatant effect. These findings highlight the great potential of combining EVs and biomaterials to promote angiogenesis for enhancing flap survival.

## 6 Translational hurdles for EV-loaded biomaterials

Although the aforementioned preclinical studies have yielded encouraging results supporting the efficacy and safety of biomaterial carrying EVs in wound healing [499], the current preclinical literature generally suffers from low reproducibility. This limitation arises primarily from inadequate reporting of critical experimental variables, intrinsic biological heterogeneity of EVs, and inconsistencies in animal models (see Tables S1 and S2). Despite the overall promise of this therapeutic strategy, most available findings should be interpreted as exploratory rather than confirmatory. The clinical translation of biomaterials carrying EVs encounters a distinct set of challenges, including compliance with current good manufacturing practice (cGMP) for scalable production, reproducibility, long-term storage stability, regulatory considerations, and potential immunogenicity. The following subsections will discuss in detail these translational hurdles.

### 6.1 cGMP-compliant scalable production strategies

Advances in fundamental research have propelled the ongoing development of clinical investigations involving EVs. Table 2 provides a comprehensive summary of clinical trials registered on ClinicalTrials.gov as of April 2026 that evaluate EV-based therapeutics for various skin wounds. Similarly, Table 3 details clinical studies registered on the Chinese Clinical Trial Registry (ChiCTR) as of April 2026 that examine EV-based therapeutics for wound healing. Collectively, these trials underscore the substantial translational potential of EV-based therapeutics in the context of cutaneous wounds. However, in the translation of EV-loaded biomaterial platforms from laboratory research to clinical application, the establishment of a reliable and scalable production process is essential to ensure product stability and meet regulatory standards. Traditional methods for EV isolation, particularly ultracentrifugation (UC), exhibit considerable limitations in scalability due to volume restrictions and batch-to-batch variability [500]. These challenges have driven the exploration of alternative strategies more suitable for industrial-scale manufacturing.

**Table 2:** A comprehensive overview of clinical trials investigating EV-based therapeutics for a range of skin wounds (as of April 2026) by conducting a key word search for “exosome” or “extracellular vesicle” on ClinicalTrials.gov website.

Targeted skin diseases	ClinicalTrials.gov identifier	Official Title	Source of EVs	Purpose	Sponsor (Nation)	Last Update Posted
Cutaneous wounds	NCT06253975	A Randomized, Controlled, Multicenter Study of Human Adipose Tissue Derived Extracellular Vesicles Promoting Wound Healing	Human Adipose Tissue	To evaluate whether adipose tissue-derived EVs can promote effective healing of recalcitrant wounds.	Shanghai Ninth People's Hospital Affiliated to Shanghai Jiao Tong University (China)	2024-02-12
	NCT05475418	Pilot Study of Human Adipose Tissue Derived Exosomes Promoting Wound Healing	Human Adipose Tissue	By mixing EVs with sterile hydrogel and applied directly to the wound surface.	Shanghai Ninth People's Hospital Affiliated to Shanghai Jiao Tong University (China)	2023-10-19
	NCT04652531	Autologous Serum-derived Extracellular Vesicles to Treat Venous Trophic Lesions Not Responsive to Conventional Treatments	Autologous serum	To identify an alternative therapy to treat venous trophic lesions not responding to traditional therapeutic approaches using extracellular vesicles obtained from autologous serum.	University of Turin (Italy)	2023-05-12
	NCT02565264	Effect of Plasma Derived Exosomes on Cutaneous Wound healing	Plasma	To evaluate the effect of autologous exosomes rich plasma on cutaneous wound healing.	Kumamoto University (Japan)	2020-09-09
Diabetic Foot Ulcers	NCT06812637	Efficacy and Safety of Wharton's Jelly-Derived Mesenchymal Stem Cell Exosomes in the Treatment of Diabetic Foot Ulcers: a Randomized Controlled Trial	WJ-MSCs	To investigate the efficacy and safety of the topical application of WJ-MSC-derived exosomes in patients with chronic DFUs.	Kafrelsheikh University (Abdallah Mohammed Hafez)	2025-04-29
	NCT06825884	Use of Extracellular Vesicles (EV) for Diabetic Foot Ulcers	Autologous human platelet granules	To determine the short-term speed and effectiveness of extracellular micro vesicles (EV) on the healing of diabetic foot ulceration and prove that EV will significantly shorten the required time to heal DFU.	University of Jordan (Jordan)	2025-04-16
	NCT06319287	A Phase 2a Multi-Center, Prospective, Randomized Controlled Study to Evaluate the Safety and Efficacy of Topically Applied PEP-TISSEEL in Subjects with Diabetic Foot Ulcers (DFU)	Purified Exosome Product (PEP)	To evaluate the safety and efficacy of PEP-TISSEEL + Standard of Care (SOC) compared to SOC only treatment in 40 subjects, specifically for the treatment of their non-healing DFU.	Rion Inc. (United States)	2024-11-15
	NCT05243368	Evaluation of Personalized Nutritional Intervention Together With the Application of MSC-derived Exosomes on the Regenerative Capacity and Wound Healing of Cutaneous Ulcers in Diabetics	MSCs	To develop a therapeutic process to accelerate the healing of diabetic CCU, based on the correction of nutritional deficiencies, to improve the regenerative capacity, together with the application of exosomes from MSC in the wound, creating a microenvironment that favors tissue regeneration.	Maimónides Biomedical Research Institute of Córdoba (Spain)	2023-10-25
Burn wounds	NCT05078385	A Pilot Safety Study of Mesenchymal Stem Cell Derived Extracellular Vesicles for the Treatment of Burns Wounds	BMSCs	To treat patients with deep second degree burns of the skin with EV isolated from bone marrow derived MSCs.	Aegle Therapeutics (United States)	2024-11-20

Chronic Radiation Ulcer	NCT06793748	An Adaptive Two-Part, Phase 1/2, Multi-Center, Double-Blinded, Randomized, Controlled Study of the Safety and Efficacy of Topically Applied PEP-TISSEEL in Participants With Chronic Radiation Ulcer	Not applicable	Learn if drug Purified Exosome Product (PEP™) and Fibrin Sealant (TISSEEL® VH SD Kit) (PEP-TISSEEL) works to treat chronic radiation ulcer in adults and compare it with comparator TISSEEL.	Rion Inc. (United States)	2025-04-06

Skin Graft Donor Site Wounds	NCT04664738	A Phase I Open-Label Trail to Determine the Safety of PEP on a Skin Graft Donor Site Wound	Platelet	To determine the safety of a biological therapeutic PEP (composed of platelet derived extracellular vesicles enriched in anti-inflammatory and angiogenic growth factors) in participants who have skin graft donor site wounds.	Rion lnc. (United States)	2023-09-08

**Table 3:** A comprehensive overview of clinical trials investigating EV-based therapeutics for wound healing (as of April 2026) by conducting a key word search for “exosome” or “extracellular vesicle” on Chinese Clinical Trial Registry website.

Registration number	Scientific Title	Intervention group	Objective of Study	Primary Sponsor (Nation)	Date of Registration
ChiCTR2600122806	Single-Arm Interventional Clinical Study on the Safety and Efficacy of Exosomes in the Treatment of Refractory Wounds	Exosome gel	(1) Primary objective: To evaluate the safety of exosome therapy for refractory wounds. (2) Second research objective: To evaluate the efficacy of exosomes in treating refractory wounds.	The First Affiliated Hospital of Hainan Medical University (China)	2026-04-17
ChiCTR2600121779	A Single-Center, Prospective, Non-randomized, Open-lable Safety Study of Mesenchymal Stem Cell-Derived Exosomes in the Treatment of Diabetic Foot Ulcers	MSC-Exos: 5х10^10^ particles per dose	(1) To explore the safety and tolerable dose range of locally administered mesenchymal stem cell-derived exosomes in patients with diabetic foot ulcers; (2) To determine the recommended Phase II dose (RP2D) and collect preliminary data on efficacy, pharmacokinetic/pharmacodynamic profiles, and immunological characteristics.	The Second Affiliated Hospital of Zhejiang University School of Medicine (China)	2026-04-02
ChiCTR2600118237	Efficacy of umbilical cord mesenchymal stem cell-derived exosome encapsulated hydrogel in the treatment of diabetic foot wounds: a multicenter, randomized, open-label clinical study	Umbilical cord mesenchymal stem cell-derived exosome encapsulated hydrogel	To prove that unbilical cord mesenchymal stem cell-derived exosome hydrogel can promote the healing of diabetic foot wounds, and provide a new clinical solution to solve the problem of difficult healing of diabetic foot wounds.	Third Affiliated Hospital of Sun Yat-sen University (China)	2026-02-03
ChiCTR2600117662	Exploratory Clinical Study of iPSC-Derived Exosomes (GD-iExo-001) in Scarless Wound Healing	The extracellular vesicle particle concentration of GD001 is low-dose and high-dose group	To evaluate the safety, tolerability, and preliminary efficacy of GD001 for scar prevention.	Plastic Surgery Hospital, Chinese Academy of Medical Sciences and Peking Union Medical College (China)	2026-01-27
ChiCTR2500110430	Umbilical Cord Mesenchymal Stem Cell Exosomes Combined with Hydrogel for Treating Lower Limb Arterial Ischemic Ulcers: A Clinical Study on Safety and Efficacy	Umbilical cord mesenchymal stem cell exocrine combined with water gel	This study used a single center, randomized controlled, single blind trial design to evaluate the safety and preliminary effectiveness of umbilical cord mesenchymal stem cell exocrine combined with water gel in the treatment of lower limb arterial ischemic ulcer.	Lecong Hospital of Shunde Foshan(China)	2025-10-14
ChiCTR2500108441	Clinical Study on the Application of Stem Cells and Stem Cell-Derived Exosomes in Wound Healing	Take 1 mL of exosome solution at a concentration of about 1 mg/mL, mix it 1:2 in a homemade hydrogel, apply it evently to the wound area of the face	To evaluate the safety and efficacy of topical application of human umbilical cord mesenchymal stem cells (hUC-MSCs) and their derived exosomes in the treatment of acute wounds.	Air Force Medical Center (China)	2025-08-29
ChiCTR2500103555	Multi-center clinical trial study on the safety and effectiveness of stem cell-derived extracellular vesicles for wound repair and skin appendage regeneration	MSCs-EXOs are injected subcutaneously	Evaluate the safety and efficacy of locally injected exosomes derived from fetal umbilical cord mesenchymal stem cells in the treatment of chronic wounds and regeneration of skin appendages.	Chinese PLA General Hospital (China)	2025-05-30
ChiCTR2300075136	Evaluation of the safety and efficacy of stem cell-derived exosomes for promoting wound healing in clincial research protocols	Different dose groups: 10 μg/cm^2^, 20 μg/cm^2^, 33 μg/cm^2^, 50 μg/cm^2^, 67 μg/cm^2^, 90 μg/cm^2^ exosomes given after split-skin acquisition	(1) Major objective: To explore the safety of mesenchymal stem cell exosomes for wound healing; (2) Second oobjective: To evaluate the effectiveness of mesenchymal stem cell exosomes for wound healing.	First affiliated hospital of air force medical university (China)	2023-08-25

For the large-scale production of EVs, bioreactor-based 3D culture systems have achieved a significant breakthrough, overcoming the inherent limitations of conventional 2D culture using flat-bottomed flasks. When combined with the conventional differential UC, 3D culture produces 20-fold more exosomes than 2D cultures; furthermore, tangential flow filtration (TFF) in combination with 3D MSC culture further enhances the yield of exosomes by 7-fold [501]. Specifically, relative to traditional flask-based cell culture, integration of a herringbone microfluiic bioreactor with cell microcarriers has been demonstrated to enable batch-preparation of stem cell-derived exosomes, yielding substantially higher exosome quantities [502]. Moreover, hUCMSCs can be cultured in a fixed-bed bioreactor, in which polyethylene terephthalate fiber rolls constitute the fixed-bed layer, with a specifically developed serum-free medium employed [503]. Regarding advanced EV purification, free-flow isoelectric focusing (FFIEF) achieved 2- to 4-fold higher recovery and throughput of exosomes compared to conventional UC and size exclusive chromatography (SEC), suggesting that FFIEF can serve as an alternative tool for large-scale isolation of exosomes based on isoelectric point diversity [504]. In comparison to conventional UC, the membrane-based isolation processes led to a 32-41% increase in exosome yield and generated products with higher purity [505]. The integration of EVs into biomaterial matrices requires careful consideration of the timing and delivery method; post-isolation functionalization is generally preferred to preserve the integrity of both EV and other constituent components.

Unlike batch EV harvest from conditioned media, the biomaterial itself imposes scalability constraints (retain EV bioactivity during manufacturing process may not be compatible with cGMP-scale mixing, casting, or lyophilization). In preclinical research, EV loading into natural or synthetic biomaterial scaffolds is typically performed via manual, lab-scale incubation, adsorption, or simple physical embedding, with minimal standardized process parameter control and no unified critical quality attribute (CQA) monitoring system. However, cGMP manufacturing requires full-process standardized control over cell culture for EV source production, EV isolation and purification, biomaterial substrate fabrication, EV-integrated composite biomaterial, and post-formulation initial quality inspection, with mandatory traceability of every production link and strict elimination of heterogeneous impurities and process-related contaminants. The transition from laboratory-scale to clinical-scale of EV-loaded biomaterials presents non-trival challenges. Biomaterials carrying EVs require simultaneous control of (i) EV isolation/purity, (ii) biomaterial synthesis (including polymer crosslinking density, porosity), and (iii) EV loading efficiency. A close-loop, continuous manufacturing systems where EV-loading occurs during material assembly. EVs derived from hUCMSCs can be obtained under a cGMP-compliant protocol, with consistent characteristics and pre-clinical safety profile, thereby supporting future clinical translation as cell-free therapeutic agents [506]. The successful clinical translation of scaffold protein-engineered EVs requires a fundamental shift from laboratory-scale preparation toward robust, scalable, and GMP-compliant manufacturing processes. In this context, considerations in Chemistry, Manufacturing, and Controls (CMC) are key determinants of product safety, batch-to-batch consistency, and overall therapeutic efficacy [507].

### 6.2 Quality control

EV-loaded biomaterials suffer from a “double heterogeneity”: variability in EV populations and variability in biomaterial fabrication. For EVs, even clonal cell lines under ostensibly identical culture conditions yield batch-to-batch differences in size distribution, cargo (e.g., protein, miRNA, lipids), and surface ligand density factors that directly influence release kinetics from a matrix. The pore size, degradation rate, and mechanical properties of biomaterials vary with crosslinking density, which in turn alters EV retention and diffusion. the analysis platforms for evaluating the release of EVs from biomaterials primarily including nanoparticle tracking analysis (NTA), transmission electron microscopy (TEM), Western blot (WB), flow cytometer (FCM), bicinchoninic acid (BCA) kit analysis and fluorescence labeling (as summarized in Table S5). The current best practices recommend multiparametric quality control per MISEV2023 guidelines [508]. For the biomaterial component, design of experiments approaches can identify critical process parameters such as crosslinking time, temperature, and EV-to-polymer ratio that minimize functional variability. Table S6 provides a quality control framework (from production materials, key process parameter and process control, characterization of EVs, quality attributes, stability, and process validation) for biomaterials carrying EVs.

### 6.3 Long-term storage stability

The pure cell-free EV therapeutics typically adopt unified cryogenic storage at −80 °C to maintain the integrity of the exosome membrane structure and the biological activity of the internal cargo, with relatively clear storage and transportation specifications. However, repeated temperature fluctuations and long-term static preservation can easily cause EV membrane rupture, degradation of internal functional proteins and nucleic acid cargo, and premature release of EVs from the matrix, resulting in a sharp decline in therapeutic potency before clinical application. In contrast, EV-laden composite biomaterials face dual stability attenuation risks from both the biomaterial matrix and encapsulated EVs during long-term preservation and transportation. On the one hand, polymeric or hydrogel biomaterial matrices are prone to structural degradation, mechanical performance attenuation, and pore structure collapse during long-term cryopreservation, freeze-thaw cycles, or ambient temperature transportation, disrupting the sustained-release microenvironment of loaded EVs. On the other hand, the biomaterial microenvironment can alter the natural protective state of EVs. Storage of EVs in phosphate-buffered saline led to a pronounced decline in recovery, particularly for purified EV samples, across all temperatures tested and within several days of storage [509]. By contrast, PBS supplemented with human albumin and trehalose substantially improved both short-term and long-term EV preservation at −80 °C, conferred stability throughout repeated freeze-thaw cycles, and when employed as a diluent for downstream applications, markedly enhanced EV recovery [510].

### 6.4 Regulatory considerations

As a medical device combination product, biomaterials carrying EVs are subject to a stringent approval process to guarantee both safety and efficacy. The European Medicines Agency (EMA) regulates exosome-based therapeutics through a biflurcated framework. Under this framework, classification as an Advanced Therapy Medicinal Product (ATMP) depends on the nature of the cargo and primary mechanism of action [500]. Of note, the National Institutes for Food and Drug Control (NIFDC) and the National Medical Products Administration (NMPA) of China explicitly classified the “hyaluronic acid sodium exosome membrane liquid dressing” as a medical-device-combined product (Class III medical device) in 2025. This regulatory classification represents a significant breakthrough for EV-based products within China's regulatory system. The regulatory authorities strictly require the following evidence: (i) comparative characterization (free EVs vs. matrix-released EVs) including proteomic, lipidomic, and functional assays; (ii) validation of sterilization processes: given that most terminal sterilization methods (e.g., gamma irradiation, ethylene oxide) degrade EVs; aseptic processing combined with sterile filtration of EV solutions prior to matrix assembly is the current default, but this limits scalability; (iii) stability protocols for the final product: whereby EV-loaded biomaterials stored as lyophilized powders or hydrated gels must exhibit consistent biological activity throughout their shelf life, with predefined acceptable windows for degradation.

### 6.5 Potential immunogenicity

The immunogenicity risk of EV-loaded biomaterials is multifactorial and fundamentally distinct from that of free EVs. The risk can be categorized into three primary sources. (i) Donor-derived immunogenicity: EVs carry donor-cell-derived surface proteins (e.g., shock proteins, glycans) and nucleic acids (e.g., mitochondrial DNA, non-coding RNAs). These components may be recognized as damage-associated molecular patterns or pathogen-associated molecular patterns by patten recognition receptors on host immune cells, thereby triggering activation of macrophages, dendritic cells, and the complement cascades. (ii) Biomaterial-induced immunogenicity: Synthetic or natural polymers with degradation byproducts can elicit foreign body reactions, including protein adsorption, macrophage fusion into giant cells, fibrosis, and chronic inflammation. (iii) Manufacturing process-induced immunogenicity: During the encapsulation process of EVs, chemical crosslinking or mechanical shear forces may denature surface proteins, exposing hydrophobic patches or aggregation-prone regions that act as danger signals. Additionally, residual crosslinkers, endotoxins, or degradation products may further potentiate immune activation. Moreover, preclinical investigations particularly long-term implantation studies (more than six months) in large animal models remain conspicuously limited and should be accorded high priority in future research.

To mitigate these immunological and safety risks, EV-loaded biomaterials can be optimized by optimizing EVs source, engineering EVs surface and biomaterial design, improving cell viability and hemocompatibility, reducing oncogenic risk, and ongoing safety pharmacology. The mitigation strategies include (i) EVs source optimization: use EVs from autologous or hypoimmunogenic allogeneic sources; (ii) EV surface engineering: shield EV surface epitopes by PEGylation or incorporating complement regulatory proteins; (iii) biomaterial design: employ biocompatible, non-immunogenic materials, minimize endotoxin levels via stringent purification, or incorporate immunomodulatory cues into the biomaterials to promote regulatory T cell responses; (iv) cell viability: cell viability of EV-loaded biomaterial platforms complies with international standards of safety (over 80%, ISO 10993-5), indicating that these platforms are relatively non-toxic; (v) hemocompatibility: hemolysis rate of EV-loaded biomaterial platforms is less than 5%, in line with internal standards (ISO 10993-4) [511], demonstrating that these platforms have good hemocompatibility; (vi) safety pharmacology: assess biodistribution using fluorescent EVs in small and large animal models, and monitor for off-target organ toxicity (such as heart, liver, spleen, lung, kidney) [209]; (vii) oncogenic risk assessment: screen EV cargo for known oncogenes and use producer cells with stable genomic profiles. Combining these approaches will enable safe and effective EV-loaded biomaterial platforms for next-generation therapeutics in wound healing.

## 7 Summary and future perspectives

### 7.1 Summary

In summary, biomaterials incorporating EVs have demonstrated considerable promise for wound healing applications. This review systematically examines the fundamental fabrication strategies that guide the design of various application forms of EV-loaded biomaterial platforms, including nanocomposites, hydrogels, microneedle patches, electrospun fiber membranes, 3D-printed scaffolds, and microspheres. Moreover, the release mechanisms, release profiles, and kinetic models describing EV release from biomaterial platforms are summarized. Stimuli-responsive release strategies leveraging wound microenvironmental cues (such as pH, elevated ROS and MMP concentrations, as well as externally applied triggers enable spatiotemporally controlled therapeutic delivery, thereby maximizing efficacy while minimizing off-target effects. Clinical challenges associated with biomaterials carrying EVs are also discussed, including compliance with cGMP for scalable production, reproducibility, long-term storage stability, regulatory considerations, and potential immunogenicity. This review aims to provide a comprehensive overview of the current landscape of EV-loaded biomaterials and to further promotes their development and clinical translation in wound healing. Continuous progress is anticipated in achieving precise EV delivery to target cells or tissues and in developing next-generation biomaterial-based EV delivery platforms.

### 7.2 Emerging trends and future directions

With the development of regenerative medicine and the increasing market demand for skin wound repair, EV-based composite biomaterials have garnered increasing attention. This review comprehensively summarizes the common fabrication strategies of various composite biomaterials, which prolong the release of EVs. The recent progress in various wounds of EV-loaded biomaterials is also reviewed. From our limited knowledge, EV-loaded biomaterials have made impressive progress, but significant challenges remain, and the journey toward clinical application is ongoing. The current limitations and future perspectives include as follows:

(1) **Production and quality control standardization**: The isolation, purification and preservation protocols of EVs are not yet standardized, which makes it difficult to guarantee the yield, purity, and biological integrity. Despite various strategies have been developed for the isolation, purification and preservation of EVs, the standardization gap creates substantial barriers to industrial-scale production and clinical translation of EV-based therapeutics.
(2) **Structural and functional stability:** EVs face critical challenges in therapeutic applications due to their intrinsic instability in physiological conditions. Their structural integrity is easily compromised by enzymatic degradation and immune recognition clearance. In addition, under the dynamic physiological microenvironment characterized by oxidative stress or inflammatory responses, the bioactivity retention of encapsulated therapeutic cargoes, including non-coding RNAs (like miRNA, circ-RNA, lncRNA) and functional proteins, is substantially diminished due to membrane destabilization and cargo leakage.
(3) **Targeted delivery precision of EVs**: Although the composite biomaterials have achieved the sustained release of EVs, their cell-specific targeting remains a critical unmet challenge. Currently, the distribution of native EVs in vivo is nonspecific, where EVs undergo indiscriminate cellular uptake across multiple cell types. Emerging strategies focusing on surface engineering of EVs through ligand conjugation or membrane modification are showing promising progress in enhancing tissue-specific targeting capabilities.
(4) **Spatiotemporally adaptive regulation of wound healing dynamics**: The wound healing process involves four overlapping phases: hemostasis, inflammation, proliferation, and remodeling. Although the recent advances in stimuli-response biomaterial platforms have enabled sustained EVs delivery for wound repair, current systems mainly focus on the unidirectional release kinetics and limited microenvironmental responsiveness. Future development should prioritize multistage-adaptive EV delivery systems capable of (i) phase-specific biomarker recognition, (ii) spatial-temporal controlled cargo release, and (iii) bidirectional feedback regulation, to achieve perfect wound repair.
(5) **Artificial intelligence in EV loading design and release prediction**: Artificial intelligence (AI) offers a powerful computational framework to expedite the design of cargo loading into EVs and the prediction of their subsequent release profiles. By training machine learning models on relevant physicochemical attributes (such as the compositional characteristics of EV membranes and release kinetics under defined physiological or pathological microenvironments. This predictive capacity facilitates the rational selection of optimal loading parameters, thereby enhancing the precision and efficiency of EV-based therapeutic development.
(6) **Scalable production EV-loaded microspheres by microfluidic technology**: Microfluidic technology offers distinct advantages in precise fluid manipulation and microscale reaction regulation, enabling the fabrication of EV-loaded microspheres with high precision and uniformity. Key physicochemical properties such as particle size, porosity, and internal architecture, can be readily tuned through microfluidic design. Furthermore, microfluidic systems facilitate continuous and automated assembly processes, which can be scaled up via parallel-array chip configurations. This scalability supports high-throughput, clinically relevant production of EV-embedded microspheres with consistent quality and standardized attributes.
(7) **Treatment and monitoring integration**: While current stimulus-responsive EVs delivery systems show potential to respond to the change of wound microenvironment, next-generation platforms should evolve into self-regulating therapeutic systems that not only real-time monitor wound healing process, but also dynamically adjust EVs release kinetics. For instance, combined with wireless transmission technology, the monitoring data is linked to the cloud AI analysis platform to form an integrated platform of “monitoring-analysis-treatment” to optimize the therapeutic effects.
(8) **Clinical translation barriers**: Despite growing market demand for EV-based therapeutics, current EV-loaded biomaterial systems are in the laboratory-scale production, predominantly in preclinical development. The clinical validation through phased trials (Phase I-III) is urgently needed for further scalable production of EVs-loaded biomaterials.

## Ethics approval and consent to participate

Our submission is a review article, which does not include a clinical study and not involve experimentation on animals and human subjects.

## CRediT authorship contribution statement

**Shengqiu Chen:** Conceptualization, Formal analysis, Funding acquisition, Project administration, Writing – original draft. **Zhiliang Zhao:** Formal analysis, Investigation, Writing – original draft. **Lige Tian:** Formal analysis, Investigation, Validation. **Shengnan Cui:** Formal analysis, Visualization. **Xi Liu:** Investigation, Methodology. **Hao Meng:** Investigation, Methodology. **Yi Xie:** Formal analysis, Funding acquisition, Writing – review & editing. **Dawei Chen:** Formal analysis, Investigation. **Xingwu Ran:** Conceptualization, Funding acquisition, Supervision, Writing – review & editing. **Changsheng Zhao:** Conceptualization, Supervision, Writing – review & editing. **Xiaobing Fu:** Conceptualization, Funding acquisition, Supervision, Writing – review & editing. **Cuiping Zhang:** Conceptualization, Funding acquisition, Supervision, Writing – review & editing.

## Declaration of competing interest

The authors declare that they have no known competing financial interests or personal relationships that could have appeared to influence the work reported in this paper.
