# Supplementary material for: Biomaterial-based extracellular vesicle delivery systems for wound healing: From fabrication to applications
Source: Bioact Mater. 2026 Aug 5;66:1202–51. doi: 10.1016/j.bioactmat.2026.06.042 (PMC13472037; doi:10.1016/j.bioactmat.2026.06.042)
Supplement: Supplementary file 1 [file mmc1.pdf]

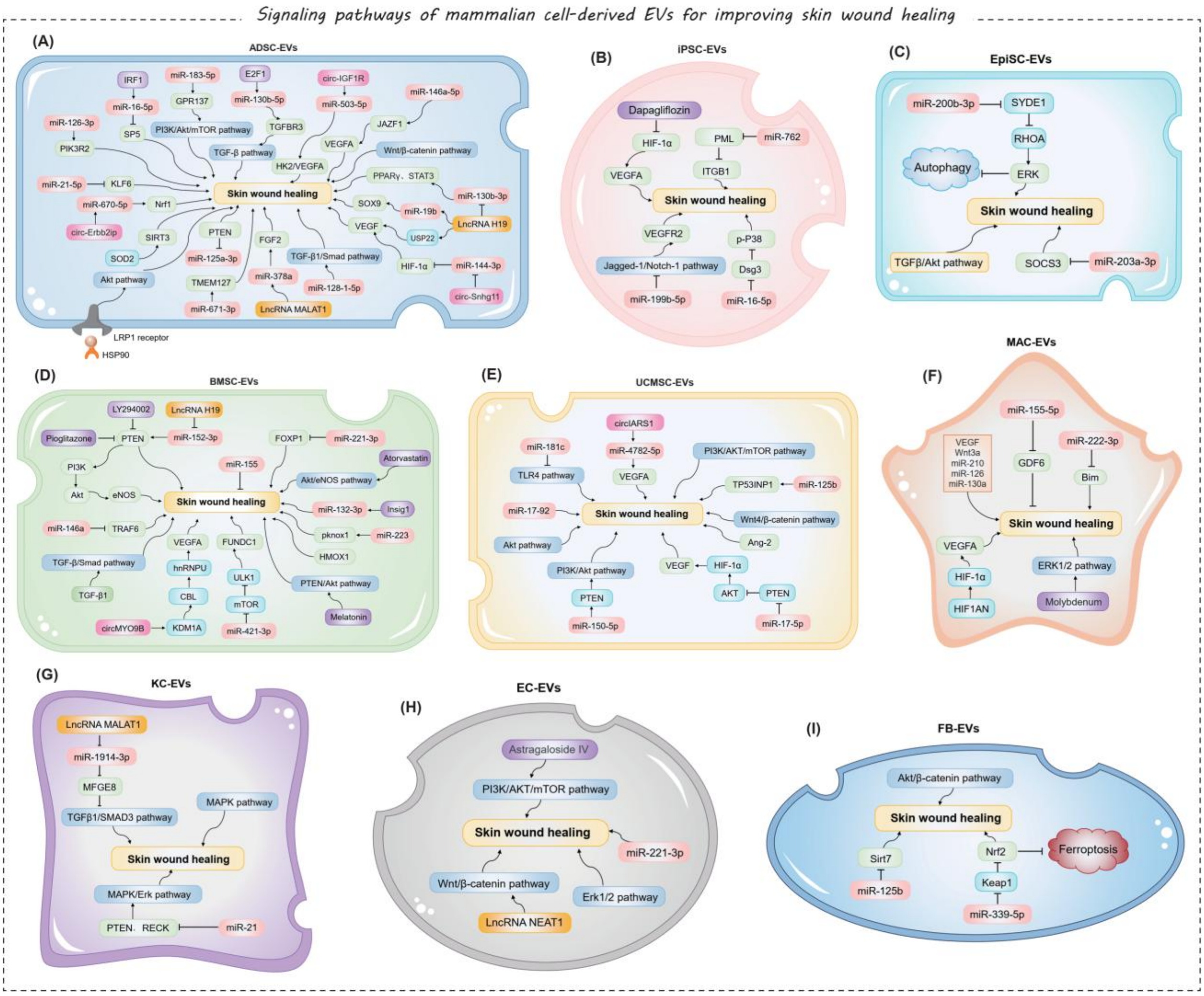

Figure S1. Schematic illustration of signaling pathways of mammalian cell-derived EVs for promoting skin wound healing. (A) ADSC-EVs: adipose-derived stem cell-derived EVs; (B) iPSC-EVs: induced pluripotent stem cell-derived EVs; (C) EpiSC-EVs: epidermal stem cell-derived EVs; (D) BMSC-EVs: bone marrow stem cell-derived EVs; (E) UCMSC-EVs: umbilical cord mesenchymal stem cell-derived EVs; (F) MAC-EVs: macrophage-derived EVs; (G) KC-EVs: keratinocyte-derived EVs; (H) EC-EVs: endothelial cell-derived EVs; (I) FB-EVs: fibroblast-derived EVs.

Table S1. Comparison of EVs derived from different mammalian cell sources for wound treatment.

Cell category	EV subtypes	Isolation method	Signaling pathway	Effects (*in vitro*)	Effects (*in vivo*)	Ref.
ADSCs	miR-126-3p-loaded ADSC-Exos	Ultracentrifugation	Targeting PIK3R2	• Promote proliferation and migration of fibroblasts and angiogenesis of HUVECs	• Accelerate wound healing of full-thickness skin defects	Ma *et al*. (2021) [1]
	miR-138-enriched Exos	Centrifugation, ultrafiltration	SIRT1/PTEN pathway	• Promote cell migration, apoptosis inhibition of HSFs in HG culture	• Accelerate wound healing in diabetic rats by promoting angiogenesis	Li *et al*. (2023) [2]
	miR-671-3p-overexpressed hADSC-EXO	Centrifugation	miR-671-3p/TMEM127 axis	• Promote HUVECs prolfieration, migration and invasion<br>• Promote fat graft angiogenesis and adipogenic differentiation	• Not applicable	Hao *et al*. (2024) [3]
	MiR-21-5p-carried ABs	Centrifugation	miR-21-5p targets KLF6	• Induce M2 macrophages polarization<br>• Enhance angiogenesis and fibroblast migration<br>• Elevate expression of miR-21-5p in macrophages	• Accelerate wound healing process in mice<br>• Promote the formation of granulation tissues and blood vessels<br>• Increase collagen deposition and neovascularization<br>• Reduce inflammatory infiltration in the tissues	Li *et al*. (2022) [4]
	miR-92a-1-5p-overexpression-	Ultracentrifugation	Conserve miR-92a-1-	• Reduce high glucose-induced NETs formation	• miR-92a-1-5p knowdowm obstructed the promotion	Li et al. (2026) [5]

ADMSCs-ex		5p/S100A9 pathway	by targeting S100A9	effect of ADMSCs-ex on diabetic wound healing and NETs formation	
miR-125a-3p-overexpressed HADSCs-exosome	Ultracentrifugation	miR-125a-3p/PTEN	• Promote the viability, migration, and angiogenesis of HUVECs	• Promote angiogensis and wound healing in mice	Pi *et al*. (2021) [6]
ADSC-EXOs	Differential centrifugation	eHSP90/LRP1/AKT axis	• Promote the proliferation and migration of HaCaTs, fibroblasts and HUVECs<br>• Promote angiogenesis in HUVECs<br>• Protect skin cells against damage from hypoxia and oxidative stress<br>• eHSP90 contributed to the effects on cell migration and protection	• Promote diabetic wound closure via eHSP90<br>• Promote ROS scavenging and angiogenesis via surface HSP90	Ren *et al*. (2022) [7]
ADSC-Exos	Supercentrifugation	miR-128-1-5p/TGF-β1/Smad pathway	• Enhance RSFs proliferation and migration under HG conditions while reducing the expression of pro-fibrotic markers	• Promote diabetic wound healing<br>• Inhibit skin fibrosis in a rat model of type 2 diabetes by decreasing the expression levels of α-SMA and Col 1	Liang *et al*. (2024) [8]
ADSCs$^{TSPAN6+}$-Exos	Ultracentrifugation	Syntenin-1	• Support the prolfieration, and migration and tube formation of HUVECs<br>• Enhance the prolfieration	• Promote wound healing in rat<br>• Enhance collagen deposition and neovascularization at	Qiao *et al*. (2024) [9]

			and migration of HSFs	the location of lesion	
circ-IGF1R-HEV	Centrifugation	circ-IGF1R/miR-503-5p/HK2/VEGFA axis	• Not applicable	• Promote wound healing in diabetic mice<br>• Enhance angiogenesis	Shi *et al.* (2024) [10]
ADSC-exos	Exosome isolation reagent (Invitrogen)	H19/miR-130-3p/PPARγ/STAT 3	• Promote macrophages into M2 polarization, thus contributing to cell priliferation, migration and angiogenesis	• Accelerate cutaneous wound healing	Li *et al.* (2019) [11]
ADSC-Exo	Differential ultracentrifugation	Not applicable	• Promote HUVEC prolfieration<br>• Regulate expression of has-miR-760 and has-miR-423-3p<br>• Regulate related genes including ITGA5 and HDAC5	• Promote repair and vascularization of prefabricated flap graft wounds *in vivo*	Xiong *et al.* (2020) [12]
hADSC exosomes	ExoQuick solution (Systems Biosciences, CA, USA)	miR-378a/FGF2 axis	• Increase viability and migration ability of HSFs<br>• Reduce apoptosis via mediating MALAT1	• Promote cutaneous wound healing in mice	Pi *et al.* (2022) [13]
Exosomes from lincoo511-overexpressing ADSCs	ExoQuick Exosome Precipitation Solution (EXOQ5A, System Biosciences, USA)	Suppressing PAQR3-induced Twist1 ubiquitin degradation	• Restore the impaired EPCs proliferation, migration and angiogenesis under HG	• Promote rat diabetic foot ulcer healing by accelerating angiogenesis	Qiu *et al.* (2021) [14]

	ADSC$^{E2F1-/-}$-Exos	Ultracentrifugation	miR-130b-5p/TGFBR3 axis	• Promote proliferation, migration and collagen formation of fibroblasts	• Accelerate wound healing by promoting collagen formation and angiogenesis	Yu *et al.* (2023) [15]
	ADSC-Exos	Ribo™ Exosome Isolation Reagent (RiboBio, Guangzhou, China)	lncRNA H19/miR-19b/SOX9 axis	• Accelerate the proliferation, migration and invasion of HSF cells in H19	• Promote skin wound healing via H19 in mice	Qian *et al.* (2021) [16]
	ADSC-Exos	Centrifugation	miR-146a-5p/JAZF1 axis	• Mitigate prolfieration, migration and angiogenesis in HUVECs<br>• Enhance angiogensis through up-regulating miR-146a-5p	• Promote wound healing in diabetic mice with increased collagen I synthesis and neovascularization	Che *et al.* (2024) [17]
	FEXO	Ultracentrifugation	miR-183-5p/GPR137/PI3K/Akt/mTOR axis	• Enhance autophagy and viability and alleviate pyroptosis in endothelial cells	• Promote angiogenesis and autophagy, inhibit oxidative stress and alleviate apoptosis and pyroptosis in random-pattern skin flaps	Zhang *et al.* (2024) [18]
	IRF1-Exo	Differential ultracentrifugation	IRF1/miR-16-5p/SP5 axis	• Trigger fibroblasts prolfieration and migration<br>• Trigger endothelial cell angiogensis	• Facilitate wound closure in diabetes mellitus rats by enhancing collagen deposition, blood vessels formation	Wu *et al.* (2023) [19]
iPSCs	iPSCs-KCs-Exos	Serial ultracentrifugation	miR-762 tagets PML through ITGB1	• Promote the migration of keratinocytes and HUEVCs	• Accelerate the healing of deep second-degree burns in mice	Bo *et al.* (2022) [20]

	hiPSC-ECs-Exo	Ultracentrifugation	miR-199b-5p	• Promote cell migration and prolfieration, as well as angiogenesis via inhibiting Jagged 1/Notch 1 signaling	• Not applicable	Ye *et al*. (2019) [21]
BMSCs	Exo-HMOX1	Ultracentrifugation	HMOX1	• Promote the proliferation and migration of fibroblasts and keratinocytes<br>• Facilitate the migratory and angiogenic activities of HUVECs	• Accelerate cutaneous wound healing in diabetic mouse by promoting re-epithelialization and vascularization	Cheng *et al*. (2023) [22]
	EXO-miR-146a-5p	Hieff® Quick exosome isolation kit Plus (YESEN, Shanghai, China)	TRAF6	• Protect HUVECs from HG-induced injury by inducing M2 polarization	• Accelerate diabetic wound healing in mice via enhancing angiogenesis and M2 polarization	Zhou *et al*. (2023) [23]
	Exosomal IncRNA H19	Ultracentrifugation	IncRNA H19/miR-152-3p/PTEN axis	• Regulate fibroblast proliferation, migration, and apoptosis	• Accelerate wound healing process of mice with DFU	Li *et al*. (2023) [24]
	MSCs-Exos carrying circMYO9B	Total Exosome Isolation reagent (ThermoFisher Scientific)	hnRNPU/CBL/KDM1A/VEGFA axis	• Regulate the proliferation, migration and angiogensis of HG-induced HUVECs via upregulating KDM1A or suppressing CBL	• Accelerate wound healing in mice with DFU by translocating hnRNPU and enhancing VEGFA expression	Wang *et al*. (2024) [25]
	miR-155-inhibitor-loaded MSC-derived exosomes	Differential centrifugation	miR-155-inhibitor	• Accelerate wound healing *in vitro* via modulating the expression of miR-155, FGF-7, VEGF, and MMP	• Promote diabetic wound healing by enhancing collagen deposition, angiogenesis, and re-epithelialization	Gondaliya *et al*. (2022) [26]

Hypo-Exo	ExoQuick-TC (SBI, USA)	mTOR/ULK1/FUNDC1 pathways	• Protect vascular endothelial cells from I/R injuried *in vitro*<br>• Inhibit autophagy reversed the biological effects *in vitro*	• Improve survival rate of I/R injuried skin flaps through attenuating I/R injury<br>• Reduce inflammation, ROS, apoptosis in I/R injuried flaps model<br>• Protect vascular endothelial cells from I/R injuried *in vivo*<br>• Acitivate autophagy by suppressing mTOR via miR-421-3p	Deng *et al*. (2023) [27]
MET-Exos	Ultracentrifugation	LINC-PINT/miR-139-3p/FOXC2 axis	• Promote migration, tube formation and angiogensis in HUVECs *in vitro*	• Promote wound closure, re-epithelialization, collagen deposition and neovascularization in a diabetic mouse model using STZ injection	Gu *et al*. (2025) [28]
Insig1-exo	Centrifugation	miR-132-3p	• Promote the proliferation, migration, and angiogenic abilities of mouse dermal fibroblasts by transporting miR-132-3p	• Accelerate wound healing in diabetic mice by promoting epithelialization, collagen deposition and reducing inflammatory response	Zheng *et al*. (2024) [29]
Exosomal miR-221-3p	Ultracentrifugation	Mediating FOXP1/SPRY1 axis	• Promote angiogenesis in HG-treated HUVECs through FOXP1/SPRY1 axis	• Promote wound healing in diabetic mice	Qiu *et al*. (2024) [30]
MSC-secreted	ExoQuick-TC	miR-223 by	• Promote M2 polarization	• Enhance cutaneous wound	He *et al*.

	exosomes	(ExoQuick, System Biosciences)	targeting pknox1		healing and skew macrophages to the M2 phenotype	(2019) [31]
USCs	H-exo	Ultracentrifugation	Tageting SERPINE1 through miR-486-5p	• Enhance proliferation, migration, and tube formation of HUVECs	• Contribute to the diabetic wound healing by inhibiting inflammation, re-epithelialization, collagen deposition, and vascularization	Fan *et al.* (2024) [32]
	USC-Exos	Exoquick Exosome Precipitation Solution (System Biosciences, USA)	Transferring DMBT1 protein	• Enhance the proliferation and migration of HaCaTs and HSFs<br>• Augment the angiogenic activities of HMECs	• Exosomal DMBT1 accelerates cutaneous wound healing in diabetic mice<br>• Exosomal DMBT1 promotes angiogesis in the wound sites of diabetic mice	Chen *et al.* (2018) [33]
UCB-MSCs	UCB-MSC-exo	Gradient centrifugation	miR-21-5p and miR-125b-5p<br>TGF-β	• Promote the prolfieration and migration of EPSCs and HDFs	• Accelerate wound closure in full-thickness skin-wounded rats<br>• Improve regeneration and regulate collagen distribution in wound healing of rats<br>• Suppresses scar formation by inhibiting myofibroblast differentiation	Zhang *et al.* (2021) [34]
UCMSCs	EGF-Exos	Grandient	Not applicable	• Promote the proliferation,	• Contribute to the repair of	Gong *et al.*

	ultracentrifugation		migration, and angiogenesis of HUVECs, and have anti-inflammatory effect	diabetic wound	(2024) [35]
Nr-CWS-Exos	Ultracentrifugation	Upregulating circIARS1/miR-4782-5p/VEGFA axis	• Advance the angiogenesis of HUVECs	• Accelerate wound healing of diabetic mice by facilitating the angiogenesis of wound tissues	Li *et al*. (2023) [36]
Ang-2-enriched HucMSC-Exs	Centrifugation	Ang-2	• Promote HUVECs prolfieration, migration and tube formation	• Promote cutaneous wound healing and angiogenesis in a rat model of deep-second-degree burn injury	Liu *et al*. (2020) [37]
HucMSC-EVs	Ultracentrifugation	miR-17-5p/PTEN/AKT/HIF-1α/VEGF pathway	• Promote prolfieration, migration, and tube formation in HG-treated HUVECs	• Enhance cutaneous wound healing and angiogenesis in diabetic mice	Wei *et al*. (2022) [38]
HucMSC-sEVs	Ultracentrifugation	Restore Ca^{2+} homeostasis with upregulated TRPC6	• Reduce apoptosis and inflammatory damage caused by AGE-BSA in dermal fibroblasts<br>• Enhance the ability of dermal fibroblasts to secrete collagen-like ECM	• Promote wound healing in diabetic rats<br>• Promote decent re-epithelializtion, complete epidermal architecture establishment and angiogenesis with an intact lumen structure	Wang *et al*. (2024) [39]
UCMSC-exo/eNOS	Ultracentrifugation	Multi-pathway activation, PI3K/Akt/mTOR and	• Promote proliferation and migration of fibroblasts and HUVECs<br>• Counteract oxidative	• Promote wound closure and stromal remodeling in diabetic mice<br>• Promote angiogenesis in	Zhao *et al*. (2023) [40]

		FAK/ERK1/2, STAT3 and Smad3	damage and restore biological functions in fibroblats and HUVECs treated with HG<br>• Reverse oxidative stress-mediated damege to HUVECs	diabetic mouse<br>• Reduce inflammatory cell infiltration and the inflammatory response at the wound site<br>• Reshape the immune microenvironment of wound tissue	
hUCMSC-ex	PureExo® Column (101Bio company, CA, USA)	miR-181c expression in exosomes by downregulating the TLR4 signaling pathway	• Suppress LPS-induced macrophage inflammation	• Alleviate the inflammatory response of rats after burn injury	Li *et al*. (2016) [41]
HUC-MSCs-Exos	Filtration and iltracentrifugation	miR-150-5p regulates PI3K/AKT pathway by mediating PTEN	• Promote the proliferation and migration of H_2O_2-induced HaCaTs and inhibit the apoptosis<br>• Exosomal miR-150-5p effectively improves H_2O_2-induced injury of HaCaTs	• Not applicable	Xiu *et al*. (2022) [42]
miR-17-92-Exos	Ultracentrifugation	miR-17-92	• Protect HUVECs against erastin-induced ferroptosis	• Enhance faster wound healing by inducing angiogenesis and inhibiting ferroptosis	Nie *et al*. (2023) [43]
H-Exo	Gradient	Exosomal miR-	• Induce proliferation and	• Enhance skin wound repair	Zhang *et*

		centrifugation	125b/TP53INP1 signaling	migration of HUVECs	by promoting cell growth and migration and decrease cell apoptosis	*al*. (2021) [44]
FDMSC	FDMSC exosomes	ExoQuick-TC kit (SBI, USA)	Activating Notch signaling	• Enhance prolfieration, migration, and secretion of adult dermal fibroblasts	• Accelerate cutaneous wound healing by promoting cell proliferation, ECM deposition, and reepithelialization in the wound area	Wang *et al*. (2019) [45]
ESC	ESC-Exos	Differential centrifugation/ultracentrifugation	miR-200a/Keap1/Nrf2	• Ameliorate endothelial senescence and aging-related angiogenic dysfunction induced by D-gal<br>• Rejuvenate senescent endothelial cells through activating Nrf2	• Promote pressure ulcer wound healing in aged mice<br>• Enhance angiogenesis and ameliorate vascular endothelial cell senescence in the wound sites of mice	Chen *et al*. (2019) [46]
plaMSC	plaMSC-Ex	Ultracentrifugation	Not applicable	• Promote HaCaTs prolfieration and migration	• Promote diabetic wound healing in STZ-induced rats	Peng *et al*. (2024) [47]
hAMSC	hAMSC-Exos	Ultracentrifugation	Not applicable	• Facilitate the prolfieration, migration and angiogenic activities of HUVECs treated with HG	• Accelerate the healing of diabetic wounds by improving angiogenesis	Fu *et al*. (2023) [48]
	hAMSC-Exo	Ultracentrifugation	miR-135a/LATS2	• Promote migration in BJ cells<br>• Regulate the expression of migration-associated	• Promote wound healing and epithelialization in rats	Gao *et al*. (2020) [49]

				proteins		
SHED	SHED-Exo	Ultracentrifugation	AKT, ERK1/2, and STAT3 signaling pathway	• Regulate macrophagy function by manipulating autophagy	• Ameliorate the delayed wound healing process in LPS-induced mouse<br>• Relieve itch responses during wound healing	Xie *et al*. (2022) [50]
DPSCs	DPSC-Exos	Ultracentrifugation	Cdc42/p38 MAPK pathway	• Enhance the angiogenic activities of HUVECs	• Promote cutaneous wound healing in mice by promoting angiogenesis	Zhou *et al*. (2022) [51]
ESCs	EpiSC-EXOs	Ultracentrifugation	miR-200b-3p mediated downregulation of SYDE1/MAPK/autophagy pathway	• Alleviate apoptosis of HUVECs in a high-glucose environment	• Accelerate diabetic wound healing by promoting angiogensis	Xu *et al*. (2023) [52]
	ESCs-Exo	Centrifugation, ultrafiltration, ultracentrifugation	Activation of TGFβ and protein kinase B signaling pathways	• Promote proliferation and migration of DFBs<br>• Regulate macrophage polarization	• Accelerate diabetic wound healing in db/db mice by dereasing wound inflammation and promote wound cell prolfieration and angiogenesis	Wang *et al*. (2022) [53]
	EpiSC-EXOs	Ultracentrifugation	miR-203a-3p/SOCS3	• Induce M2 macrophage polarization<br>• Reduce SOCS3 expression	• Promote diabetic wound healing and macrophage M2 polarization in db/db mice	Yang *et al*. (2023) [54]
Macrophages	M2-Exos	Total exosome isolation reagent	GDF6-Akt axis	• Manipulate angiogenesis in HUVECs	• M2-like macrophage-derived exosomes and miR-	Lou *et al*. (2023)

	(Thermo Fisher Scientific, Carlsbad, CA)			155-5p inhibitors as promising therapeutics against diabetic foot ulcers	[55]
M2-exo	ultracentrifugation and ultrafiltration	HIF1AN/HIF-1α/VEGFA	• Enhance the proliferation, migration and tube formation of HUVECs	• Promote skin flap survival by facilitating choke vessels remodeling and neovascularization	Luo *et al*. (2023) [56]
Mo-Exo	Ultrafiltration and Total exosome isolation reagent (Invitrogen, USA)	Activating ERK1/2 signaling pathway	• Promote the angiogenic capacity of HUVECs<br>• Ameliorate inflammation and enhance the M2 polarization of macrophages<br>• Enhance angiogenesis partially via the activation of ERK1/2 signaling pathway	• Accelerate wound healing process in rats via angiogenesis promotion and immune regulation by encapsulating Mo-Exo into GelMA hydrogel	Deng *et al*. (2024) [57]
MAC-EVs	Ultracentrifugation	Not applicable	• Increase cellular proliferation and migration in recipient ECs<br>• Promote tube formation	• Increase the formation of new and large vessels in Matrigel plug mouse model	Gangadaran *et al*. (2020) [58]
Exos	Ultracentrifugation	Not applicable	• Improve angiogenesis in HG-HUVECs<br>• Promote cell migration and proliferation in HG-HUVECs via anti-inflammation	• Promote wound healing in type 1 diabetic rats by inhibiting the secretion of pro-inflammatory enzymes and cytokines and enhancing angiogenesis and re-epithelialization at the	Li *et al*. (2019) [59]

					• wound sites	
	ATM-Exos	Exosome Qucik Extraction solution	miR-222-3p, Bcl2l11/Bim	• Not applicable	• Accelerate wound healing by in db/db mice	Xia *et al*. (2023) [60]
Fibroblasts	DF-Ex	Differential centrifugation	Activate Akt/β-catenin pathway	• Protect HUVECs from HG damage	• Accelerate cutaneous wound healing in diabetic rats	Han *et al*. (2021) [61]
	exosomes^{Young}	Ultracentrifugation	miR-125b/Sirt7 signaling	• Activate fibroblast migration and increase fibrosis<br>• Promote migration and FMT by delivering miR-125b	• Accelerate wound healing by guiding phenotypic switch to myofibroblasts	Xia *et al*. (2022) [62]
	ABs	Differential centrifugation	miR-339-5p/KEAP1/Nrf2 axis	• Inhibit ferroptosis and promote endothelial cell survival via miR339-5p/KEAP1 *in vitro*	• Promote ischaemic flap survival *in vivo*<br>• Inhibit oxidative stress and cell death *in vivo*<br>• Inhibit ferroptosis and promote flap survival via miR-339-5p and KEAP1-Nrf2 axis *in vivo*	Yu *et al*. (2024) [63]
Keratinocytes	MV miR-21	Centrifugation	• Down-regulate PTEN and RECK in protein level<br>• Activate MAPK/ERK	• Promote fibroblast migration and fibroblast-mediated angiogenesis<br>• Modulate pro-inflammatory interleukins and MMPs in fibroblasts	• Promote diabetic cutaneous wound healing in the rat model<br>• Facilitate fibroblast differentiation	Li *et al*. (2019) [64]

			signaling cascade			
	HaCaT-derived EVs	TIM-4 affinity purification method	Activate MAPK pathway	• Enhance the migration of HaCaTs and fibroblasts	• Not applicable	Glady *et al.* (2021) [65]
	KCs-Exo carring MALAT1	Ultracentrifugation	MALAT1/miR-1914-3p/MFGE8 signaling axis	• Regulate macrophage phagocytosis, apoptosis, and polarization via TGFβ1/SMAD3 siganling pathway	• Promote wound healing in mice with diabetes mellitus by promoting collagen deposition, ECM remodeling, and expression of MFGE8, VEGF, and CD31 but reduce the expression of TGFβ1 and SMAD3	Kuang *et al.* (2023) [66]
Endothelial cells	Exos	Ultrafiltration and centrifugation	Not aplicable	• Promote angiogenesis-related genes expression, including FGF-1, eNOS, IL-8, ANG-1, E-selectin, VEGFA, VEGFR-2, CXCL-16<br>• Downregulate MMP-9 expression, in HMECs	• Promote wound healing in diabetic rats by promoting endothelial function	Li *et al.* (2016) [67]
	ASIV-EPCexos	Centrifugation	Activate PI3K/AKT/mTOR pathway	• ASIV promotes EPCexo secretion	• Promote type I diabetic wound healing<br>• Facilitate BrdU and VEGF expression and modulating CD31 and α-SMA expression<br>• Promote angiogenesis	Xiong *et al.* (2020) [68]

				• Inhibit inflammation	
H_2O_2-HUVEC-Exos	Ultracentrifugation	Lnc NEAT1/Wnt1/β-catenin axis	• Enhance the proangiogenic abilities of EPCs<br>• Significantly upregulate Lnc NEAT1 in EPCs	• Enhance the survival of skin flaps<br>• Increase neovascularization of skin flaps and recruitment of EPCs	Guo *et al*. (2022) [69]
EPC-Exos	Ultrafiltration and ultracentrifugation	Activate Erk1/2 signaling	• Pro-angiogenic effects on HMECs	• Accelerate cutaneous wound healing in STZ-induced diabetic rats<br>• Promote angiogenesis in the wound sites of diabetic rats	Zhang *et al*. (2016) [70]
EPC-Exo	Centrifugation	miR-221-3p	• Not applicable	• Promote wound healing in diabetic mice by promoting angiogenesis	Xu *et al*. (2020) [71]

Abbreviations: epidermal stem cells (EpiSCs, ESCs); urine-derived stem cells (USCs); angiopoietin-2 (Ang-2); hypoxic adipose stem cells exosomes (HypADSCs-exo); human umbilical cord MSCs (hUC-MSCs); exosomes derived from ADSCs preconditioned with H_2O_2 (H_2O_2-ADSC-exos); human adipose-derived mesenchymal stem cells (HADSCs); exosomes derived from TSPAN6 overexpression-treated ADSCs (ADSCs$^{TSPAN6+}$-Exos); IRF1 overexpression-modified exosomes (IRF1-Exo); botulinum toxin type A (BTX-A); EGFL6-preconditioned exosomes (EGF-Exos); HOX transcript antisense RNA (HOTAIR); EVs from MSCs transfected to overexpress long non-coding HOTAIR (HOTAIR-MSC EVs); *Nocardia rubra* cell skeleton (Nr-CWS); diabetic fibroblasts (DFB); bone marrow-derived macrophages (BMDMs); diabetic foot ulcer (DFU); kruppel like factor 6 (KLF6); M2 macrophages (M2-exo); molybdenum-inspired macrophage-derived exosome (Mo-Exo); hypoxic human umbilical vein endothelial cells (H-Exos); human umbilical cord mesenchymal stem cell-derived exosomes (HUC-Exos); high glucose (HG); endothelial progenitor cells (EPCs); EPC-derived exosomes (EPCexos); astragaloside IV-stimulated EPCexos (ASIV-EPCexos); human skin fibroblasts (HSFs); human microvascular endothelial cells (HMECs); diabetic foot ulcer (DFU); fetal dermal mesenchymal stem cells (FDMSCs); exosomes from human embryonic stem cells

(ESC-Exos); human amniotic mesenchymal stem cell (hAMSC) ; stem cells from human exfoliated deciduous teeth (SHED); macrophage-derived EVs (MAC-EVs); adipose tissue macrophages derived exosomes (ATM-Exos); exosomes derived from FGF1 pre-conditioned ADSCs (FEXO); selenium-stimulated exosomes (Sel-Exo); eNOS-rich umbilical cord MSC exosomes (UCMSC-exo/eNOS); CHIR99021-pretreated hucMSC-derived exosomes (CHIR-Exos); exosomes derived from atorvastatin-pretreated BMSC (AKT-Exos); streptozotocin (STZ); melatonin-pretreated MSCs-derived exosomes (MT-Exo); S-phase fraction (SPF); proliferation index (PIndex); exosomes derievd from Fe_3O_4 NPs-pretreated MSCs (Exo+NPs); exosomes derievd from placenta-derived MSCs (plaMSC-Ex); fibroblast-derived exosome (FB-EXO); insulin-induced gene 1 (Insig1); Insig1-induced BMSCs exosomes (Insig1-exos): young fibroblast-derived exosome ($exosome^{Young}$); neural stem cells-derived exosomes (NSC-exo); human-induced pluripotent stem cells-derived exosomes (iPSC-exo); keratinocytes derived from induced pluripotent stem cells (iPSCs-KCs); exosomes produced by iPSCs-derived keratinocytes (iPSCs-KCs-Exos); human induced pluripotent stem cells -derived endothelial cells (iPS-ECs); dapagliflozin (DA); DA-loaded nanovesicles derived from iPS-ECs (DA@iPS-EC NV); microvesicle derived from human epidermal keratinocyte adult (HEKa-MV); keratinocyte-derived exosome (KCs-Exo); exosomes derived from autologous dermal fibroblasts (DF-Ex);exosomes derievd from H_2O_2-stimulated HUVECs (H_2O_2-HUVEC-Exos).

Table S2. A comprehensive summary of EV retention, release kinetics, and wound healing efficacy of different biomaterial platforms for wounds treatment.

Type of biomaterial platforms	Components	EV retention (in vivo)	EV Release kinetics	Wound models	Wound healing efficacy	Refs.
Hydrogels	Exo, HBC, OKGM, EPL	Not applicable	• 18.2% released within first 8 h and 66.4% released within 72 h<br>• Continuous release up to 60 h	10-mm-diameter full-thickness skin stretchable wounds in SD rats	• 94.6% wound healing rate by day 9<br>• Almost no obvious scars by day 14<br>• Accelerate wound epithelialization and tissue remodeling<br>• Promote collagen deposition and ECM remodeling<br>• Promote cell proliferation<br>• Promote revascularization	Qin et al. (2021) [72]
	ADSC-exos, ECM	Not applicable	• 95.1±1.4% release over 72 h	10-mm-diameter full-thickness skin wounds on the back of diabetic ICR mice	• 92% wound closure rate on day 14<br>• Promote granulation tissue formation and regenerating collagen at the wound sites<br>• Reduce wound inflammation<br>• Promote angiogenesis of wound<br>• Promote both epithelial cell and endothelial cells proliferation in vivo	Song et al. (2023) [73]
	YBEVs, GelMA	Not applicable	• Continuous release for 14 days	20-mm-diameter full-thickness wound in SD rats	• Approximately 95% wound closure rate by day 14<br>• Promote epidermal and dermal growth<br>• •Promote neovascularization	Wu et al. (2025) [74]

				• Promote functional regeneration of hair follicles or sebaceous glands	
H-exo, SISMA	Not applicable	• 96.33% released by day 21	15-mm-diameter full-thickness skin defect in diabetic rats	• 98% wound closure rate by day 21<br>• Promote re-epithelialization<br>• Promote collagen deposition<br>• Promote angiogenesis<br>• •Inhibit inflammation	Fan et al. (2024) [32]
EXO, HAMA, CSMA, MPBA	Not applicable	• Sustained release for up to 14 days<br>• Glucose-responsive sustained release properties	Full-thickness wounds in diabetic mouse model	• Around 95% wound closure after 12 days treatment<br>• Promote the growth of granulation tissue and collagen remodeling<br>• Enhance angiogenesis<br>• Promote the polarization of M2 macrophages	Yuan et al. (2024) [75]
Exo, GelMA, MXene ($Ti_3C_2T_x$)	Up to 7 days on wounds	• Continuous release up to 15 days	8-mm-diameter full-thickness burn wound on C57BL/6 mice	• Nearly complete wound closure by day 12<br>• 97.49±0.844% healing rate by day 12<br>• Enhance epithelialization rate in burn wounds<br>• Upregulate OLFM4 expression<br>• Reduce ROS levels in epithelium	Liu et al. (2025) [76]
$sEVs^{17\text{-}OE}$, GelMA	Over 5 days on diabetic wound	• Constant and sustained release over 20 days	Full-thickness dorsal skin wounds on DB/db mice	• 97.24±3.47% wound closure rate by day 15<br>• Promote re-epithelialization<br>• Accelerate angiogenesis and collagen deposition at the wound site	Wei et al. (2024) [77]

				• Alleviate senescence and enhance proliferation of skin repair cells	
Exos, OPLL, GOx, CMCS	Not applicable	• pH/glucose dual-responsive release properties<br>• ~80% released over 4 days in glucose-containing PBS solution or under acidic conditions (pH 5.5)	10-mm-diameter skin trauma in diabetic mice	• Complete healing by day 10<br>• Promote collagen deposition<br>• Decrease wound inflammation and regulate macrophage polarization<br>• Promote neovascularization of wound	Yang et al. (2024) [78]
PRP-EXO, GEL, β-CD	Not applicable	• 96.3% released by day 10<br>• Good fit with Zero-order model	10-mm-diameter full-thickness skin wound on diabetes mellitus rats	• 97.05% wound closure rate at day 10<br>• Promote re-epithelialization and collagen deposition<br>• Promote autophagy and inhibiting apoptosis<br>• Promote vascularization	Shu et al. (2023) [79]
pEVs, PAH, Gel	Not applicable	• A burst release during the 2 first hours and then increased over time<br>• 27.5±1.8% released over 17 h	~8-mm-diameter full-thickness skin wound in diabetic rats	• Complete closure of the wound area by day 14<br>• Modulate macrophage polarization<br>• Enhance vascular formation and follicle activation	Back et al. (2024) [80]
Exo, Gelatine, QCS, AC-β-CD	Not applicable	• Continuous release for over 72 h<br>• 60% released within the first 12 h	10-mm-diameter full-thickness skin wound on diabetic mice	• Complete heal by day 14<br>• Promote collagen deposition<br>• Promote macrophages M2 polarization	Cheng at al. (2023) [81]

				• Increase neovascularization	
Exo, OD, GM, QCS	Not applicable	• 89% release on day 9	10-mm-diameter full-thickness defect infected by S. aureus in SD rat	• Complete wound healing by day 14<br>• Eliminate pathogenic bacteria with 95.1±1.6% bacterial inhibition rate<br>• Enhance re-epithelialization process with 161.4±16.2 m thick epithelial layer<br>• 76.7±4.1% collagen deposition rate<br>• Modulate inflammation<br>• Promote angiogenesis	Lv et al. (2024) [82]
PRP-Exo, OSA-G	Not applicable	• Continuous release for 14 days and more than 80% released	10-mm-diameter full-thickness skin injury model in SD rats	• Increase the thickness of epithelial, vascular density, and present regularly arranged collagen	Sun et al. (2025) [83]
ADSC-exo, PEG, Ag^+, M, CNT	At least 10 days in wound site	• Sustained release for 12 days<br>• >80% released by day 12	~10-mm-diameter full-thickness skin wound on diabetic mice	• Virtual wound close by day 10<br>• Enhance cell proliferation<br>• Promote angiogenesis<br>• Reduce inflammation<br>• Reduce ROS production	Zhang et al. (2023) [84]
HucMSC-Exos, HA, AAD, DP7, dopamine	Not applicable	• Quickly released within the first 4 h, followed by slow release at a steady rate<br>• 76.56% released on day 14	Deep second-degree burns with PAO1 bacterial infection in mice	• 93.36±2.30% wound closure rate by day 17<br>• Reduce the number of PAO1 colonies<br>• Produce high proportion of type III collagen<br>• Reduce inflammation and apoptotic cells<br>• Promote neovascularization	Yang et al. (2024) [85]

				• Inhibit scar formation	
SVF-EXO, CSMA, dAMMA	Not applicable	• Nearly 80% released rapidly during the first 10 days, followed by a slow and steady release	10-mm-diameter third-degree burn wound in diabetic mice	• 90% wound closure rate by day 14<br>• Promote neovascularization and re-epithelialization<br>• Exhibit low type I/III collagen ratio<br>• Reduce scar formation	Xiao et al. (2025) [86]
SHED-Exos, ODex, QCSMA-bio	More than 7 days in diabetic wound site	• Not applicable	10-mm-diameter full-thickness wound in diabetic SD rats	• 96.36% wound closure rate by day 14<br>• Accelerate collagen deposition<br>• Reduce inflammation<br>• Exhibit strong angiogenic potential<br>• Restore Treg/Th17 balance	Guo et al. (2025) [87]
Exo, ADA, aPF-127, LL18 peptide	Not applicable	• Sustainably released over 5 days	15-mm-diameter deep partial thickness burns in SD rats	• Complete healing by day 21<br>• Promote re-epithelialization, granulation tissue formation and collagen deposition<br>• Reduce inflammatory response<br>• Facilitate angiogenesis	Vipin et al. (2024) [88]
hMSC-exos, PVA, TPA	Not applicable	• Over 90% released within 72 h in the presence of H2O2<br>• ROS-responsive release properties	15-mm-diameter full-thickness wound in diabetic SD rats	• 96.14±1.20% wound healing rate by day 21<br>• Promote collagen remodeling<br>• Modulate inflammatory responses<br>• Reduce oxidative stress<br>• Enhance angiogenesis<br>• Promote nerve regeneration	Zhang et al. (2025) [89]

Exo, HB-PEG, HA-SH	Not applicable	• Sustained release for 15 days<br>• Over 75% released by day 15	10-mm-diameter full-thickness wound in diabetic rats	• Fast wound closure rate over 21 days<br>• Enhance granular tissue formation and re-epithelialization<br>• Promote collagen deposition<br>• Reverse excessive inflammation<br>• Promote angiogenesis	Wang et al. (2024) [90]
hUCMSC-Exo, AM, CN	Not applicable	• Long-term release capability<br>• 87.67±2.08% released by 7 days	10-mm-diameter full-thickness skin defect in diabetic mice	• Nearly complete wound closure by day 14<br>• Promote epidermal proliferation and collagen deposition<br>• Promote skin appendage regeneration<br>• Accelerate angiogenesis<br>• Promote M2 macrophage polarization	Liu et al. (2025) [91]
Tβ4-Exos, HAMA, PLMA	Over 96 h in wound	• Sustained release	10-mm-diameter full-thickness wound in diabetic mice	• Fast wound closure over 14 days<br>• Promote angiogenesis<br>• Enhance collagen deposition<br>• Regulate macrophage polarization to reduce inflammation	Yu et al. (2025) [92]
M2-Exo, 8P-T, 8P-S	Biodistribution over 6 days by subcutaneous administration	• Tuned release from 6 to 27 days in PBS	8-mm-diameter full-thickness wound in BALB/c mice	• Fast wound closure over 24 days<br>• Enable macrophage reprogramming<br>• Enhance dermal adipogenesis and hair follicle regeneration	Kwak et al. (2022) [93]

	hyBMSC-Exos, Chi-GA, OHA	Over 96 h in diabetic wound	• Not applicable	10-mm-diameter full-thickness wound in db/db mice	• Almost completely released at day 14<br>• Promote re-epithelialization and collagen deposition<br>• Promote the increased transition of M2-to-M2 macrophage polarization<br>• Suppress inflammation<br>• Promote angiogenesis	Shi et al. (2024) [94]
	Exo, Q protein	Rapid local bolus release 24 h after administration and keep retention for 21 days	• A two-stage release profile: an initial burst release within the first 24 h, followed by sustained release over 24-100 h<br>• Gradual network relaxation and matrix erosion<br>• Good match with Gallagher-Corrigan model	10-mm-diameter of excisional wounds on $LepR^{db/db}$ type 2 diabetic mouse dorsal skin	• Median 17 days for closing diabetic wound<br>• 83.7% reduction in pathological time to closure	Subhan et al. (2026) [95]
3D bio-printed hydrogel	M2-Exo, alginate, gelatin, PDA NPs, COL, d-ECM	Not applicable	• Initial burst release within 2 days, followed by a controlled release up to 10 days	Full-thickness skin wound with a diameter of 10 mm on rats	• Great wound contraction by day 14<br>• Reduce scar formation<br>• Promote re-epithelialization<br>• Enhance epidermal thickness<br>• Reduce inflammation	Dutta et al. (2024) [96]

3D-printed scaffold	ApoEVs, GMA, CurCoa	Not applicable	• More than 75% released over 21 days	•Model 1: 20-mm-diameter full-thickness skin wound on SD rat<br>•Model 2: 10-mm-diameter full-thickness skin wound infected by S. aureus on c57 mice	• Model 1: 99.0% wound closure rate on day 15, and accelerating re-epithelialization, thickening granulation tissue, promoting blood vessel formation, and decreasing scar formation<br>• Model 2: Almost 100% healed by day 15, exhibiting antibacterial effect, and promoting collagen deposition, re-epithelialization and blood vessel formation	Jiang et al. (2025) [97]
Microneedle patches	ExoV-ExoL, silk, glycerol	Not applicable	• ~83% release for the patch containing 0wt% glycerol over 6 days<br>• Nearly complete release within 9 days in the presence of bacterial supernatant<br>• Release driven by local enzymatic activity and pH variations, which facilitate microneedle degradation and	8-mm-diameter full-thickness wounds infected by MRSA in diabetic C57BL/6 mice	• Complete epithelialization and full recovery by day 14<br>• Near-complete bacterial eradication<br>• Promote collagen deposition and enhance the orderly alignment of collagen fibers<br>• Reverse immunosuppression and promote M2 macrophage polarization in diabetic infections	Zou et al. (2025) [98]

		enhance exosome diffusion			
EXO@miR-493-5p, GelMA, ZnO, GOx, nano-silver particles	Not applicable	• Over 80% released within 48 h	10-mm-diameter full-thickness wound in STZ-induced diabetic mice	• Achieve complete re-epithelialization and dermal thickening by day 14<br>• Promote M2 polarization<br>• Promote angiogenesis<br>• Attenuate oxidative responses	Chen et al. (2026) [99]
MEs, HAMA, PDA-PVA	Not applicable	• 82.33±3.06% released within 12 h, followed by gradually stabilized release<br>• 84.00±1.73% released after 72 h	9-mm-diameter full-thickness wound in diabetic rat	• 94.99% wound closure area by day 14<br>• Suppress inflammation<br>• Improve collagen deposition<br>• Promote angiogenesis	Zeng et al. (2023) [100]
PLT-Exos, ADMMA	At least 14 days in diabetic wound site	• 82.08% released on day 10 in PBS<br>• Release up to 14 days	20-mm-diameter full-thickness skin defect on the back of diabetic rats	• Over 80% wound healing rate by day 21<br>• Promote re-epithelialization and collagen deposition<br>• Inhibit inflammation<br>• Promote angiogenesis	Cao et al. (2024) [101]
MSC-exos, GelMA, SilMA, Ag NPs	Not applicable	• Rapidly released with 27.09±2.44% released during the first 4 h, followed by gradual release<br>• 87.23±1.96% released after 2 days	10-mm-diameter full-thickness wound in diabetic rats	• Nearly complete re-epithelialization by day 14<br>• Promote angiogenesis<br>• Promote collagen deposition<br>• Inhibit inflammation<br>• Promote angiogenesis	Gan et al. (2022) [102]

EVs$^{T\beta4}$, HA, PEGDA, GelMA	Continuous over 72 h in skin repair cells	• Release over an 11-day period in vitro	10-mm-diameter full-thickness excisional wound in diabetic mice	• High wound closure rate<br>• Regenerate skin appendages<br>• Promote epithelial reformation<br>• Active proliferation of skin repair cells<br>• Accelerate collagen deposition and angiogenesis<br>• Reduce cellular senescence and boost proliferation	Ding et al. (2025) [103]
HUVEC-exo. PEGDA, GelMA	Not applicable	• Sustained release and over 80% released after 10 days	10-mm-diameter full-thickness wound in diabetic mice	• Fast wound closure over 15 days<br>• Promote re-epithelialization<br>• Promote collagen deposition<br>• Promote angiogenesis	Yuan et al. (2022) [104]
ADSC-Exo, ZIF-8, PEGDA, GelMA	Not applicable	• 67.08%±1.33% released by day 10<br>• pH-response release properties<br>• Higher release rate in the acidic environment than in the neutral environment	10-mm-diameter full-thickness wound in diabetic rat	• Almost fully healed by day 16<br>• Promote collagen deposition<br>• Promote angiogenesis<br>• Suppress inflammatory response	Xiang et al. (2025) [105]
KMD@AsExo, PEGDA, GelMA	Not applicable	• 72.4% release rate in the passive release group<br>• 64.3% release rate in the electrically controlled group	6-mm-diameter full-thickness wound infected by S. aureus in Balb/c mice	• Near-complete wound closure by day 12<br>• Decrease bacterial survival<br>• Attenuate inflammatory response<br>• Promote collagen deposition	Zhang et al. (2025) [106]

			• Biphasic release model, characterized by an initial burst phase followed by a sustained release phase			
Electrospun fibrous membrane	BMSC-ABs, PCL	Over 48 h at wound site	• Continuous release over 48 h	8-mm-diameter full-thickness skin wound in C57BL/6 mouse	• Fast wound closure within 10 days<br>• Exert anti-inflammatory and angiogenic effects	Su et al. (2024) [107]
	sEVsHypo-5%, PDA, PCL	Over 12 days on diabetic wound	• Sustained release and 70% released within 14 days	10-mm-diameter full-thickness excisional wound on db/db mice	• Almost complete wound closure on day 14<br>• Enhance neovascularization and ECM deposition	Guo et al. (2026) [108]
	mEXO, PDA, HA, gelatin	Not applicable	• Minimal burst-out effect over time<br>• 16.81% released within 21 days	8-mm-diameter full-thickness wound in C57BL/6 mice	• Fast wound healing rate over 14 days<br>• Promote re-epithelization<br>• Promote angiogenesis	Bui et al. (2024) [109]
Hydrogel/electrospun nanofibers composite scaffold	EXOs, PCL, GT	Not applicable	• 40% released incrementally from day 2 to day 12 and 8% released from day 12 to day 21<br>• Good fit with First-order model	Not applicable	• Not applicable	Chahsetareh et al. (2024) [110]
	EXOs, PCL, Alginate	Not applicable	• ~70% of EXOs released within 7 days	10-mm-diameter full-thickness circular wounds in rat	• 96±3% of wound closure percentage by day 14	Ashrafi et al. (2025) [111]

			• Good fit with Gallagher-Corrigan model<br>• Direct diffusion from alginate network and erosion/dissolution of carrier hydrogel as the dominant release mechanism		• 459.5±62.8 μm of granulation issue thickness on day 14<br>• Up to 65% of the type I collagen formation and up to 98% re-epithelialization	
Microspheres	ABs, GelMA	Up to 21 days retention on diabetic wound	• Slow release within 7 days<br>• On the first day, 27.21% released; from the second to the seventh day, a relative uniform release on each day	10-mm-diameter full-thickness wounds in diabetic SD rats	• 81.19% wound closure rate by day 21<br>• Promote collagen deposition and order arrangement<br>• Modulate inflammation<br>• Promote vascular regeneration	Mao et al. (2024) [112]
	MSC-sEVs, GelMA	Over 7 days in diabetic wounds	• Sustained release and 89%±3% released by day 7	~15-mm-diameter full-thickness wound in diabetic rats	• Almost completely healed by day 28<br>• Enhance M2 macrophage polarization and suppress inflammation<br>• Complete re-epithelialization and a well-organized dermis<br>• Promote collagen deposition	Li et al. (2025) [113]

Cryogel	Lyophilized EVs, PUAO-CPO	Not applicable	• Half-release time: ≈4.2 days<br>• Good match with Higuchi model<br>• Fickian diffusion as the dominant release mechanism	10-mm-diameter full-thickness skin wound in diabetic rats	• By day 21, achieving near complete re-epithelialization with the wound closure rate of 95.71±3.67% in rat diabetic wounds<br>• Promote re-epithelialization, granulation tissue, collagen deposition, and neovascularization, hair follicle formation and overall tissue regeneration<br>• Reduce oxidative stress and inflammation level in diabetic wounds	Shiekh et al. (2026) [114]
Sponges	Exos, CuMOFs, CTMG	Not applicable	• 61.00%±1.61% released by day 7<br>• pH/ROS dual-responsive release behavior<br>• Good fitting with Korsmeyer-Peppas model<br>• A combination of diffusion and swelling-driven release mechanism	10-mm-diameter full-thickness skin defect in SD rat	• 90.10±1.46% of epithelial regeneration rate by day 14<br>• 300.99±1.93 μm of the granulation tissue thickness on day 14<br>• 83.90±4.81% of collagen deposition by day 14<br>• Reduce the expression of inflammatory factors<br>• Promote angiogenesis	Zhou et al. (2025) [115]
	BMSC-Exo, collagen, TSA	About 7 days locally in the wound	• 85% progressively released on day 4 before ultimately	About 10 mm-diameter full-thickness wound in mice	• Fast wound closure over 14 days<br>• Improve angiogenesis and collagen formation<br>• Inhibit the inflammatory response	Wang et al. (2024) [116]

		achieving complete release	

Abbreviations: Hydroxybutyl chitosan (HBC); oxidized konjac glucomannan (OKGM); ε-poly-*L*-lysine (EPL); extracellular matrix (ECM); methyl methacrylate modified small intestine (SISMA); hyaluronic acid methacryloyl (HAMA); chitosan methacryloyl (CSMA); 3-methacrylamidophenylboronic acid (MPBA); gelatin methacrylate (GelMA); oxidized pullulan polysaccharide (OPLL); glucose oxidase (GOx); carboxymethyl chitosan (CMCS); platelet-rich plasma (PRP); gelatin (GEL); β-cyclodextrin (β-CD); platelet extracellular vesicles (pEVs); poly(allylamine) hydrochloride (PAH); quaternary ammonium chitosan (QCS); acrylate β-cyclodextrin (AC-β-CD); oxidized hyaluronic acid (OD); methylacrylylated gelatin (GM); platelet-rich plasma exosomes (PRP-Exo); oxidized sodium alginate-gelatin (OSA-G); polyethylene glycol (PEG); metformin hydrochloride (M); carbon nanotubes (CNTs); hyaluronic acid (HA); antimicrobial peptide (DP7); stromal vascular fraction (SVF); decellularized amniotic membrane modified with methacrylic anhydride (dAMMA); stem cells from human exfoliated deciduous teeth (SHED); oxidized dextran (ODex); methacryloyl- and biotin-modified chitosan (QCSMA-bio); alginate dialdehyde (ADA); aminated Pluronic F127 (aPF-127); polyvinyl alcohol (PVA); N^1-(4-boronobenzyl)-N^3-(4-boronophenyl)-N^1, N^1, N^3, N^3-tetramethylpropane-1,3-diaminium (TPA); hyperbranched polyethylene glycol (HB-PEG); thiol-modified hyaluronic acid (HA-SH); methacrylation-modified acellular dermal matrix (AM); β-cyclodextrin-nature borneol complexes (CN); hyaluronic acid methacryloyl (HAMA); poly-L-lysine methacryloyl (PLMA); gallic acid conjugated chitosan (Chi-GA); oxidized hyaluronic acid (OHA); polydopamine nanoparticles (PDA NPs); collagen (COL); decellularized extracellular matrix (d-ECM); apoptotic extracellular vesicles (ApoEVs); GelMA (GMA); curcumin-containing coacevate (CurCoa); vancomycin & lysostaphin-loaded exosomes (ExoV-ExoL); M2 macrophage-derived exosomes (MEs); platelet-derived exosomes (PLT-Exos); methacrylated acellular dermal matrix (ADMMA); silk fibroin-methacryloyl (SilMA); poly(ethylene glycol) diacrylate (PEGDA); polycaprolactone (PCL); glycyrrhizic acid (GA); black phosphorus (BP); polyurethane (PUAO); calcium peroxide (CPO); copper-based metal-organic frameworks (CuMOFs); trimethylolpropane-modified chitosan derivatives (CTMG); trichostatin A (TSA).

Table S3. A comparative analysis of different biomaterial platforms for delivering EVs in wound healing.

Biomaterial platforms	Advantages	Limitations	Comparative efficacy	Applicable clinical scenarios
Nanocomposites	• Cellular uptake and deep tissue penetration: small-sized nanoparticles are conductive to being taken up by target cells and penetrating deep issues [117]<br>• High specific surface area: High drug loading capacity [118]<br>• Passive targeting: achieving active targeting through the EPR effect or surface modification [119]	• Toxicity concerns: the long-term biological safety of certain inorganic nanoparticles (such as silver, graphene)<br>• Burst release issue: EVs tend to be released in an explosive manner during the initial stage<br>• Easy to aggregate<br>• Poor mechanical support ability	• 80.1±8.8% wound closure rate on the 14th day, promoting epidermal regeneration and hair regrowth [118]	Suitable for wounds that require rapid healing, such as<br>• Infectious wounds<br>• Acute wounds
Hydrogels	• Biomimetic microenvironment: high water content, stimulating ECM, providing a moist healing environment [120]<br>• Responsive release: responding to stimuli such as pH, ROS, enzymes, and achieving intelligent controlled release [121-123]<br>• Different forms: injectable hydrogel, sprayable hydrogel, in-situ forming hydrogel, self-healing hydrogel, hydrogel dressing patch [78, 124-126]<br>• Easy operation: in-situ forming gel and perfectly fitting complex wounds [127]<br>• Absorb exudate, fill dead spaces, and transport cells/drugs [128]	• Large molecular barrier: some gel networks are dense, restricting the release efficiency of EVs<br>• Not resistant to pressure: easy to be compressed and deformed or degraded<br>• Prone to dehydration/expansion	• Nearly 99% wound closure rate at the 14th day, promoting collagen deposition of the skin tissue, enhancing the regeneration of the dermal layer, facilitating angiogenesis [129]<br>• Nearly 100% wound closure rate on the 14th day, promoting granulation tissue formation and collagen deposition, enhancing neurogenesis [130]<br>• 98.2% wound closure rate on the 14th day and ≈100% wound closure rate on the 18th day, accelerating epithelialization, promoting cell proliferation and	Especially suitable for chronic non-healing wounds, such as<br>• Full-thickness skin defects [129]<br>• Diabetic foot ulcers [130]<br>• Infected stretchable wounds [131]

			collagen deposition, reducing inflammation and promoting M2 macrophage polarization [131]	
Microneedle patches	• Physical penetration: breaking through the biofilm barrier and the stratum corneum [106]<br>• Minimally invasive transdermal: bypass the stratum corneum and directly deliver EVs to the dermis [132-134]<br>• Targeted drug delivery: acting directly on the deep layer of the wound or the biofilm [102, 105]	• Low loading capacity: the needle tip is small in size, and the total amount of EVs is limited<br>• Insufficient mechanical strength: the needle tip may break and lead to the residue of microneedles<br>• Ineffective in deep areas: It is difficult to act on deep fascia or muscle layers	• Decreasing the thickness of hypertrophic scar and improving fibroblast distribution and collagen fiber arrangement [135]<br>• Piezoelectric microneedles carrying miR-493-5p: achieve complete re-epithelialization and dermal thickening by day 14, promote M2 polarization and angiogenesis, attenuate oxidative responses [99]	Particularly suitable for superficial chronic wounds, such as<br>• Biofilm-related chronic wounds [136]<br>• Hypertrophic scar [135]
Electrospun fibrous membranes	• Structural mimicry: with similar structure to the ECM, which can guide cell growth, migration and differentiation [109, 137]<br>• High specific surface area and high porosity: possessing breathability, which helps to drainage the wound exudate [138], absorb wound biofluid [139], as well as hemostatic activity [111]	• Insufficient pore size: hinder cell migration<br>• Preparation impact: the high-voltage electric field may damage the activity of EVs<br>• Brittleness and contraction: Mechanical anisotropy<br>• Risk of residual solvent	• 96±3% wound closure rate on the 14th day, promoting granulation tissue formation, re-epithelialization, hair follicle growth, and collagen fiber synthesis [111]<br>• Nearly 100% wound closure rate on the 14th day, promoting collagen deposition, enhancing angiogenesis [109]	Highly suitable for treating skin defects, such as<br>• Burn wounds, especially suitable for superficial burns<br>• Surgical incisions

3D-printed scaffolds	• Personalized customization: capable of precisely constructing a 3D structure that matches the wound surface [140, 141]<br>• Bionic hierarchical structure: simulating the natural skin stratification (dense epidermal layer, loose dermal layer) [96]	• High cost and difficult sterilization: the printing accuracy and the requirements for the bioink are high<br>• Complex printing process: EVs may become inactive during the printing process (due to shear force or ultraviolet irradiation for crosslinking)	• Nearly 100% wound closure rate on the 18th day, enhancing re-epithelization and neovascularization, reducing hyper-granulation, increasing collagen deposition and a shallower depth of inflammation [141]<br>• Nearly 100% wound contraction rate on the 14th day, superior healing ability with thick epidermis, granulation tissue, various glands, and hair follicle growth, reducing inflammation, promoting angiogenesis [96]	Suitable for certain wounds, such as<br>• Complex anatomical areas (such as joints, facial regions)<br>• Large-scale deep defects
Microspheres	• Controlled release: regulate the release of EVs through regulating degradation rate [113]<br>• Injectable property: minimally invasive for filling irregular deep cavities [142]	• Easily to aggregating/drift: likely to disperse in moist wounds<br>• Foreign body reaction: high concentration microspheres may cause granuloma<br>• Migration issue: likely to be fall off from the wound surface and require secondary fixation<br>• No structural support: unable to provide mechanical strength	• Nearly 100% wound closure rate on the 28th day, enhancing M2 polarization and reducing inflammation, promoting re-epithelialization and collagen deposition [113]<br>• Over 80% wound closure rate on the 14th day, promoting re-epithelization, collagen deposition, and ECM remodeling, enhancing angiogenesis [143]	Suitable for injections into deep wounds, such as<br>• Diabetic chronic wounds [143]<br>• Surgical sinus tracts<br>• The base of chronic ulcers

Table S4. A comprehensive comparison of existing wound treatment methods.

Existing wound therapy strategy	Definition and major advantages	Major limitations
Dry dressings (such as cotton gauze)	• Affordability, breathability, durability, safety, lack of allergenicity, ability to manage traumatic bleeding and blood absorbency [144]	• Secondary injury caused by the complex formed by blood clots and the gauze adhering to the wound [145, 146]
Surgical debridement	• A crucial first step in wound care, involving the removal of necrotic tissue, surface microbial biofilms and foreign materials from the wound bed to create an optical healing environment and preserving fresh and well-vascularized tissues [147, 148]	• Invasive and difficult to control the boundary of injury: Excessive debridement may damage healthy tissues (especially tendons, nerves, and blood vessels), while insufficient debridement will be ineffective.<br>• Disordered wound microenvironment: After debridement, there may still be abnormal high expression of MMP and lack of growth factors in the local microenvironment.
PRP	• An autologous blood product (a concentration of platelets in plasma, containing higher-than-baseline levels of platelets, growth factors, and cytokines) [149, 150]<br>• Notable growth factors in PRP including PDGF, VEGF, FGF, IGF, EGF, IL-8, TNF-β [151, 152]<br>• Effective for treating chronic diabetic foot ulcers [153]	• Heterogeneity in preparation and application protocols: Significant variations exist in centrifugation methods, platelet/leukocyte concentrations, activator use and application techniques (injection or topical) [152]
Growth factors	As crucial signaling molecules, greatly influence cell behavior and wound healing [154], and their importance can be demonstrated from two aspects:<br>• Precisely regulating the processes of cell proliferation, differentiation, and migration to ensure that all phases of skin repair proceeds in an orderly and efficient manner<br>• Effectively reducing scar formation by modulating inflammatory	• Short half-life, rapid enzymatic degradation, and poor retention at the wound site [154]<br>• Uncontrolled release behavior and excessive levels may lead to fibrosis and scar formation [155]

	responses, promoting angiogenesis, and remodeling the ECM [155]	
Stem cells	• Isolated from many different sources in the body, such as dental pulp, umbilical cord, bone marrow, skeletal muscle tissue, adipose tissue [156]<br>• Great application potential in the field of regenerative medicine and possess multiple biofunctions, such as promoting angiogenesis, immune regulation, anti-apoptosis, and the recruitment of endogenous stem cells [157, 158]	• Tumorigenicity risk [159, 160]<br>• Immunogenicity and immune reaction: Jeopardize the whole process if the patient's body rejects the transplanted cells; cause cell death is the immune response directed against transplanted allogeneic cell products [161]<br>• Rigorous quality control: Requires stringent GMP guidelines to ensure the safety, efficacy, and scalability of stem cell-based therapies [162]
Skin grafts and flaps	• Skin grafts are the cornerstone of reconstructive surgery, bridging the gap between primary closure and advanced flap-based techniques [163]<br>• Skin flap is a type of reconstruction surgery in which an autologous piece if tissue is rearranged or transferred from one body region to another, and has been used to cover large defects resulting from chronic wounds, burns, trauma, malignancy, and congenital deformities [164]	• Skin graft complications: including infection, graft failure, dehiscence, and bandage-induced complications [165]<br>• Skin flap complications: necrosis, typically of the distal end of the skin flap, is caused by a multitude of vascular and inflammatory factors [164]
HBOT	• Involving the intermittent inhalation of 100% oxygen at elevated atmospheric pressure, which significantly increases oxygen delivery to hypoxic tissues [166]<br>• Enhanced tissue oxygenation promotes angiogenesis, blood supply, fibroblast proliferation, collagen deposition, and directly kills anaerobic bacteria [167, 168]<br>• Recommended as a second-line treatment after progression beyond one month of diabetic foot ulcers [169]	• Narrow application scope: Strict assessment of tissue oxygen partial pressure is required [170]<br>• Requirement for specialized equipment and multiple treatment sessions may present as logistical and resource concerns [168]
NPWT	• A non-invasive therapeutic technique that involves the application of controlled negative pressure to a wound through a specialized	• Cannot replace blood vessel reconstruction [174]<br>• Bulky design, significant operational noise, and frequent need for

	dressing and vacuum-assisted closure system [171]<br>• Remove the wound fluid and create a more favorable environment for wound healing by eliminating infectious substances and exudates, reducing edema, and promoting perfusion and granulation tissue growth [172, 173]	recharging [175]
ES	• Inspired by the pathological mechanism that endogenous electric fields generated from the intact skin toward the wound site can stimulate cell growth and direct the wound healing process [176, 177]<br>• Promote angiogenesis, disrupt biofilms, and induce various responses in the cells involved in wound healing [178]	• Parameter confusion: No standards for waveform, current density, and frequency [177]<br>• ES can lead to excessive ROS accumulation, inducing oxidative stress that inhibits cell activity and may even delay the healing process [179]

Abbreviations: Platelet-rich plasma (PRP); platelet-derived growth factor (PDGF), vascular endothelial growth factor (VEGF), fibroblast growth factor (FGF), insulin-like growth factor (IGF), epidermal growth factor (EGF), interleukin-8 (IL-8), transforming growth factor-β (TNF-β); extracellular matrix (ECM); hyperbaric oxygen therapy (HBOT); negative pressure wound therapy (NPWT); electrical stimulation (ES); reactive oxygen species (ROS).

Table S5. A comparison of different platforms for EV release analysis.

Analysis platforms	Measured contents	Limitations	Refs.
NTA	• Total particle concentration<br>• Size distribution	• Cannot distinguish intact EVs from protein aggregates or scaffold degradation fragments of similar size<br>• Cannot confirm the presence of EV markers or functional cargo	[180]
TEM	• Morphology, size, and integrity of EVs released from biomaterials	• Cannot differentiate EVs that were actively released from those passively desorbed or resulted from scaffold degradation<br>• Time-consuming and only a small field of view can be analyzed, leading to potential sampling bias	[181, 182]
WB	• Specific EVs marker proteins (e.g., CD63, CD81, TSG101)<br>• Intensity of the target protein band in the release medium, quantified by densitometry	• Signal intensity reflects protein amount, not EV particle concentration<br>• Leading to false-positive signals and inaccurate quantification due to other proteins that cross-react with the primary antibody used in WB	[121, 133, 135]
FCM	• Surface markers of EVs (such as CD9, CD63, CD81)	• Fluorescently conjugated antibodies or lipophilic dyes may dissociate from EVs membrane during incubation, leading to false positive signals (free dyes/antibodies are counted as EVs) or false negative signals (EVs lose fluorescence)	[73, 130]
Fluorescence labeling	• Cell internalization (in vitro tracking)<br>• Tissue retention (in vivo tracking)	• Fluorescent dyes or antibodies may dissociate from EVs during release (e.g., due to pH changes, enzyme degradation in the wound microenvironment), leading to false-positive fluorescence signals that overestimate the EVs release amount	[95, 183, 184]
BCA Protein Assay Kit	• Total protein quantification<br>• EV release kinetics	• Non-specific and can measure all protein (e.g., protein leached from the biomaterial itself or serum proteins in the culture medium) in release medium	[185]

Abbreviations: Nanoparticle Tracking Analysis (NTA); Transmission Electron Microscopy (TEM); Western Blot (WB); Flow Cytometer (FCM); Bicinchoninic Acid (BCA).

Table S6. Quality control framework for biomaterials carrying EVs.

Category	Key elements	Main recommendations
Production materials	• Production cells<br>• Raw and auxiliary materials (natural EVs)<br>• Raw and auxiliary materials (engineered EVs)<br>• Consumable, packaging materials	• Refer to "Guidelines for Cell and Gene Therapy" products<br>• Cells, culture media, additives<br>• Plasmids, bacterial and viral strains, auxiliary cells, viruses, small molecule drugs, bioactive molecules<br>• Ensure traceability and controllability<br>• Require clear sources<br>• Prefer materials of non-animal origin<br>• Refer to "Guidelines for Cell and Gene Therapy"
Key process parameters and process control	• Critical process steps, in-process control (IPC), intermediate product inspection (if applicable)	• Use the "quality from design" concept to define critical process parameters (CPPs) and IPCs to ensure EV consistency and quality<br>• Must include process performance indicators, microbial safety tests, and appropriate process control
Characterization of EVs	• Identity verification<br>• Particle size and concentration<br>• Morphology and structure<br>• Zeta potential<br>• Component analysis<br>• Key drug components<br>• Purity and content	• Detect surface markers (e.g., CD9, CD63, CD81, AL1X), short tandem repeat sequences (STR)<br>• Engineered EVs require additional identification of target molecules<br>• Size distribution and quantity of EV particles<br>• Preliminary assessment of integrity and purity<br>• Surface charge analysis<br>• Lipids, proteins, nucleic acids (including omics analysis)<br>• Need to exclude interference from contaminants during separation and purification<br>• Define and detect functional molecules (such as miRNA, proteins)<br>• Determine the purity based on membrane integrity and positive rate of markers<br>• Engineered EVs need to evaluate encapsulation rate, drug loading, and

		positive rate
	• Impurity	• Non-membrane particles, free molecules, non-positive particles, EV without drugs (based on product functionality defined as impurities)
	• Bioactivity	• Evaluate at the molecular, cellular, and animal levels<br>• Natural EVs assess immunomodulation, anti-apoptosis, pro-angiogenic activities<br>• Engineered EVs assess uptake efficiency, therapeutic molecule functions
Quality attributes	• General physicochemical properties	• Assess pH, osmotic pressure, visible impurities, and microbial safety-related attributes
	• General considerations	• Establish release and stability specifications for active substance and final formulation (if applicable)<br>• The testing items should include identity, concentration, particle size distribution, morphology, bioactivity, purity, impurities, microbial safety, and general properties<br>• The analytical methods must undergo comprehensive validation
	• Acceptable limits	• Based on non-clinical and clinical study data<br>• Use statistical methods to set the batch release standards by combining with stability data
	• Unique properties of engineered EVs	• Encapsulation efficiency<br>• Drug loading capacity<br>• Positive marker rate
	• Reference standards	• Encourage the establishment of reference standards
Stability	• Storage conditions<br>• Packaging materials<br>• Transportation	• Support the determination of validity period<br>• Evaluate the stability of intermediate products
Process validation	• Batch-to-batch consistency<br>• Quality controllability	• Validate using at least three consecutive batches<br>• Ensure process control and reproducibility

References

[1] J. Ma, Z. Zhang, Y. Wang, H. Shen, Investigation of miR-126-3p loaded on adipose stem cell-derived exosomes for wound healing of full-thickness skin defects, Exp Dermatol 31(3) (2021) 362-374.

[2] X. Li, Y. Chang, W. Shen, G. Huang, N. Hu, H. Lv, M. Jin, miR-138 from ADSC Exo accelerates wound healing by targeting SIRT1/PTEN pathway to promote angiogenesis and fibrosis, Cell Signal 111 (2023).

[3] X. Hao, Y. Guo, X. Yu, L. He, Y. He, M. Shu, Exosomes from adipose-derived stem cells overexpressing microRNA-671–3p promote fat graft angiogenesis and adipogenic differentiation, Tissue Cell 91 (2024).

[4] J. Li, C. Wei, Y. Yang, Z. Gao, Z. Guo, F. Qi, Apoptotic bodies extracted from adipose mesenchymal stem cells carry microRNA-21–5p to induce M2 polarization of macrophages and augment skin wound healing by targeting KLF6, Burns 48(8) (2022) 1893-1908.

[5] W. Li, H. Zhang, P. Bai, H. Wang, F. Hou, Z. Zhou, W. Li, ADMSC-derived exosomes promote diabetic wound healing by inhibiting neutrophil extracellular traps formation through delivering miR-92a-1-5p, Arch. Biochem. Biophys. 778 (2026).

[6] L. Pi, L. Yang, B.-R. Fang, X.-X. Meng, L. Qian, Exosomal microRNA-125a-3p from human adipose-derived mesenchymal stem cells promotes angiogenesis of wound healing through inhibiting PTEN, Mol Cell Biochem 477(1) (2021) 115-127.

[7] S. Ren, J. Chen, J. Guo, Y. Liu, H. Xiong, B. Jing, X. Yang, G. Li, Y. Kang, C. Wang, X. Xu, Z. Liu, M. Zhang, K. Xiang, C. Li, Q. Li, H.-G. Machens, Z. Chen, Exosomes from Adipose Stem Cells Promote Diabetic Wound Healing through the eHSP90/LRP1/AKT Axis, Cells 11(20) (2022).

[8] Q. Liang, D. Zhou, X. Ge, P. Song, W. Chu, J. Xu, Y. Shen, Exosomes from adipose-derived mesenchymal stem cell improve diabetic wound healing and inhibit fibrosis via miR-128-1-5p/TGF-β1/Smad axis, Mol. Cell Endocrinol. 588 (2024).

[9] Z. Qiao, X. Wang, H. Zhao, Y. Deng, W. Zeng, J. Wu, Y. Chen, Research on the TSPAN6 regulating the secretion of ADSCs-Exos through syntenin-1 and promoting wound healing, Stem Cell Res. Ther. 15(1) (2024).

[10] R. Shi, P. Jia, S. Zhao, H. Yuan, J. Shi, H. Zhao, Upregulation of circ-IGF1R increased therapeutic effect of hypoxia-pretreated ADSC-derived extracellular vesicle by regulating miR-503-5p/HK2/VEGFA axis, J Cell Mol Med 28(13) (2024).

[11] B. Li, L. Qian, L. Pi, X. Meng, A therapeutic role of exosomal lncRNA H19 from adipose mesenchymal stem cells in cutaneous wound healing by triggering macrophage M2 polarization, Cytokine 165 (2023).

[12] J. Xiong, Z. Liu, M. Wu, M. Sun, Y. Xia, Y. Wang, Comparison of Proangiogenic Effects of Adipose-Derived Stem Cells and Foreskin Fibroblast Exosomes on Artificial Dermis Prefabricated Flaps, Stem Cells Int 2020 (2020) 1-14.

[13] L. Pi, L. Yang, B.-R. Fang, X.-X. Meng, L. Qian, LncRNA MALAT1 from Human Adipose-Derived Stem Cell Exosomes accelerates Wound Healing Via miR-378a/FGF2 Axis, Regen Med 17(9) (2022) 627-641.

[14] J. Qiu, C. Shu, X. Li, C. Ye, W.-C. Zhang, Exosomes from linc00511-overexpressing ADSCs accelerates angiogenesis in diabetic foot ulcers healing by suppressing PAQR3-induced Twist1 degradation, Diabetes Res Clin Pr 180 (2021).

[15] H. Yu, Y. Wu, B. Zhang, M. Xiong, Y. Yi, Q. Zhang, M. Wu, Exosomes Derived from E2F1–/–

Adipose-Derived Stem Cells Promote Skin Wound Healing via miR-130b-5p/TGFBR3 Axis, Int J Nanomed Volume 18 (2023) 6275-6292.
[16] L. Qian, L. Pi, B.-R. Fang, X.-X. Meng, Adipose mesenchymal stem cell-derived exosomes accelerate skin wound healing via the lncRNA H19/miR-19b/SOX9 axis, Lab Invest 101(9) (2021) 1254-1266.
[17] D. Che, X. Xiang, J. Xie, Z. Chen, Q. Bao, D. Cao, Exosomes Derived from Adipose Stem Cells Enhance Angiogenesis in Diabetic Wound Via miR-146a-5p/JAZF1 Axis, Stem Cell Rev Rep 20(4) (2024) 1026-1039.
[18] X. Zhang, X. Jiang, H. Deng, G. Yu, N. Yang, A. Al Mamun, F. Lian, T. Chen, H. Zhang, Y. Lai, J. Huang, S. Xu, F. Cai, X. Li, K. Zhou, J. Xiao, Engineering exosomes from fibroblast growth factor 1 pre-conditioned adipose-derived stem cells promote ischemic skin flaps survival by activating autophagy, Mater. Today Bio 29 (2024).
[19] M. Wu, J. Tu, J. Huang, H. Wen, Y. Zeng, Y. Lu, Exosomal IRF1-loaded rat adipose-derived stem cell sheet contributes to wound healing in the diabetic foot ulcers, Mol. Med. 29(1) (2023).
[20] Y. Bo, L. Yang, B. Liu, G. Tian, C. Li, L. Zhang, Y. Yan, Exosomes from human induced pluripotent stem cells-derived keratinocytes accelerate burn wound healing through miR-762 mediated promotion of keratinocytes and endothelial cells migration, J Nanobiotechnol 20(1) (2022).
[21] M. Ye, Q. Ni, H. Qi, X. Qian, J. Chen, X. Guo, M. Li, Y. Zhao, G. Xue, H. Deng, L. Zhang, Exosomes Derived from Human Induced Pluripotent Stem Cells-Endothelia Cells Promotes Postnatal Angiogenesis in Mice Bearing Ischemic Limbs, Int J Biol Sci 15(1) (2019) 158-168.
[22] B. Cheng, X. Song, L. Yin, J. Lin, Z. Liu, Y. Zhu, H. Wu, HMOX1-overexpressing mesenchymal stem cell-derived exosomes facilitate diabetic wound healing by promoting angiogenesis and fibroblast function, Biochem Bioph Res Co 690 (2024).
[23] X. Zhou, C. Ye, L. Jiang, X. Zhu, F. Zhou, M. Xia, Y. Chen, The bone mesenchymal stem cell-derived exosomal miR-146a-5p promotes diabetic wound healing in mice via macrophage M1/M2 polarization, Mol Cell Endocrinol 579 (2024).
[24] B. Li, S. Luan, J. Chen, Y. Zhou, T. Wang, Z. Li, Y. Fu, A. Zhai, C. Bi, The MSC-Derived Exosomal lncRNA H19 Promotes Wound Healing in Diabetic Foot Ulcers by Upregulating PTEN via MicroRNA-152-3p, Mol Ther Nucl Acids 19 (2020) 814-826.
[25] Z. Wang, H. Xu, B. Xue, L. Liu, Y. Tang, Z. Wang, K. Yao, MSC-derived exosomal circMYO9B accelerates diabetic wound healing by promoting angiogenesis through the hnRNPU/CBL/KDM1A/VEGFA axis, Commun. Biol. 7(1) (2024).
[26] P. Gondaliya, A.A. Sayyed, P. Bhat, M. Mali, N. Arya, A. Khairnar, K. Kalia, Mesenchymal Stem Cell-Derived Exosomes Loaded with miR-155 Inhibitor Ameliorate Diabetic Wound Healing, Mol. Pharmaceutics 19(5) (2022) 1294-1308.
[27] C. Deng, K. Dong, Y. Liu, K. Chen, C. Min, Z. Cao, P. Wu, G. Luo, G. Cheng, L. Qing, J. Tang, Hypoxic mesenchymal stem cell-derived exosomes promote the survival of skin flaps after ischaemia–reperfusion injury via mTOR/ULK1/FUNDC1 pathways, J Nanobiotechnol 21(1) (2023).
[28] X. Gu, T. Li, X. Yin, P. Zhai, D. Jiang, D. Sun, H. Yan, B. Wang, Exosomes Derived from Metformin-Pretreated BMSCs Accelerate Diabetic Wound Repair by Promoting Angiogenesis Via the LINC-PINT/miR-139-3p/FOXC2 Axis, Stem Cell Rev Rep (2025).
[29] L. Zheng, H. Song, Y. Li, H. Li, G. Lin, Z. Cai, Insulin-Induced Gene 1-Enhance Secretion of BMSC Exosome Enriched in miR-132–3p Promoting Wound Healing in Diabetic Mice, Mol.

Pharmaceutics 21(9) (2024) 4372-4385.
[30] Z.Y. Qiu, W.c. Xu, Z.H. Liang, Bone marrow mesenchymal stem cell-derived exosomal miR-221-3p promotes angiogenesis and wound healing in diabetes via the downregulation of forkhead box P1, Diabetic Med 41(9) (2024).
[31] X. He, Z. Dong, Y. Cao, H. Wang, S. Liu, L. Liao, Y. Jin, L. Yuan, B. Li, MSC-Derived Exosome Promotes M2 Polarization and Enhances Cutaneous Wound Healing, Stem Cells Int 2019 (2019) 1-16.
[32] M. Fan, X. Zhang, Y. Jiang, J. Pi, J. Zhang, Y. Zhang, F. Xing, H. Xie, Exosomes from hypoxic urine-derived stem cells facilitate healing of diabetic wound by targeting SERPINE1 through miR-486-5p, Biomaterials 314 (2025).
[33] C.-Y. Chen, S.-S. Rao, L. Ren, X.-K. Hu, Y.-J. Tan, Y. Hu, J. Luo, Y.-W. Liu, H. Yin, J. Huang, J. Cao, Z.-X. Wang, Z.-Z. Liu, H.-M. Liu, S.-Y. Tang, R. Xu, H. Xie, Exosomal DMBT1 from human urine-derived stem cells facilitates diabetic wound repair by promoting angiogenesis, Theranostics 8(6) (2018) 1607-1623.
[34] Y. Zhang, Y. Pan, Y. Liu, X. Li, L. Tang, M. Duan, J. Li, G. Zhang, Exosomes derived from human umbilical cord blood mesenchymal stem cells stimulate regenerative wound healing via transforming growth factor-β receptor inhibition, Stem Cell Res. Ther. 12(1) (2021).
[35] C. Gong, C. Xia, L. Liu, Exosomes derived from epidermal growth factor-like domain protein 6-preconditioned mesenchymal stem cells for diabetic wound healing, Regen. Ther. 26 (2024) 932-940.
[36] Q. Li, L. Guo, J. Wang, S. Tao, P. Jin, Exosomes derived from Nr-CWS pretreated MSCs facilitate diabetic wound healing by promoting angiogenesis via the circIARS1/miR-4782-5p/VEGFA axis, Chin. J. Nat. Med. 21(3) (2023) 172-184.
[37] J. Liu, Z. Yan, F. Yang, Y. Huang, Y. Yu, L. Zhou, Z. Sun, D. Cui, Y. Yan, Exosomes Derived from Human Umbilical Cord Mesenchymal Stem Cells Accelerate Cutaneous Wound Healing by Enhancing Angiogenesis through Delivering Angiopoietin-2, Stem Cell Rev Rep 17(2) (2020) 305-317.
[38] Q. Wei, Y. Wang, K. Ma, Q. Li, B. Li, W. Hu, X. Fu, C. Zhang, Extracellular Vesicles from Human Umbilical Cord Mesenchymal Stem Cells Facilitate Diabetic Wound Healing Through MiR-17-5p-mediated Enhancement of Angiogenesis, Stem Cell Rev Rep 18(3) (2021) 1025-1040.
[39] M. Wang, D. Yang, L. Li, P. Wu, Y. Sun, X. Zhang, C. Ji, W. Xu, H. Qian, H. Shi, A Dual Role of Mesenchymal Stem Cell Derived Small Extracellular Vesicles on TRPC6 Protein and Mitochondria to Promote Diabetic Wound Healing, ACS Nano 18(6) (2024) 4871-4885.
[40] X. Zhao, L. Fu, H. Zou, Y. He, Y. Pan, L. Ye, Y. Huang, W. Fan, J. Zhang, Y. Ma, J. Chen, M. Zhu, C. Zhang, Y. Cai, X. Mou, Optogenetic engineered umbilical cord MSC-derived exosomes for remodeling of the immune microenvironment in diabetic wounds and the promotion of tissue repair, J Nanobiotechnol 21(1) (2023).
[41] X. Li, L. Liu, J. Yang, Y. Yu, J. Chai, L. Wang, L. Ma, H. Yin, Exosome Derived From Human Umbilical Cord Mesenchymal Stem Cell Mediates MiR-181c Attenuating Burn-induced Excessive Inflammation, EBioMedicine 8 (2016) 72-82.
[42] C. Xiu, H. Zheng, M. Jiang, J. Li, Y. Zhou, L. Mu, W. Liu, MSCs-Derived miR-150-5p-Expressing Exosomes Promote Skin Wound Healing by Activating PI3K/AKT Pathway through PTEN, Int J Stem Cells 15(4) (2022) 359-371.
[43] W. Nie, X. Huang, L. Zhao, T. Wang, D. Zhang, T. Xu, L. Du, Y. Li, W. Zhang, F. Xiao, L.

Wang, Exosomal miR-17–92 derived from human mesenchymal stem cells promotes wound healing by enhancing angiogenesis and inhibiting endothelial cell ferroptosis, Tissue Cell 83 (2023).
[44] X.-F. Zhang, T. Wang, Z.-X. Wang, K.-P. Huang, Y.-W. Zhang, G.-L. Wang, H.-J. Zhang, Z.-H. Chen, C.-Y. Wang, J.-X. Zhang, H. Wang, Hypoxic ucMSC-secreted exosomal miR-125b promotes endothelial cell survival and migration during wound healing by targeting TP53INP1, Mol Ther Nucl Acids 26 (2021) 347-359.
[45] X. Wang, Y. Jiao, Y. Pan, L. Zhang, H. Gong, Y. Qi, M. Wang, H. Gong, M. Shao, X. Wang, D. Jiang, Fetal Dermal Mesenchymal Stem Cell-Derived Exosomes Accelerate Cutaneous Wound Healing by Activating Notch Signaling, Stem Cells Int 2019 (2019) 1-11.
[46] B. Chen, Y. Sun, J. Zhang, Q. Zhu, Y. Yang, X. Niu, Z. Deng, Q. Li, Y. Wang, Human embryonic stem cell-derived exosomes promote pressure ulcer healing in aged mice by rejuvenating senescent endothelial cells, Stem Cell Res. Ther. 10(1) (2019).
[47] C. Peng, H. Xu, Q. Zhuang, J. Liu, Y. Ding, Q. Tang, Z. Wang, K. Yao, Placenta-derived mesenchymal stem cells promote diabetic wound healing via exosomal protein interaction networks, Wound Repair Regen 32(5) (2024) 638-651.
[48] S. Fu, H. Zhang, X. Li, Q. Zhang, C. Guo, K. Qiu, J. Feng, X. Liu, D. Liu, Exosomes Derived from Human Amniotic Mesenchymal Stem Cells Facilitate Diabetic Wound Healing by Angiogenesis and Enrich Multiple lncRNAs, Tissue Eng Regen Med 20(2) (2023) 295-308.
[49] S. Gao, T. Chen, Y. Hao, F. Zhang, X. Tang, D. Wang, Z. Wei, J. Qi, Exosomal miR-135a derived from human amnion mesenchymal stem cells promotes cutaneous wound healing in rats and fibroblast migration by directly inhibiting LATS2 expression, Stem Cell Res. Ther. 11(1) (2020).
[50] Y. Xie, L. Yu, Z. Cheng, Y. Peng, Z. Cao, B. Chen, Y. Duan, Y. Wang, SHED-derived exosomes promote LPS-induced wound healing with less itching by stimulating macrophage autophagy, J Nanobiotechnol 20(1) (2022).
[51] Z. Zhou, J. Zheng, D. Lin, R. Xu, Y. Chen, X. Hu, Exosomes derived from dental pulp stem cells accelerate cutaneous wound healing by enhancing angiogenesis via the Cdc42/p38 MAPK pathway, Int J Mol Med 50(6) (2022).
[52] H. Xu, H. Yang, Z. Wang, Q. Tang, X. Cao, C. Chen, Y. Dong, Z. Xu, D. Lv, Y. Rong, M. Chen, B. Tang, W. Deng, J. Zhu, Z. Hu, Epidermal Stem Cell Derived Exosomes Alleviate Excessive Autophagy Induced Endothelial Cell Apoptosis by Delivering miR200b-3p to Diabetic Wounds, J Invest Dermatol 144(5) (2024) 1134-1147.e2.
[53] P. Wang, G. Theocharidis, I.S. Vlachos, K. Kounas, A. Lobao, B. Shu, B. Wu, J. Xie, Z. Hu, S. Qi, B. Tang, J. Zhu, A. Veves, Exosomes Derived from Epidermal Stem Cells Improve Diabetic Wound Healing, J Invest Dermatol 142(9) (2022) 2508-2517.e13.
[54] H. Yang, H. Xu, Z. Wang, X. Li, P. Wang, X. Cao, Z. Xu, D. Lv, Y. Rong, M. Chen, B. Tang, Z. Hu, W. Deng, J. Zhu, Analysis of miR-203a-3p/SOCS3-mediated induction of M2 macrophage polarization to promote diabetic wound healing based on epidermal stem cell-derived exosomes, Diabetes Res Clin Pr 197 (2023).
[55] R. Lou, J. Chen, F. Zhou, T. Zhang, X. Chen, C. Wang, B. Guo, L. Lin, Exosomal miRNA-155-5p from M1-polarized macrophages suppresses angiogenesis by targeting GDF6 to interrupt diabetic wound healing, Mol Ther Nucl Acids 34 (2023).
[56] G. Luo, Z. Zhou, Z. Cao, C. Huang, C. Li, X. Li, C. Deng, P. Wu, Z. Yang, J. Tang, L. Qing, M2 macrophage-derived exosomes induce angiogenesis and increase skin flap survival through HIF1AN/HIF-1α/VEGFA control, Arch. Biochem. Biophys. 751 (2024).

[57] Y. Deng, J. Xie, J. Xiao, X. Huang, Z. Cao, Gelatin methacryloyl hydrogel encapsulating molybdenum-inspired macrophage-derived exosomes accelerates wound healing via immune regulation and angiogenesis, Int J Biol Macromol 291 (2025).
[58] P. Gangadaran, R.L. Rajendran, J.M. Oh, C.M. Hong, S.Y. Jeong, S.-W. Lee, J. Lee, B.-C. Ahn, Extracellular vesicles derived from macrophage promote angiogenesis In vitro and accelerate new vasculature formation In vivo, Exp. Cell Res. 394(2) (2020).
[59] M. Li, T. Wang, H. Tian, G. Wei, L. Zhao, Y. Shi, Macrophage-derived exosomes accelerate wound healing through their anti-inflammation effects in a diabetic rat model, Artif. Cell. Nanomed. B. 47(1) (2019) 3793-3803.
[60] W. Xia, Y. Liu, X. Jiang, M. Li, S. zheng, Z. Zhang, X. Huang, S. Luo, Y. Khoong, M. Hou, T. Zan, Lean adipose tissue macrophage derived exosome confers immunoregulation to improve wound healing in diabetes, J Nanobiotechnol 21(1) (2023).
[61] X. Han, P. Wu, L. Li, H.M. Sahal, C. Ji, J. Zhang, Y. Wang, Q. Wang, H. Qian, H. Shi, W. Xu, Exosomes derived from autologous dermal fibroblasts promote diabetic cutaneous wound healing through the Akt/β-catenin pathway, Cell Cycle 20(5-6) (2021) 616-629.
[62] W. Xia, M. Li, X. Jiang, X. Huang, S. Gu, J. Ye, L. Zhu, M. Hou, T. Zan, Young fibroblast-derived exosomal microRNA-125b transfers beneficial effects on aged cutaneous wound healing, J Nanobiotechnol 20(1) (2022).
[63] G. Yu, Y. Chen, N. Yang, H. Zhang, X. Zhang, Y. Geng, J. Zhao, Z. Chen, C. Dong, L. Lin, J. Qi, X. Zhang, X. Jiang, W. Gao, Y. Cai, X. Wang, J. Ding, J. Xiao, K. Zhou, Apoptotic Bodies Derived from Fibroblast-Like Cells in Subcutaneous Connective Tissue Inhibit Ferroptosis in Ischaemic Flaps via the miR-339-5p/KEAP1/Nrf2 Axis, Adv. Sci. 11(24) (2024).
[64] Q. Li, H. Zhao, W. Chen, P. Huang, J. Bi, Human keratinocyte-derived microvesicle miRNA-21 promotes skin wound healing in diabetic rats through facilitating fibroblast function and angiogenesis, Int J Biochem Cell B 114 (2019).
[65] A. Glady, A. Vandebroek, M. Yasui, Human keratinocyte-derived extracellular vesicles activate the MAPKinase pathway and promote cell migration and proliferation in vitro, Inflamm. Regen. 41(1) (2021).
[66] L. Kuang, C. Zhang, B. Li, H. Deng, R. Chen, G. Li, Human Keratinocyte-Derived Exosomal MALAT1 Promotes Diabetic Wound Healing by Upregulating MFGE8 via microRNA-1914-3p, Int J Nanomed Volume 18 (2023) 949-970.
[67] X. Li, C. Jiang, J. Zhao, Human endothelial progenitor cells-derived exosomes accelerate cutaneous wound healing in diabetic rats by promoting endothelial function, J Diabetes Complicat 30(6) (2016) 986-992.
[68] X. Zou, Q. Yang, Y. Xiao, L. Zhang, H. Xiao, H. Lei, X. Zhang, X. Bai, W. Xiong, Endothelial Progenitor-Cell-Derived Exosomes Induced by Astragaloside IV Accelerate Type I Diabetic-wound Healing via the PI3K/AKT/mTOR Pathway in Rats, Front Biosci-Landmark 28(11) (2023).
[69] L. Guo, Y. Chen, X. Feng, D. Sun, J. Sun, S. Mou, K. Zhao, R. An, Oxidative stress-induced endothelial cells-derived exosomes accelerate skin flap survival through Lnc NEAT1-mediated promotion of endothelial progenitor cell function, Stem Cell Res. Ther. 13(1) (2022).
[70] J. Zhang, C. Chen, B. Hu, X. Niu, X. Liu, G. Zhang, C. Zhang, Q. Li, Y. Wang, Exosomes Derived from Human Endothelial Progenitor Cells Accelerate Cutaneous Wound Healing by Promoting Angiogenesis Through Erk1/2 Signaling, Int J Biol Sci 12(12) (2016) 1472-1487.
[71] J. Xu, S. Bai, Y. Cao, L. Liu, Y. Fang, J. Du, L. Luo, M. Chen, B. Shen, Q. Zhang, miRNA-

221-3p in Endothelial Progenitor Cell-Derived Exosomes Accelerates Skin Wound Healing in Diabetic Mice, Diabet Metab Synd OB Volume 13 (2020) 1259-1270.
[72] D. Qin, A. Zhang, N. Wang, Y. Yao, X. Chen, Y. Liu, Hydroxybutyl chitosan/ oxidized glucomannan self-healing hydrogels as BMSCs-derived exosomes carriers for advanced stretchable wounds, Appl. Mater. Today 26 (2022).
[73] Y. Song, Y. You, X. Xu, J. Lu, X. Huang, J. Zhang, L. Zhu, J. Hu, X. Wu, X. Xu, W. Tan, Y. Du, Adipose-Derived Mesenchymal Stem Cell-Derived Exosomes Biopotentiated Extracellular Matrix Hydrogels Accelerate Diabetic Wound Healing and Skin Regeneration, Adv. Sci. 10(30) (2023).
[74] Y. Wu, J. Mao, Y. Zhou, G. Hong, H. Wu, Z. Hu, X. Huang, J. Shi, Z. Xie, Y. Lan, Youthful Brain-Derived Extracellular Vesicle-Loaded GelMA Hydrogel Promotes Scarless Wound Healing in Aged Skin by Modulating Senescence and Mitochondrial Function, Research 8 (2025) 1-14.
[75] W. Yuan, C. Huang, W. Deng, J. Lai, J. Chen, J. Jie, Y. Wu, T. You, L.-P. Wu, Hyaluronic acid methacryloyl/chitosan methacryloyl/3-methacrylamidophenylboronic acid multifunctional hydrogel loading exosome for diabetic wound healing, Int J Biol Macromol 280 (2024).
[76] W. Liu, H. Huang, F. Shu, Y. Liu, J. Lin, L. Yang, W. Zhang, L. Jiang, T. Liu, C. Xie, L. Li, Y. He, S. Xiao, Y. Zheng, Z. Xia, AntagomiR-192-5p-engineered exosomes encapsulated in MXene-modified GelMA hydrogel facilitated epithelization of burn wounds by targeting OLFM4, Bioact. Mater. 52 (2025) 318-337.
[77] Q. Wei, J. Su, S. Meng, Y. Wang, K. Ma, B. Li, Z. Chu, Q. Huang, W. Hu, Z. Wang, L. Tian, X. Liu, T. Li, X. Fu, C. Zhang, MiR-17-5p-engineered sEVs Encapsulated in GelMA Hydrogel Facilitated Diabetic Wound Healing by Targeting PTEN and p21, Adv. Sci. 11(13) (2024).
[78] P. Yang, Y. Ju, N. Shen, S. Zhu, J. He, L. Yang, J. Lei, X. He, W. Shao, L. Lei, B. Fang, Exos-Loaded Gox-Modified Smart-Response Self-Healing Hydrogel Improves the Microenvironment and Promotes Wound Healing in Diabetic Wounds, Adv. Healthcare Mater. (2024).
[79] Q.-H. Shu, R.-T. Zuo, M. Chu, J.-J. Shi, Q.-F. Ke, J.-J. Guan, Y.-P. Guo, Fiber-reinforced gelatin/β-cyclodextrin hydrogels loaded with platelet-rich plasma-derived exosomes for diabetic wound healing, Biomater. Adv. 154 (2023).
[80] F. Back, A. Barras, A. Nyam-Erdene, J.C. Yang, S. Melinte, J. Rumipamba, T. Burnouf, R. Boukherroub, S. Szunerits, E.Y. Chuang, Platelet Extracellular Vesicles Loaded Gelatine Hydrogels for Wound Care, Adv. Healthcare Mater. 14(1) (2024).
[81] P. Cheng, X. Xie, L. Hu, W. Zhou, B. Mi, Y. Xiong, H. Xue, K. Zhang, Y. Zhang, Y. Hu, L. Chen, K. Zha, B. Lv, Z. Lin, C. Lin, G. Dai, Y. Hu, T. Yu, H. Hu, G. Liu, Y. Zhang, Hypoxia endothelial cells-derived exosomes facilitate diabetic wound healing through improving endothelial cell function and promoting M2 macrophages polarization, Bioact. Mater. 33 (2024) 157-173.
[82] Y. Lv, L. Li, J. Zhang, J. Li, F. Cai, Y. Huang, X. Li, Y. Zheng, X. Shi, J. Yang, Visible-Light Cross-Linkable Multifunctional Hydrogels Loaded with Exosomes Facilitate Full-Thickness Skin Defect Wound Healing through Participating in the Entire Healing Process, ACS Appl. Mater. Interfaces 16(20) (2024) 25923-25937.
[83] D. Sun, H. Yu, H. Shen, Y. Wang, T. Su, H. Guo, X. Xing, W. Wang, L. Wei, W. Gao, Y. Yao, B. Xiu, J. Wang, X. Zhang, Y. Guo, Sodium alginate hydrogel platelet rich plasma exosome loaded for synergistic hemostasis, Int J Biol Macromol 311 (2025).
[84] Y. Zhang, M. Li, Y. Wang, F. Han, K. Shen, L. Luo, Y. Li, Y. Jia, J. Zhang, W. Cai, K. Wang, M. Zhao, J. Wang, X. Gao, C. Tian, B. Guo, D. Hu, Exosome/metformin-loaded self-healing conductive hydrogel rescues microvascular dysfunction and promotes chronic diabetic wound

healing by inhibiting mitochondrial fission, Bioact. Mater. 26 (2023) 323-336.
[85] Y. Yang, J. Zhang, S. Wu, Y. Deng, S. Wang, L. Xie, X. Li, L. Yang, Exosome/antimicrobial peptide laden hydrogel wound dressings promote scarless wound healing through miR-21-5p-mediated multiple functions, Biomaterials 308 (2024).
[86] Z. Xiao, S. Chen, K. Zhang, S. Jiang, H. Qin, H. Jian, L. Zhou, Y. Yang, Z. Liu, W. Liu, Y. Tang, J. Luo, J. Liu, Biomimetic bilayer hydrogel loaded with SVF exosomes promotes scar-free healing of diabetic burn wounds, Chem. Eng. J. 520 (2025).
[87] K. Guo, Q. Yu, K. Zhu, Y. Yang, T. Chen, Y. Huang, W. Li, A bioinspired biotin-avidin hydrogel with prolonged SHED Exo delivery enhances diabetic wound healing by modulating the Treg/Th17 balance, Chem. Eng. J. 519 (2025).
[88] C.L. Vipin, G.S.V. Kumar, Exosome laden sprayable thermo-sensitive polysaccharide-based hydrogel for enhanced burn wound healing, Int J Biol Macromol 290 (2025).
[89] Y. Zhang, Y. Xu, W. Hu, X. Ma, J. Hu, Y. Ye, S. Yang, Y. Yu, N. Li, D. Zheng, T. Zhang, H. Lin, J. Gao, An adhesive and self-healing ROS-scavenging hydrogel loading with hMSC-derived exosomes for diabetic wound healing, Int J Pharmaceut 672 (2025).
[90] Y.F. Wang, G. Zhao, S. A, Q. Xu, X.L. Wu, W.X. Wang, Y.J. Rui, In Situ Forming Hypoxia-Induced Exosome-Loaded Hydrogel for Enhanced Diabetic Wound Healing, Macromol Mater Eng (2024).
[91] B. Liu, L. Chen, C. Huang, H. Zhang, H. Zhou, Y. Chen, X. Liu, Z. Xiao, K. Liang, X. Xie, Y. Gao, K. Liu, X. Qi, A sprayable exosome-loaded hydrogel with controlled release and multifunctional synergistic effects for diabetic wound healing, Mater. Today Bio 34 (2025).
[92] H. Yu, B. Wang, Z. Li, K. Liu, W. Chen, S. Zhao, Y. Zhou, G. Wang, Y. Zhou, Y. Chen, H. Chen, Y. Lai, Q. Wang, J. Wang, B. Ni, D. Zhang, C. Pan, Y. He, L. Li, Tβ4-exosome-loaded hemostatic and antibacterial hydrogel to improve vascular regeneration and modulate macrophage polarization for diabetic wound treatment, Mater. Today Bio 31 (2025).
[93] G. Kwak, J. Cheng, H. Kim, S. Song, S.J. Lee, Y. Yang, J.H. Jeong, J.E. Lee, P.B. Messersmith, S.H. Kim, Sustained Exosome-Guided Macrophage Polarization Using Hydrolytically Degradable PEG Hydrogels for Cutaneous Wound Healing: Identification of Key Proteins and MiRNAs, and Sustained Release Formulation, Small 18(15) (2022).
[94] Y. Shi, S. Wang, K. Wang, R. Yang, D. Liu, H. Liao, Y. Qi, K. Qiu, Y. Hu, H. Wen, K. Xu, Relieving Macrophage Dysfunction by Inhibiting SREBP2 Activity: A Hypoxic Mesenchymal Stem Cells-Derived Exosomes Loaded Multifunctional Hydrogel for Accelerated Diabetic Wound Healing, Small 20(25) (2024).
[95] B.S. Subhan, S. Hanson, D. Almanzar, J.F. Cortes Troncoso, P. Katyal, J.W. Sun, H.-W. Shih, T. Mestvirishvili, M. Meleties, F. Arias, A. Wang, K. Ruggles, I. Dolgalev, P. Mita, J.K. Montclare, P.S. Rabbani, Duo-nano exosome encapsulating hydrogel boosts wound healing across xenogenic and allogenic models, Biomaterials (2025).
[96] S.D. Dutta, J.M. An, J. Hexiu, A. Randhawa, K. Ganguly, T.V. Patil, T. Thambi, J. Kim, Y.-k. Lee, K.-T. Lim, 3D bioprinting of engineered exosomes secreted from M2-polarized macrophages through immunomodulatory biomaterial promotes in vivo wound healing and angiogenesis, Bioact. Mater. 45 (2025) 345-362.
[97] L. Jiang, J. Dong, M. Jiang, W. Tan, Y. Zeng, X. Liu, P. Wang, H. Jiang, J. Zhou, X. Liu, H. Li, L. Liu, 3D-printed multifunctional bilayer scaffold with sustained release of apoptotic extracellular vesicles and antibacterial coacervates for enhanced wound healing, Biomaterials 318 (2025).

[98] J. Zou, W. Cui, N. Deng, C. Li, W. Yang, X. Ye, F. Yao, T. Zhang, J. Xiao, C. Ma, L. Wu, D. Dong, J. Chen, C. Guo, A. Liu, H. Wu, Fate reversal: Exosome-driven macrophage rejuvenation and bacterial-responsive drug release for infection immunotherapy in diabetes, J. Control. Release 382 (2025).
[99] T. Chen, L. Ouyang, B. Mi, Y. Sheng, F. Al-Smadi, H. Xu, Y. Wu, X. Zhang, K. Zhou, A. Wu, X. Wang, G. Liu, W. Zhang, MicroRNA-493-5p engineered exosomes delivered via piezoelectric microneedles for epigenetic modulation of macrophages in diabetic wound healing, Mater. Today Bio 37 (2026).
[100] J. Zeng, Z. Sun, F. Zeng, C. Gu, X. Chen, M2 macrophage-derived exosome-encapsulated microneedles with mild photothermal therapy for accelerated diabetic wound healing, Mater. Today Bio 20 (2023).
[101] Y. Cao, B. Chen, Q. Liu, Y. Mao, Y. He, X. Liu, X. Zhao, Y. Chen, X. Li, Y. Li, L. Liu, C. Guo, S. Liu, F. Tan, H. Lu, J. Liu, C. Chen, Dissolvable microneedle-based wound dressing transdermally and continuously delivers anti-inflammatory and pro-angiogenic exosomes for diabetic wound treatment, Bioact. Mater. 42 (2024) 32-51.
[102] J. Gan, X. Zhang, W. Ma, Y. Zhao, L. Sun, Antibacterial, adhesive, and MSC exosomes encapsulated microneedles with spatio-temporal variation functions for diabetic wound healing, Nano Today 47 (2022).
[103] Y. Ding, J. Wang, J. Li, Y. Cheng, S. Zhou, Y. Zhang, Y. Zhao, M. Zhou, Tβ4-Engineered ADSC Extracellular Vesicles Rescue Cell Senescence Through Separable Microneedle Patches for Diabetic Wound Healing, Adv. Sci. 12(26) (2025).
[104] M. Yuan, K. Liu, T. Jiang, S. Li, J. Chen, Z. Wu, W. Li, R. Tan, W. Wei, X. Yang, H. Dai, Z. Chen, GelMA/PEGDA microneedles patch loaded with HUVECs-derived exosomes and Tazarotene promote diabetic wound healing, J Nanobiotechnol 20(1) (2022).
[105] J. Xiang, Z. Li, s. Tseng, T. Li, L. Wang, Z. Li, L. Kang, F. Du, J. Huang, N. Yu, X. Long, Multifunctional MOF microneedle patch with adsorbed exosomes for enhanced diabetic wound healing, Mater. Today Bio 33 (2025).
[106] S. Zhang, T. Jiang, D. Yang, L. Cao, M. Li, J. Tang, Q. Gu, A. Xu, Y. Li, H. Jiang, Dual-domain engineered exosome-based self-powered microneedle delivery platform for the treatment of infected wounds, Mater. Today Bio 35 (2025).
[107] X. Su, J. Yang, Z. Xu, L. Wei, S. Yang, F. Li, M. Sun, Y. Hu, W. He, C. Zhao, L. Chen, Y. Yuan, L. Qin, N. Hu, Fibrous scaffolds loaded with BMSC-derived apoptotic vesicles promote wound healing by inducing macrophage polarization, Genes Dis 12(2) (2025).
[108] G. Kailu, G. Junfeng, Z. Weicheng, Z. Yiqing, X. Yangmengyuan, H. Yaying, X. Zhan, M. Liqian, C. Junli, S. Yejiao, L. Xi, F. Xiaobing, Z. Cuiping, Hypoxia-Challenged sEVs Engineered Nanofiber Scaffolds Accelerate Diabetic Wound Healing via Reversing Cellular Dysfunction of Skin Repair Cells, Research (2026).
[109] H.-T.D. Bui, G. You, M. Lee, W. Mao, C. So, C. Byeon, S. Hong, H. Mok, H.S. Yoo, Milk exosome-infused fibrous matrix for treatment of acute wound, J Control Release 376 (2024) 79-93.
[110] H. Chahsetareh, F. Yazdian, M. Pezeshki-Modaress, M. Aleemardani, S. Hassanzadeh, R. Najafi, S. Simorgh, V. Taghdiri Nooshabadi, Z. Bagher, S.M. Davachi, Alginate hydrogel-PCL/gelatin nanofibers composite scaffold containing mesenchymal stem cells-derived exosomes sustain release for regeneration of tympanic membrane perforation, Int. J. Biol. Macromol. 262 (2024).

[111] F. Ashrafi, A. Emami, S. Sefidbakht, H. Aghayan, F. Soleimani, K. Omidfar, Accelerated healing of full-thickness skin wounds by multifunctional exosome-loaded scaffolds of alginate hydrogel/PCL nanofibers with hemostatic efficacy, Int J Biol Macromol 307 (2025).
[112] J. Mao, S. Qian, Q. Zhao, B. Zhao, B. Lu, L. Zhang, X. Mao, Y. Zhang, W. Cui, X. Sun, Balancing macrophage polarization via stem cell-derived apoptotic bodies for diabetic wound healing, Med 5(2) (2024) 148-168.e8.
[113] W. Li, J. Chen, L. Yu, L. Ding, X. Zhang, L. Yan, M. Shi, Mesenchymal stem cell-derived small extracellular vesicles-loaded GelMA microspheres enhance diabetic wound healing by promoting M2 macrophage polarization through p38 MAPK inhibition, Mater. Today Bio 35 (2025).
[114] P.A. Shiekh, U. Tariq, D.N. Athmuri, P. Singh, J. Bhattacharyya, A. Kumar, N. Bhatnagar, An off-the-shelf bioactive EV delivering cryogel orchestrating immunomodulatory reprogramming and angiogenesis for diabetic ulcer healing, Biomaterials 330 (2026).
[115] H. Zhou, J. Wang, Y. Ding, H. Liang, T. Qin, P. Wang, X. Gao, X. He, Multifunctional copper-based metal-organic frameworks/chitosan derivative/exosome composite sponge for skin healing, iScience 28(12) (2025).
[116] T. Wang, Y. Xue, W. Zhang, Z. Zheng, X. Peng, Y. Zhou, Collagen sponge scaffolds loaded with Trichostatin A pretreated BMSCs-derived exosomes regulate macrophage polarization to promote skin wound healing, Int J Biol Macromol 269 (2024).
[117] G. Kumar, T. Virmani, A. Sharma, B. Goel, N. Chitranshi, M.S. Akhtar, K. Orayj, J.G. Duarte, F. Mascarenhas-Melo, Chitosan-based advanced biomaterials and stimuli-responsive systems for diabetic ulcer management: From principles to translational potential, Carbohyd. Polym. 379 (2026).
[118] Y. Chen, M.R. Younis, G. He, Z. Zheng, Y. Wang, K. Xue, J. Sun, K. Liu, P. Huang, X. Wang, Oxidative Stimuli-Responsive "Pollen-Like" Exosomes from Silver Nanoflowers Remodeling Diabetic Wound Microenvironment for Accelerating Wound Healing, Adv. Healthcare Mater. 12(23) (2023).
[119] H. Zhang, Y. Mao, Z. Nie, Q. Li, M. Wang, C. Cai, W. Hao, X. Shen, N. Gu, W. Shen, H. Song, Iron Oxide Nanoparticles Engineered Macrophage-Derived Exosomes for Targeted Pathological Angiogenesis Therapy, ACS Nano 18(10) (2024) 7644-7655.
[120] Y.X. Lu Enhao, Wang Tao, Huang Xueli, Chen Yiting, Wang Rui, Luo Kuankuan, Zhang Zhiwen, Lin Xiaoxi, Sha Xianyi, Biomimetic Thermo-Sensitive Hydrogel Encapsulating Hemangiomas Stem Cell Derived Extracellular Vesicles Promotes Microcirculation Reconstruction in Diabetic Wounds, Adv. Funct. Mater. 33(45) (2023) 1-15.
[121] H. Meng, J. Su, Q. Shen, W. Hu, P. Li, K. Guo, X. Liu, K. Ma, W. Zhong, S. Chen, L. Ma, Y. Hao, J. Chen, Y. Jiang, L. Li, X. Fu, C. Zhang, A Smart MMP-9-responsive Hydrogel Releasing M2 Macrophage-derived Exosomes for Diabetic Wound Healing, Adv. Healthcare Mater. (2025).
[122] M. Wang, C. Wang, M. Chen, Y. Xi, W. Cheng, C. Mao, T. Xu, X. Zhang, C. Lin, W. Gao, Y. Guo, B. Lei, Efficient Angiogenesis-Based Diabetic Wound Healing/Skin Reconstruction through Bioactive Antibacterial Adhesive Ultraviolet Shielding Nanodressing with Exosome Release, ACS Nano 13(9) (2019) 10279-10293.
[123] Y. Jing, T. Huang, B. Zhao, L. Zhao, N. Zhang, K. Zhang, K. Wang, J. Wang, J. Hua, Q. Tu, A ROS/glucose stimulated-responsive ADSCs-derived exosomes-release hydrogel system for diabetic wound healing, Chem. Eng. J. 487 (2024).
[124] L. Fan, X. Ma, B. Liu, Y. Yang, Y. Yang, T. Ren, Y. Li, Antioxidant-Engineered Milk-Derived Extracellular Vesicles for Accelerating Wound Healing via Regulation of the PI3K-AKT Signaling

Pathway, Adv. Healthcare Mater. 12(32) (2023).
[125] X. Jiang, J. Ma, K. Xue, J. Chen, Y. Zhang, G. Zhang, K. Wang, Z. Yao, Q. Hu, C. Lin, B. Lei, C. Mao, Highly Bioactive MXene-M2-Exosome Nanocomposites Promote Angiogenic Diabetic Wound Repair through Reconstructing High Glucose-Derived Immune Inhibition, ACS Nano 18(5) (2024) 4269-4286.
[126] W. Wang, Y. Huang, X. Chen, Y. Lei, Y. Zheng, X. Shi, J. Yang, Multifunctional conductive hydrogel integrating exosome delivery and electrical stimulation for enhanced diabetic wound healing, Biomater. Adv. 182 (2026).
[127] X. Chen, X. Wang, M. He, J. Kang, J. Gu, Y. Wei, J. Li, B. Li, X. Hong, X. Mao, D. Liu, H. Cai, Mesenchymal Stem Cell-Derived Extracellular Vesicles Embedded in a Self-Adaptive Multifunctional Hydrogel for Rapid Healing of Infected Wounds, Adv. Healthcare Mater. (2025).
[128] J. Lan, L. Shi, W. Xiao, X. Zhang, S. Wang, A Rapid Self-Pumping Organohydrogel Dressing with Hydrophilic Fractal Microchannels to Promote Burn Wound Healing, Adv. Mater. 35(38) (2023).
[129] D. Wu, S. Tao, L. Zhu, C. Zhao, N. Xu, Chitosan Hydrogel Dressing Loaded with Adipose Mesenchymal Stem Cell-Derived Exosomes Promotes Skin Full-Thickness Wound Repair, ACS Appl. Bio Mater. 7(2) (2024) 1125-1134.
[130] Y. Xiong, Z. Lin, P. Bu, T. Yu, Y. Endo, W. Zhou, Y. Sun, F. Cao, G. Dai, Y. Hu, L. Lu, L. Chen, P. Cheng, K. Zha, M.A. Shahbazi, Q. Feng, B. Mi, G. Liu, A Whole-Course-Repair System Based on Neurogenesis-Angiogenesis Crosstalk and Macrophage Reprogramming Promotes Diabetic Wound Healing, Adv. Mater. 35(19) (2023).
[131] J. Li, C. Du, X. Yang, Y. Yao, D. Qin, F. Meng, S. Yang, Y. Tan, X. Chen, W. Jiang, Y. Liu, Instantaneous Self-Healing Chitosan Hydrogels with Enhanced Drug Leakage Resistance for Infected Stretchable Wounds Healing, Small 21(2409641) (2025) 1-17.
[132] X. Zhang, J. Gan, L. Fan, Z. Luo, Y. Zhao, Bioinspired Adaptable Indwelling Microneedles for Treatment of Diabetic Ulcers, Adv. Mater. 35(23) (2023).
[133] L. Zhou, Z. Zeng, J. Liu, F. Zhang, X. Bian, Z. Luo, H. Du, P. Zhang, Y. Wen, Double Bionic Deformable DNA Hydrogel Microneedles Loaded with Extracellular Vesicles to Guide Tissue Regeneration of Diabetes Ulcer Wound, Adv. Funct. Mater. 34(14) (2023).
[134] J. Xu, S. Lin, H. Chen, G. Yang, M. Zhou, Y. Liu, A. Li, S. Yin, X. Jiang, Highly Active Frozen Nanovesicles Microneedles for Senile Wound Healing via Antibacteria, Immunotherapy, and Skin Regeneration, Adv. Healthcare Mater. 13(12) (2024).
[135] S. Meng, Q. Wei, S. Chen, X. Liu, S. Cui, Q. Huang, Z. Chu, K. Ma, W. Zhang, W. Hu, S. Li, Z. Wang, L. Tian, Z. Zhao, H. Li, X. Fu, C. Zhang, MiR-141-3p-Functionalized Exosomes Loaded in Dissolvable Microneedle Arrays for Hypertrophic Scar Treatment, Small 20(8) (2023).
[136] L. Huang, F. Xue, Y. Fu, C. Ma, Q. Wang, H. Ren, Z. Meng, Bioinspired microneedle patch integrating ginger vesicle-silver hybrid nanocomposites with dual antibacterial and angiogenic functions for diabetic wound healing, Mater. Design (2026).
[137] J. Cao, S. Luo, W. Huang, X. Lu, R.K. Kankala, S. Wang, P. Xu, A. Chen, ECM-inspired stem cell secretome sustained releasing composite nanofibrous membranes for accelerated wound healing, Mater. Today Bio 34 (2025).
[138] Y. Zhang, Z. Zhang, X. Jiang, H. Sun, J. Han, H. Wu, Y. Tang, X. Wu, Q. Chen, NIR-responsive Janus dressing with unidirectional exudate drainage and real-time pH monitoring to accelerate infected wound healing, J Mater Sci Technol 270 (2026) 174-186.

[139] L. Shi, X. Liu, W. Wang, L. Jiang, S. Wang, A Self-Pumping Dressing for Draining Excessive Biofluid around Wounds, Adv. Mater. 31(5) (2018).
[140] L. Li, Z. Wang, K. Wang, S. Fu, D. Li, M. Wang, Y. Cao, H. Zhu, Z. Li, L. Weng, Z. Li, X. Ding, L. Wang, Paintable Bioactive Extracellular Vesicle Ink for Wound Healing, ACS Appl. Mater. Interfaces 15(21) (2023) 25427-25436.
[141] S. Vakilian, F. Jamshidi-adegani, F. Al-Fahdi, Z. Mirsanei, J. Al-kindi, K. Al-Riyami, N. Alwahaibi, A. Shalaby, A. Al-Harrasi, N. Al-Sharabi, K. Mustafa, S. Al-Hashmi, Engineered local delivery of extracellular vesicles loaded with si-TNF-α, via a core-sheath 3D-bio-printed scaffold as an effective wound dressing, J Drug Deliv Sci Tec 101 (2024).
[142] L. Chai, J. Huang, M. Wang, Y. Huang, Z. Huang, R. Zhang, L. He, H. Wang, D. Chen, Y. Lei, L. Guo, Injectable deferoxamine-loaded microsphere hydrogels for inhibition of ferroptosis and promotion of third-degree burn wound healing, Mater. Today Bio 32 (2025).
[143] Z. Liu, S. Yang, X. Li, S. Wang, T. Zhang, N. Huo, R. Duan, Q. Shi, J. Zhang, J. Xu, Local transplantation of GMSC-derived exosomes to promote vascularized diabetic wound healing by regulating the Wnt/β-catenin pathways, Nanoscale Adv. 5(3) (2023) 916-926.
[144] H.R.M. Rashdan, E. Tolba, M. Abdelraof, Cotton gauze wound-dressing patches loaded with dapsone derivative and silver nanoparticles for promoting wound healing via antibacterial acceleration function, RSC Adv. 15(56) (2025) 48156-48170.
[145] A.S. Montaser, M. Rehan, W.M. El-Senousy, S. Zaghloul, Designing strategy for coating cotton gauze fabrics and its application in wound healing, Carbohyd. Polym. 244 (2020).
[146] X. Wu, C. Wang, J. Wang, Y. Feng, Y. Zhu, Y. Pan, Y. Yuan, C. Chen, J. Cao, J. Lin, X. Tong, Y. Li, C. Wen, X. Shen, J. Ma, Antiadhesive, antibacterial, and anti-inflammatory sandwich-structured ZIF8-containing gauze for enhanced wound healing, Chem. Eng. J. 491 (2024).
[147] M.R. Faramarzi, M. Sayahi, B. Babamiri, N. Faraji, K. Rahimi, S. Ghaderzadeh, Remarkable healing of a severe wound through surgical debridement, honey medicinal application, amniotic membrane, and bovine collagen powder; A case report study, Int. J. Surg. Case Rep. 132(C) (2025).
[148] B. Babamiri, M.R. Faramarzi, S.K. Taraj, N. Faraji, R. Goli, F. Mohammadi, Healing of a diabetic foot wound through surgical debridement using med-honey, maggot therapy, and human amniotic membrane: A case study, Int. J. Surg. Case Rep. 122(C) (2024).
[149] F.M.A. Altalbawy, B.A.M. Mukhlif, A. Hussen, J.S. Mohammed, R.J. S, A. Singh, S.B. Mishra, A.S. Chauhan, M.E. Astaneh, N. Fereydouni, Regenerative potential of PRP-based scaffolds in chronic wound healing: Mechanisms, advances, and therapeutic insights, Regen. Ther. 30 (2025) 278-298.
[150] Y. Sun, M. Zhu, L. Qiu, Efficacy of mesenchymal stem cells and platelet-rich plasma therapies on wound healing: A Systematic Review and meta-analysis, Regen. Ther. 30 (2025) 75-91.
[151] S. Rüzgar, A. Tabaru, Intraoperative platelet-rich plasma in tonsillectomy: A randomized controlled trial of pain and wound-healing outcomes, Am. J. Otolaryng. 47(3) (2026).
[152] X. Zhang, X. Zhang, L. Wang, L. Zhen, M. Lin, Y. Li, L. Gong, H. Zeng, W. Ruan, M. Zhu, Best evidence summary for platelet-rich plasma treatment of chronic wounds, Regen. Ther. 31 (2026).
[153] Y.-C. Tsai, S.-Y. Wu, C.-J. Wu, H.-Y. Chiao, H.-P. Tseng, Y.-M. He, T.-M. Chen, M.-Y. Tu, Y.-s. Tzeng, Distinct microRNA signatures in Platelet-Rich Plasma-Derived extracellular vesicles predict healing outcomes in chronic diabetic foot ulcers, Diabetes Res. Clin. Pr. 233 (2026).
[154] A. Albelasi, S.A. Alghamdi, M. Alissa, Fibrin hydrogel incorporated with microspheres

containing vascular endothelial growth factor (VEGF) and basic fibroblast growth factor (bFGF) accelerated the healing of diabetic wounds in rats, Tissue Cell 99 (2026).
[155] Z. Jia, S. Li, S. Zhou, C. Su, Q. Liu, L. Lei, B. Xie, Emerging growth factor delivery hydrogel system for wound healing, Mater. Today Adv. 30 (2026).
[156] H. Daneste, L. Mohammadzadeh Boukani, N. Ramezani, F. Asadi, H.K. Zaidan, A. Sadeghzade, M. Ehsannia, A. Azarashk, N. Gholizadeh, Combination therapy along with mesenchymal stem cells in wound healing; the state of the art, Adv Med Sci-Poland 68(2) (2023) 441-449.
[157] B.I. Sukmana, R. Margiana, Y.Q. Almajidi, S.G. Almalki, A. Hjazi, S. Shahab, R.M. Romero-Parra, A.A.A. Alazbjee, A. Alkhayyat, V. John, Supporting wound healing by mesenchymal stem cells (MSCs) therapy in combination with scaffold, hydrogel, and matrix; State of the art, Pathol. Res. Pract. 248 (2023).
[158] F. Rangatchew, P. Vester-Glowinski, B.S. Rasmussen, E. Haastrup, L. Munthe-Fog, M.-L. Talman, C. Bonde, K.T. Drzewiecki, A. Fischer-Nielsen, R. Holmgaard, Mesenchymal stem cell therapy of acute thermal burns: A systematic review of the effect on inflammation and wound healing, Burns 47(2) (2021) 270-294.
[159] M. Mottaghi, C. Kianbakht, S. Samadian, A. Farshbaf, M. Mirhashemi, F. Ahrari, Clinical applications of dental pulp stem cells-derived exosomes in oral and maxillofacial tissue regeneration and repair, Differentiation 146 (2025).
[160] A.Y. Movahed, R. Bagheri, P. Savatier, T. Šarić, S. Moradi, Elimination of tumorigenic pluripotent stem cells from their differentiated cell therapy products: An important step toward ensuring safe cell therapy, Stem Cell Rep. 20(7) (2025).
[161] T.-T.-H. Tran, T.-L. Tran, H.-T.-C. Nguyen, T.-H. Luong, T.-M.-A. Dang, T.-A. Pham, T.-N. Nguyen, A comprehensive review of clinical trials and progress in stem cell therapies for advanced heart failure, Regen. Ther. 30 (2025) 812-837.
[162] I.Z. Sadiq, F.S. Abubakar, B.S. Katsayal, B. Ibrahim, A. Adamu, M.A. Usman, M. Aliyu, M.A. Suleiman, A. Muhammad, Stem cells in regenerative medicine: Unlocking therapeutic potential through stem cell therapy, 3D bioprinting, gene editing, and drug discovery, Biomed. Eng. Adv. 9 (2025).
[163] O. Shadid, J. Novo, R. Saini, I. Seth, P.J. Terrill, W.M. Rozen, Incidence of surgical site infections at skin graft recipient sites with negative pressure wound therapy: A meta-analysis, J. Plast. Reconstr. Aes. 110 (2025) 306-321.
[164] A. Watters, M. Maloney, G. Brown, P. Socrates, D. Russell, E. Batko, S. Burgwardt, S. Ahmed, M. Thirunavukkarasu, M.M. Sanders, J.A. Palesty, N. Maulik, Overexpression of thioredoxin-1 enhances angiogenesis and survival in a mouse ischemic skin flap model, Surgery 194 (2026).
[165] C. Seebart, H. Kunzman, G. Cole, S. Chen, Application of acellular fish skin grafts for complicated soft tissue wound management in multiple exotic companion animal species: 3 cases (2023-2024), J. Exot. Pet Med. (2026).
[166] M. Alissa, S.A. Alghamdi, A.S. Binshaya, Hyaluronic acid-based three dimensional scaffold in combination with hyperbaric oxygen therapy promote diabetic wound healing, Tissue Cell 98 (2026).
[167] A.R. Khalatbary, S. Sarabandi, F. Ahmadi, F.M. Kasmaie, N. Sadeghi, S. Soleimani, R.A. Disfani, A. Raoofi, D. Nasiry, Transplantation of bioengineered dermal derived matrix-scaffold in combination with hyperbaric oxygen therapy improves wound healing in diabetic rats, Tissue Cell

89 (2024).
[168] F. Shen, D. Phuyal, B.B. Ozmen, V. Ferrini, E.F.R. Duraes, R.S. Djohan, Hyperbaric oxygen therapy for complex wound management following breast cancer treatment: Single institution 10-year experience, J. Plast. Reconstr. Aes. 111 (2025) 218-225.
[169] E. Pathault, S. Sanchez, B. Husson, C. Vanhaecke, P. Georges, C. Brazier, B. Mourvillier, M. Viguier, Hyperbaric oxygen therapy enables pain reduction and healing in painful chronic wounds, including in calciphylaxis, Ann. Dermatol. Vener. 151(4) (2024).
[170] M. Drabik, L.H. Granicka, Advances in wound healing: physiology, complications, the role of oxygen and innovative treatment strategies enhancing oxygenation, Biocybern. Biomed. Eng. 46 (2026) 113-129.
[171] B. Babamiri, F. Nikkhah, N. Faraji, R. Goli, N.V. Moghaddam, K. Rahimi, Diabetic foot ulcer: Successful healing with combination therapy, including surgical debridement, maggot therapy, and negative pressure wound therapy, Int. J. Surg. Case Rep. 110 (2023).
[172] C. Arundel, L. Mandefield, C. Fairhurst, K. Baird, A. Gkekas, P. Saramago, I. Chetter, B. Corbacho Martin, C.E. Hewitt, A. Mott, S. Swan, D.J. Torgerson, J. Wilkinson, S. Zahra, J. Blazeby, R. Macefield, S. Dixon, J. Hatfield, A. Oswald, J. Dumville, M. Lee, T. Pinkney, N. Stubbs, L. Wilson, A. Clothier, D.C. Bosanquet, M. Blow, C. Price, J. Todd, T. Munro, W. Pillay, A. Pradhan, A. Garnham, M. Wall, K. Powezka, A. Syed, D. Gerrard, A. Croucher, N. Hadjievangelou, A. Firth, T. Roe, G. Smith, C. Bicknell, C. Carr, E. Negbenose, L. Tarusan, A. Vesey, D. Wilson, D. Bell, J. Fletcher, C. Greenwood, T. Wallace, S. Vallabhaneni, S. Holder, J. Williams, S. Sim, A.L. Tambyraja, F. Kerray, A. Ng, M. Sylvester, L. Slater, S.T. Rashid, A. Palacios, K. Feld, S. Nandhra, G. Stansby, N. Parr, L. Jones, J. Milne, C. Stubbs, R. Hinchliffe, C. Twine, G.A. Antoniou, C. Corbett, S. Munt, S. Warran, R. Fletcher, W. Al-Jundi, M. Burrows, P. Stather, R. Barnes, T. Woodrow, B. Adams, O. Agu, Y. Gleeson, R. Dsouza, L. Erete, S. Jones, C. Checketts, D. Bajic, R. Matravers, I. Loftus, J. Budge, B. Azhar, R. Hancox, C. Pearce, N. Suggett, A. Whitehouse, M. Juszczak, G. Kuhan, S. Premnath, N. Dattani, V. Hollings, F. Khasawneh, J. AlShakarchi, E. Packer, Negative pressure wound therapy versus usual care in patients with surgical wound healing by secondary intention in the UK (SWHSI-2): an open-label, multicentre, parallel-group, randomised controlled trial, The Lancet 405(10490) (2025) 1689-1699.
[173] M. Abudurezhake, Y. Huang, H. Wang, G. Aili, Y. Xu, Y. Tian, Z. Ma, The role of negative pressure wound therapy—From the perspective of drainage fluid composition, Surgery Open Sci. 30 (2026) 23-32.
[174] Y. Zeng, J. Tu, J. Qin, The mechanism by which negative pressure wound therapy promotes wound healing, J. Tissue Viability 35(1) (2026).
[175] N. Pan, M. Zhang, X. Fang, Y. Liu, Q. Meng, Y. Li, Patient voices from the chronic wound care journey: A meta-synthesis of experiences with negative pressure therapy, J. Vasc. Nurs. (2026).
[176] Y. Jiang, L. Yan, M. Liu, Y. Guo, K. Tang, A polyvinyl alcohol-gelatin composite hydrogel dressing for accelerated wound healing under electrical stimulation, Colloid Surface B 263 (2026).
[177] X. Lin, L. Yang, T. Xu, B. Cheng, H. Liu, Smart dressings for wound monitoring and treatment: a review with focus on electrical stimulation therapy, Microchem. J. 223 (2026).
[178] M. Verdes, K. Mace, L. Margetts, S. Cartmell, Status and challenges of electrical stimulation use in chronic wound healing, Curr. Opin. Biotech. 75 (2022).
[179] W. Wang, F. Sun, F. Yang, C. Cui, B. Wang, Y. Huang, X. Zhang, Z. Yang, Synergistic

supramolecular conductive dressing integrating electrical stimulation and polyoxometalate antioxidants for enhanced chronic wound healing, Acta Biomater. 204 (2025) 340-353.
[180] Y. Wang, Z. Cao, Q. Wei, K. Ma, W. Hu, Q. Huang, J. Su, H. Li, C. Zhang, X. Fu, VH298-loaded extracellular vesicles released from gelatin methacryloyl hydrogel facilitate diabetic wound healing by HIF-1α-mediated enhancement of angiogenesis, Acta Biomater. 147 (2022) 342-355.
[181] L. Tian, Z. Wang, S. Chen, K. Guo, Y. Hao, L. Ma, K. Ma, J. Chen, X. Liu, L. Li, X. Fu, C. Zhang, Ellagic Acid-Loaded sEVs Encapsulated in GelMA Hydrogel Accelerate Diabetic Wound Healing by Activating EGFR on Skin Repair Cells, Cell Prolif. (2025).
[182] Z. Liu, D.-H. Chen, Z.-H. Lin, Z.-Y. Wang, H. Peng, R.-T. Liu, Z.-C. Hu, Y.-H. He, X.-J. Wei, C.-Q. Zhang, Y. Feng, Q. Tang, Z.-Z. Zhu, In-situ Sprayed platelet-derived small extracellular vesicles for the skin flap survival by reducing PANoptosis, Biomaterials 316 (2025).
[183] L. Chen, K. Peng, H. Huang, Z. Gong, J. Huang, A.M. Mohamed, Q. Chen, W.T. Sow, L. Guo, K.Y.H. Kwan, B. Li, M.A. Khan, P. Makvnadi, M. Jones, S. Shen, X. Wang, C. Ma, H. Li, A. Wu, Injectable Hydrogel Based on Enzymatic Initiation of Keratin Methacrylate for Controlled Exosome Release in Intervertebral Disc Degeneration Therapy, Adv. Funct. Mater. 34(32) (2024).
[184] Z. Xing, C. Zhao, S. Wu, D. Yang, C. Zhang, X. Wei, X. Wei, H. Su, H. Liu, Y. Fan, Hydrogel Loaded with VEGF/TFEB-Engineered Extracellular Vesicles for Rescuing Critical Limb Ischemia by a Dual-Pathway Activation Strategy, Adv. Healthcare Mater. 11(5) (2021).
[185] M. He, Y. Wei, X. Chen, J. Li, J. Kang, B. Li, D. Liu, H. Cai, Incorporating Stem Cell-Derived Small Extracellular Vesicles into a Multifunctional OHAMA-EPL Hydrogel Spray for Rapid Healing of Infected Skin Wounds, Adv. Healthcare Mater. 14(32) (2025).
